# Domain-dependent uncoupling of the co-chaperone and E3 ubiquitin ligase CHIP underlies heterogeneity in spinocerebellar ataxia 48

**DOI:** 10.1016/j.jbc.2026.113399

**Published:** 2026-08-07

**Authors:** Selin Altinok, Ethan Paulakonis, Michael F. Almeida, Isaac Hwang, Rebekah Sanchez-Hodge, Christina D’Ovidio, Alison S. Eidman, Shreesti Shrestha, Georgia Roper, Grant Irons, Elena Vargas, Ivy Peng, Morcos Saeed, Aliyaa Pathan, Mel N. Azin, Joseph Latham, Sarah M. Ronnebaum, Chang-he Shi, Mark J. Ranek, K. Matthew Scaglione, Richard C. Page, Nicholas G. Brown, Jonathan C. Schisler

**Affiliations:** 1 The McAllister Heart Institute, The University of North Carolina at Chapel Hill, Chapel Hill, North Carolina, USA; 2 Department of Pharmacology, The University of North Carolina at Chapel Hill, Chapel Hill, North Carolina, USA; 3 Department of Chemistry and Biochemistry, Miami University, Oxford, Ohio, USA; 4 Institute of Neuroscience, Zhengzhou University, Zhengzhou, Henan, China; 5 Division of Cardiology, Department of Medicine, Johns Hopkins Medical Institutions, Baltimore, Maryland, USA; 6 Department of Molecular Genetics and Microbiology, Duke University, Durham, North Carolina, USA; 7 Department of Pathology and Lab Medicine, and Computational Medicine Program, The University of North Carolina at Chapel Hill, Chapel Hill, North Carolina, USA

**Keywords:** CHIP, co-chaperone, E3 ubiquitin ligase, genotype-phenotype correlation, heat shock response, HSF1, HSP70, STUB1, molecular chaperone, oligomerization, protein quality control, spinocerebellar ataxia type 48

## Abstract

The carboxyl terminus of Hsp70-interacting protein (CHIP, encoded by *STUB1*) combines co-chaperone and E3 ubiquitin ligase activities to regulate protein quality control. Heterozygous mutations in *STUB1* cause spinocerebellar ataxia type 48 (SCA48), a progressive cerebellar ataxia with variable extrapyramidal and cognitive features. To understand the molecular basis of this variability, we systematically analyzed 13 SCA48-associated variants spanning the TPR and U-box domains through recombinant protein biochemistry and cellular models. TPR variants retained intrinsic ligase activity but showed significantly reduced HSP70 binding, impaired substrate ubiquitination, and decreased stability. Conversely, U-box variants abolished ligase function, promoted the formation of high-molecular-weight oligomers, and often increased CHIP levels while only partially impairing co-chaperone activity. Many mutants displayed temperature-sensitive defects and defective stress-induced nuclear translocation. Principal component analysis revealed distinct biochemical clustering specific to each domain. RNA-seq following *STUB1* knockdown modeled CHIP insufficiency and showed preserved HSF1-dependent transactivation, but loss of CHIP’s capacity to amplify ubiquitination, chaperone function, and stress-related transcriptional programs. Meta-analysis of 87 SCA48 patients linked TPR-like biochemical signatures to upper motor neuron involvement and U-box-like profiles to prominent dysarthria. Overall, these data indicate that SCA48 results from domain-specific disruption of CHIP’s dual functions, producing varying degrees of CHIP insufficiency and/or gain-of-toxic effects that together contribute to the phenotypic diversity observed across patients. This work refines the mechanistic framework for SCA48 pathogenesis and highlights strategies for therapeutic modulation of residual CHIP activity.

Spinocerebellar ataxias (SCAs) are a group of hereditary ataxias characterized by progressive gait incoordination and are often associated with impaired coordination of the hands, speech, and eye movements. Although the genetic causes involve mutations in various proteins, a common feature of SCAs is the inability to maintain proper protein homeostasis under stress. Therefore, strong protein quality control (PQC) responses and their machinery are vital for cellular function (1).

The gene *STUB1* (*STIP1* homology and U-box containing protein 1) encodes the PQC protein known as CHIP (Carboxyl terminus of HSC70-interacting protein). Importantly, mutations in *STUB1* can cause a wide range of clinical symptoms, including classic ataxia, developmental delay, cognitive decline, accelerated aging, and hypogonadism. The first disease-related mutation in *STUB1* was identified in patients with a newly classified recessive form of spinocerebellar ataxia, SCAR16 (2, 3). In 2018, a heterozygous *STUB1* mutation was linked to a new autosomal dominant spinocerebellar ataxia, SCA48 (4). Additionally, *STUB1* mutations have been shown to modify other ataxias, such as SCA8 and SCA17 (5, 6). These findings collectively highlight a unique role for *STUB1* as both a driver and modifier of SCAs, emphasizing its importance in the development of these disorders.

CHIP plays a dual role in the PQC pathway as a co-chaperone with heat shock proteins (HSPs) and as a ubiquitin ligase, helping proteins fold and be targeted for degradation, respectively (7, 8, 9, 10). CHIP’s multiple enzymatic functions highlight its importance in the cellular stress response, especially in neurodegenerative diseases (11, 12, 13, 14, 15). Understanding how SCA-related mutations disable CHIP's various functions is therefore crucial, as it highlights CHIP’s potential as a therapeutic target given its ability to ubiquitinate and degrade disease-related proteins *via* the proteasome (16).

CHIP uses TPR (tetratricopeptide) and U-box domains to perform its enzymatic functions. In brief, the N-terminal TPR domain binds to chaperones, such as HSC70 (constitutive form; encoded by *HSPA8*) and HSP70 (inducible form; *HSPA1A*), for protein refolding and recruits substrates for ubiquitination. CHIP’s inherent ubiquitin (Ub) ligase activity is enabled by the U-box domain, which binds and activates E2 Ub-conjugating enzymes, like UBE2D2. The co-recruitment of substrates to its TPR and the E2∼Ub (“∼” indicates a transient thioester intermediate) to its U-box facilitates polyubiquitination. Synthetic mutations in each domain, such as TPR (K30A) and U-box (H260Q), have been used previously to investigate their functions. In addition to its enzymatic roles, CHIP naturally forms stable dimers and higher-order oligomers, such as tetramers and hexamers, that function in the heat shock response (17, 18, 19). Prior studies have shown that SCAR16 disease-related mutations destabilize CHIP, resulting in a loss of co-chaperone or ubiquitin ligase activity (20). Additionally, a well-characterized SCA48 mutation in the TPR domain (A52G) was defective in recruiting HSP70 (21). However, other SCA48-linked mutations occur in both the TPR and the U-box domains of CHIP (22, 23), which may impair CHIP’s ubiquitin ligase activity and/or alter its oligomeric state, potentially contributing to disease symptoms.

Despite the critical role of CHIP in maintaining protein homeostasis and its involvement in various neurodegenerative diseases, there are currently no effective therapies targeting *STUB1* mutations. Therefore, understanding the molecular mechanisms behind these mutations is essential for developing targeted treatments. Ultimately, our findings reveal that mutations cause a complex range of changes in protein stability, oligomerization, and enzymatic activity, depending on their location. Furthermore, these biochemical changes are associated with clinical manifestations in SCA48 patients.

## Results

### SCA48 mutations have different effects on CHIP protein expression levels

**Figure 1:**
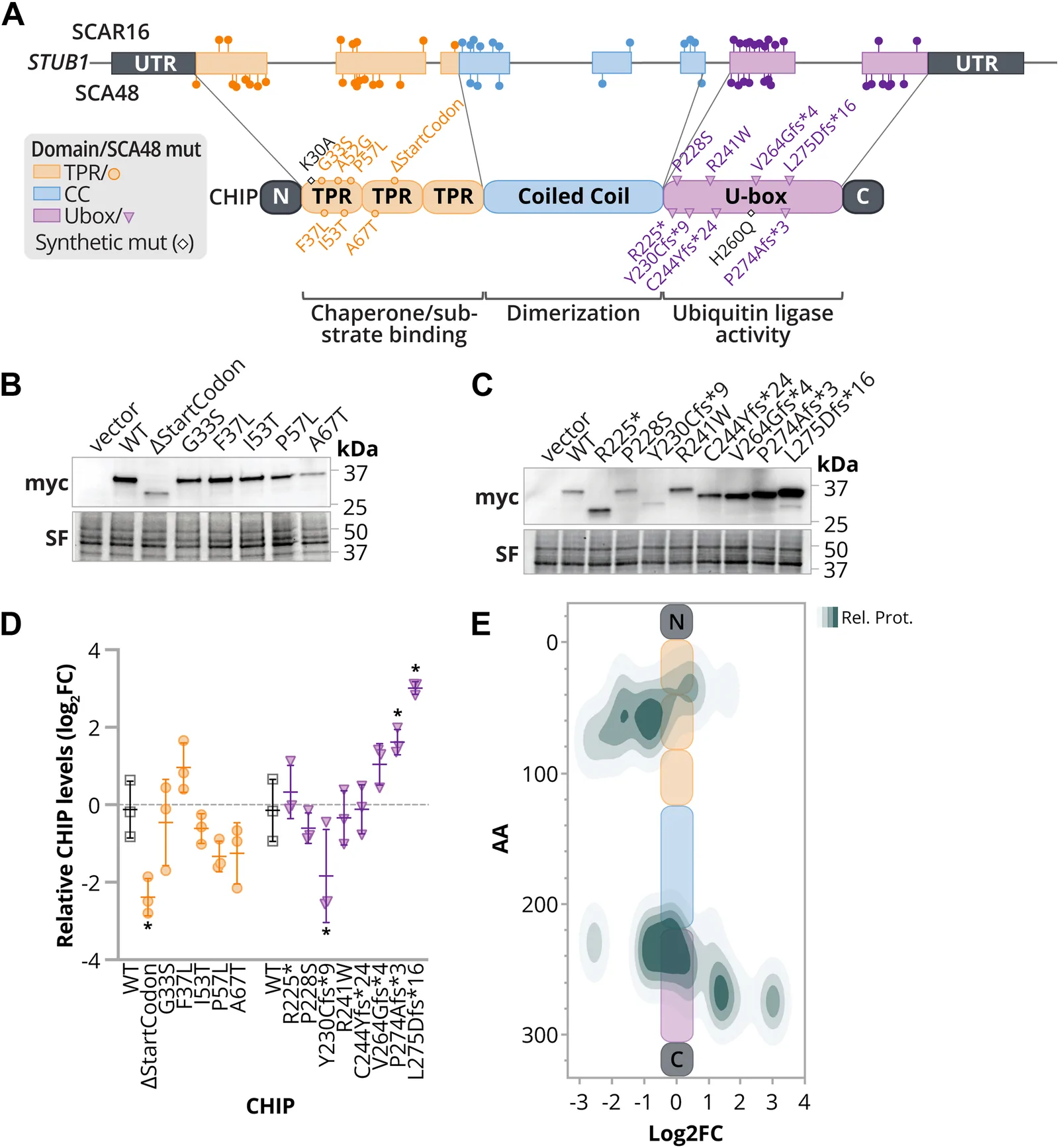
**Distinct CHIP protein expression patterns of SCA48-linked mutations.***A*, gene diagram (*upper*) showing *STUB1* mutations associated with recessive SCAR16 (*top*) and dominant SCA48 (*bottom*). Selected SCA48 mutations and the resulting location in the encoded CHIP protein (*lower*) are highlighted, along with synthetic mutations (K30A and H260Q). *B & C*, Representative SDS-PAGE immunoblots of steady-state protein levels of the indicated myc-tagged CHIP protein transiently expressed in COS-7 with stain-free (SF) levels of total protein. *D*, densitometry quantification of normalized CHIP protein levels expressed as log_2_ fold change relative to wild-type (WT). Data are presented as mean ± SD from three independent transfections, each performed with separate plasmid DNA preparations of equal quality and amount per cell. Transfection efficiency was monitored in parallel using a GFP reporter, with >75% efficiency serving as the minimum acceptance threshold. Statistical analysis employed a mixed-effects model (F(14, 12.43) = 4.9 × 10^−9^), with Dunnett’s *post hoc* test for multiple comparisons against WT (∗*p* < 0.05). Similar patterns were observed for SCAR16 mutations in prior studies (20, 81). *E*, density heatmap plotting protein levels (Rel. Prot.) relative to WT CHIP and the amino acid (AA) position of the mutation.

SCA48 mutations encompass both single-amino-acid substitutions and protein-domain deletions. Therefore, we selected a subset of 13 SCA48-associated mutations within the TPR and U-box domains for detailed analysis, including seven missense, five frameshift, and one nonsense mutation (22, 23). We also examined a *STUB1* mutation in the start codon, c.3G>A, which results in a truncated TPR domain due to an in-frame alternative translation start site within the second exon (denoted as ΔStartCodon) (24) (Fig. 1*A*).

First, we evaluated CHIP’s steady-state levels in a cell culture system by transiently transfecting COS-7 cells with constructs containing different CHIP mutations. By assessing protein levels *via* immunoblotting, we observed that TPR domain mutants either showed no change or a trend toward reduced protein expression. For example, the ΔStartCodon, which truncates the TPR domain, was markedly decreased, likely due to protein misfolding. Conversely, U-box mutants exhibited varying protein expression levels: one mutation led to decreased expression, whereas others showed either unchanged or markedly increased steady-state expression compared to CHIP WT (Fig. 1, *B*–*D*, Table S1). This wide range of protein expression suggests that distinguishing between changes in protein function and changes in protein levels is difficult solely in cell-based systems. However, plotting the relative change in expression along the primary structure of CHIP reinforced a trend of lower expression in TPR mutants and higher expression in U-box mutants, suggesting that the stability contributed by these domains may differ in cells (Fig. 1*E*).

### Recombinant systems exhibit notable differences in protein stability and oligomerization

The variable protein levels among SCA48-harboring mutations could result from changes in CHIP's intrinsic properties, such as stability, oligomerization, enzymatic activity, or a combination of these factors. For instance, defects in CHIP’s ubiquitination activity might stabilize the steady-state protein levels by decreasing autoubiquitination and degradation. To investigate these fundamental characteristics in an isolated, cell-free system, we purified recombinant forms of both wild-type and CHIP disease variants to perform a series of *in vitro* assays.

**Supporting Figure S1:**
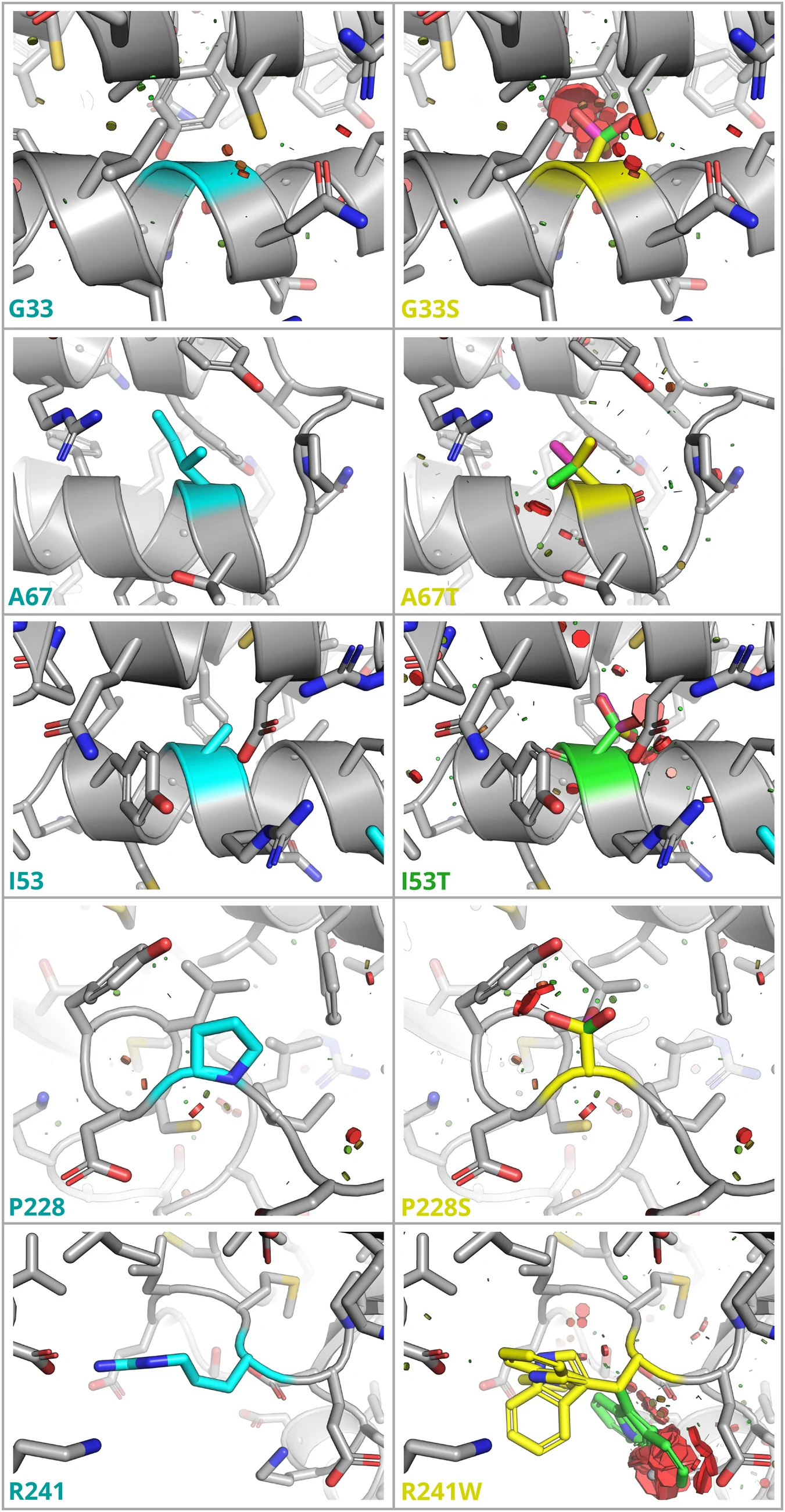


Given the dominant inheritance pattern of SCA48, we hypothesized that SCA48 mutations would be as stable or more stable than wild-type CHIP. Therefore, to assess protein stability, we initially performed differential scanning fluorimetry on recombinant CHIP proteins. These results did not show a consistent trend in thermal stability among SCA48 proteins. Instead, we observed varied effects across different variants (Table 1). For example, while P57L and Y230Cfs∗9 showed increased melting temperatures, the A67T and R241W mutations significantly lowered the melting temperature of CHIP, indicating stabilization and destabilization, respectively. Several variants with lower melting temperatures, such as G33S, I53T, A67T, and R241W, also exhibited predicted steric clashes when modeled on the CHIP dimeric structure (Fig. S1). These changes in protein stability may help explain the lower steady-state levels observed for some CHIP variants.

**Table 1:** Melting temperatures of SCA48-linked CHIP mutations

CHIP	Mean	StDev	Dif	*p*
WT	44.12	0.60	n/a	n/a
G33S	41.40	1.09	−2.72	0.1054
F37L	44.87	0.91	0.75	0.9949
I53T	41.78	1.24	−2.33	0.2170
P57L	48.18	1.78	4.07	**0.0052**
A67T	40.50	4.58	−3.62	**0.0149**
R225∗	47.00	0.78	2.88	0.0760
P228S	42.74	0.14	−1.38	0.7794
Y230Cfs∗9	47.29	0.62	3.17	**0.0406**
R241W	40.80	0.72	−3.32	**0.0296**
C244Yfs∗24	42.48	0.77	−1.63	0.6083
P274Afs∗3	45.15	1.38	1.03	0.9489
L275Dfs∗16	44.47	0.23	0.35	1.0000
V264Gfs∗4	42.79	0.22	−1.33	0.8106

The mean and standard deviation (StDev) from three independent melting experiments of the indicated recombinant CHIP protein. Mixed model analysis of melting temperatures, using replicate number as a random factor, was used to compare the differences relative to wild-type (WT) using Dunnett’s multiple test corrected *p* values (*p*).

Bold *p* values indicate melting temperatures significantly different from WT (*p* < 0.05).

**Figure 2:**
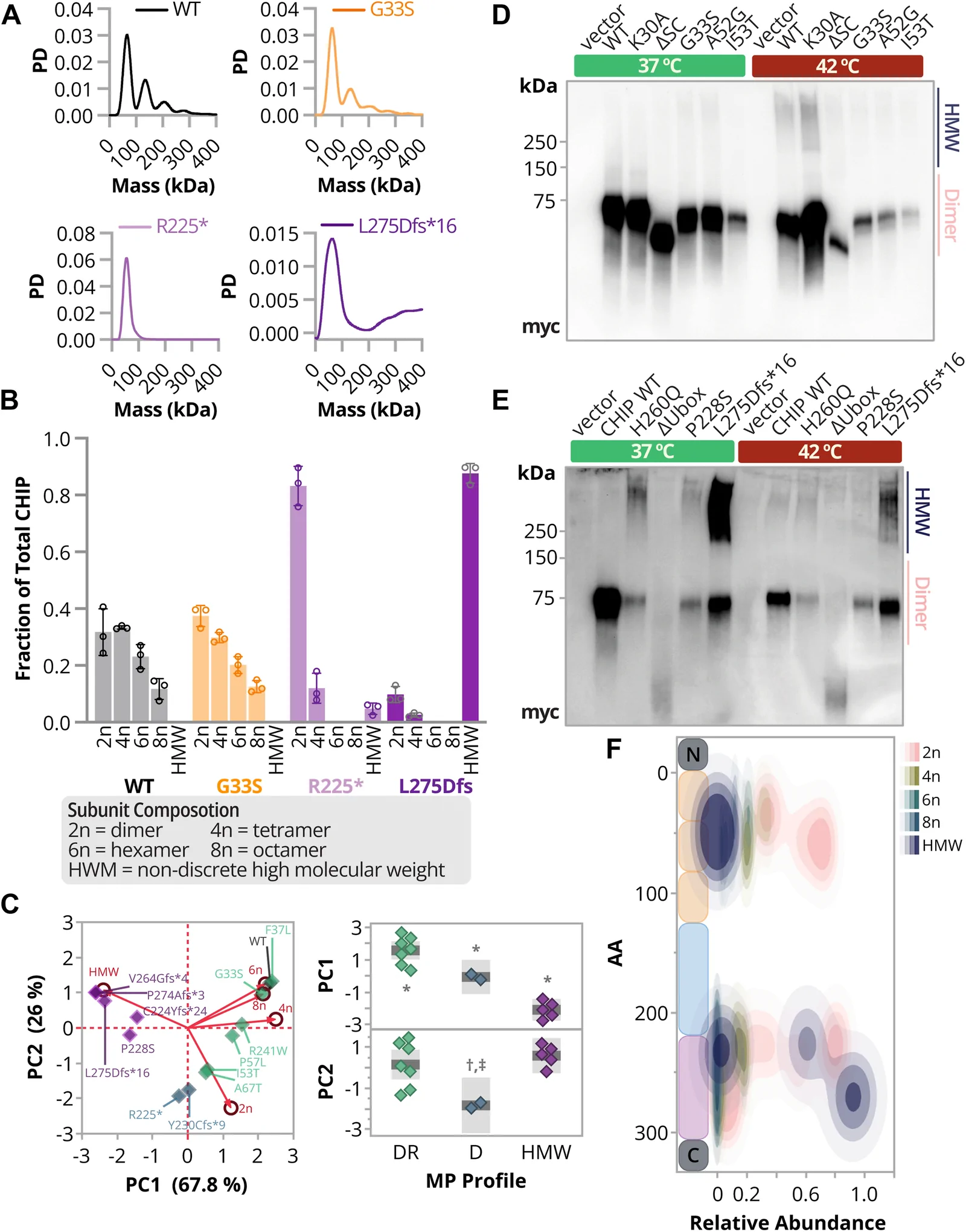
**Effect of SCA48-associated mutations on CHIP oligomeric state.***A*, probability distribution (PD) *versus* molecular mass (kDa), determined by mass photometry, of WT CHIP and three SCA48 mutant proteins that represent the main patterns of CHIP oligomerization. Full plots and additional data are present in Fig. S2. *B*, the proportions of CHIP oligomeric states, dimer (2n), tetramer (4n), hexamer (6n), octamer (8n), and high molecular weight (HMW), are represented by a bar graph and summarized as the mean ± SD (n = 3). The fraction of CHIP in each oligomeric state is weighted based on the number of monomeric subunits in each oligomer. *C*, oligomeric variation of CHIP proteins represented by (*left*) scatterplot of the first two principal components (PC) with the loadings (○) represented by red arrows and proteins (represented by diamonds) colored by mass photometry (MP) profile, and (*right*) dot plot of MP profiles across PC1 (*top*) and PC2 (*bottom*). Oneway ANOVA of PC1 and PC2, F(2,11) = 43, *p* < 0.0001, and F(2,11) = 5.7, *p* = 0.0201. Tukey posttest: ∗*p* < 0.05 of all pairwise comparisons, †*p* < 0.05 D *versus* DR, and ‡*p* < 0.05 D *versus* HMW. The dark line represents the mean, and the shaded region represents the 95% confidence interval. *D & E*, immunoblot analysis of CHIP oligomerization in COS-7 cells transfected with the indicated plasmids using native blue PAGE, under either 37 °C or 42 °C conditions, with dimer and HMW species indicated. Molecular weights annotated on the BN-PAGE blots correspond to the NativeMark ladder run alongside the samples and delineate the migration range of the resolved species, which are annotated as dimer *versus* non-discrete high-molecular-weight (HMW) forms. These panels provide an independent, cellular corroboration of the oligomeric assignments established by mass photometry (*A*-*B* and Figs. S2 and S3); band positions are not intended as precise molecular weight determinations for individual oligomeric species. ΔStartCodon is abbreviated as ΔSC. *F*, density heatmap showing amino acid (AA) positions and the resulting abundance of CHIP oligomeric species.

**Supporting Figure S2:**
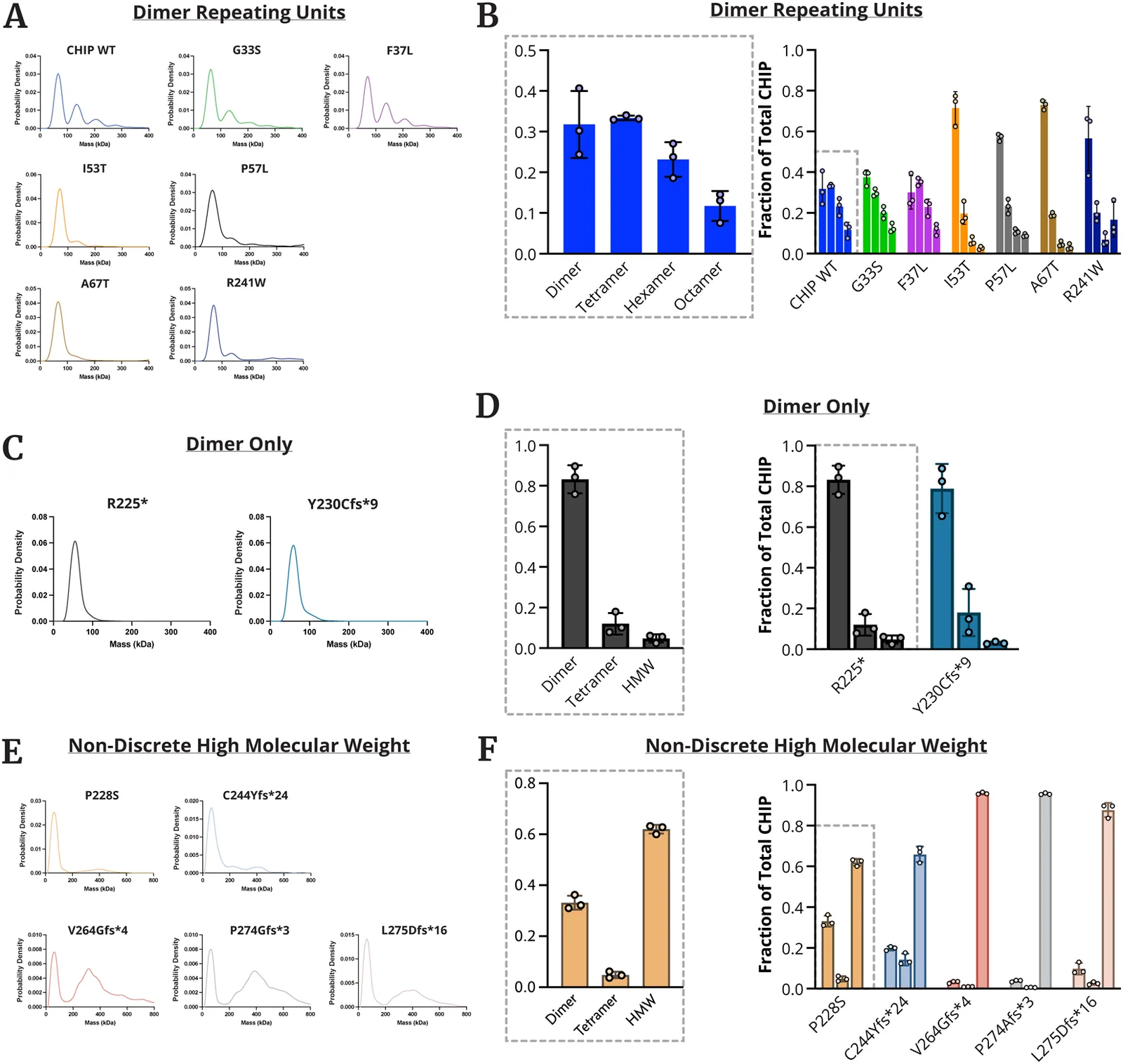


**Supporting Figure S3:**
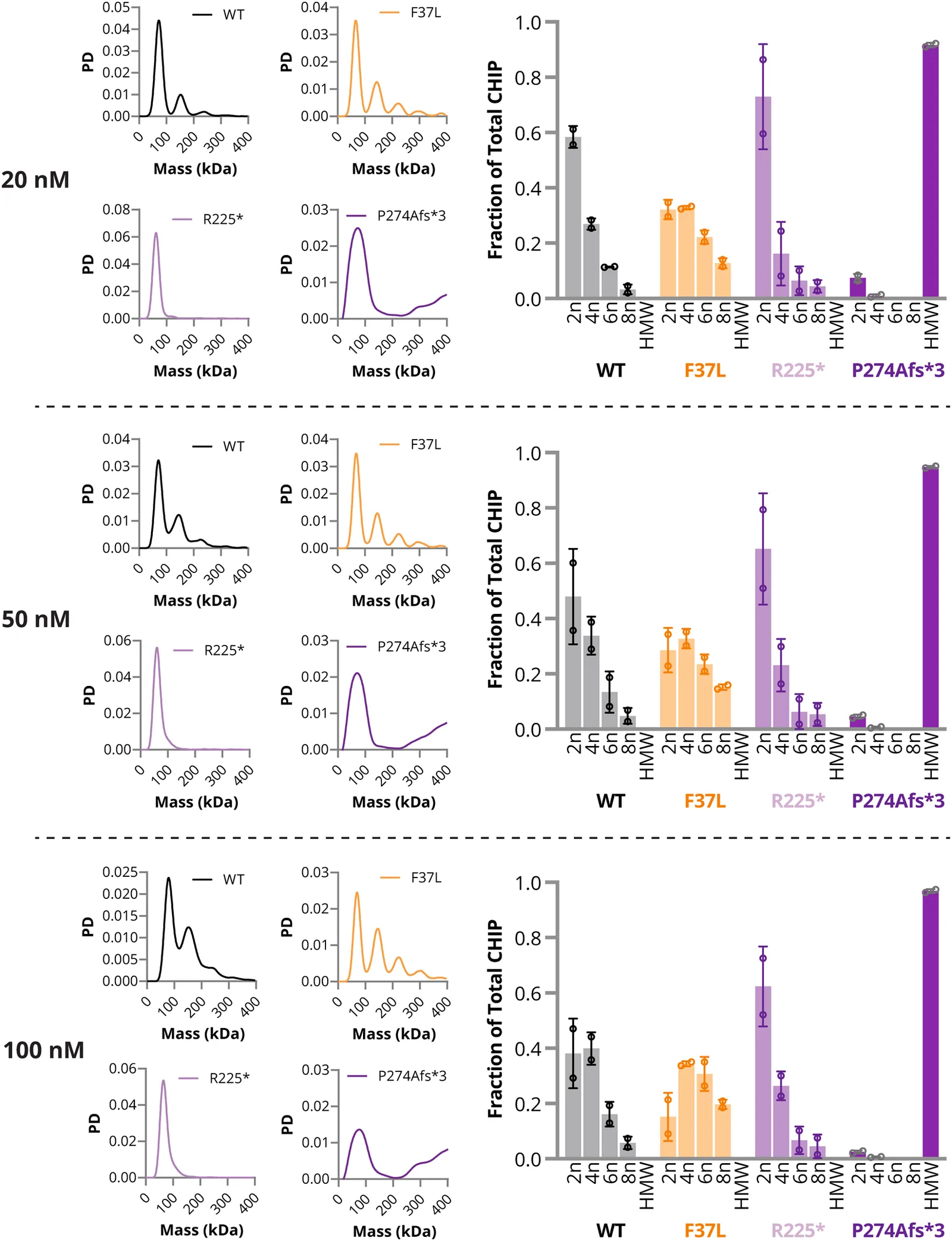


Next, we used mass photometry (MP) to monitor the oligomerization of CHIP wild type and variants, as an increased propensity for oligomerization could represent a mechanism by which certain mutations alter enzymatic activity. We observed distinct peaks at molecular weights corresponding to dimer, tetramer, hexamer, and octamer with CHIP WT (Fig. 2, *A* and *B*). All TPR domain mutants showed clear peaks matching all four oligomeric states (Figs. 2, *A*, *B* and S2, *A*, *B*). The R241W variant was the only U-box domain mutant that oligomerized similarly to CHIP WT (Fig. S2, *A* and *B*). Interestingly, about 80% of R225∗ and Y230Cfs∗9 proteins existed as dimers, lacking clear higher-order states such as hexamer and octamer (Figs. 2, *A*, *B* and S2, *C*, *D*). Conversely, the other U-box mutants mainly formed large, non-distinct high-order oligomers over 200 kDa, with minimal dimer presence (Figs. 2, *A*, *B* and S2, *E*, *F*). Based on their MP profiles, we used principal component analysis to categorize SCA48-associated CHIP mutations into three groups (Fig. 2*C*) (1): dimer repeating units (DR), which show distinct oligomeric states at dimer, tetramer, hexamer, and octamer (2); dimer-only (D), for mutations with approximately 80% dimer formation and no clear high oligomeric state; and (3) non-discrete high molecular weight (HMW), representing mutants that produce very low levels of distinct oligomers and are mostly found as non-distinct high-order oligomers (Table 2). These classifications were not altered when a representative set of CHIP variants was tested at multiple concentrations by MP (Fig. S3).

**Table 2:** MP profile of CHIP mutations

CHIP	MP profile
WT	1
G33S	1
F37L	1
I53T	1
P57L	1
A67T	1
R225∗	2
P228S	3
R241W	1
Y230Cfs∗9	2
C244Yfs∗24	3
V264Gfs∗4	3
P274Afs∗3	3
L275Dfs∗16	3

Classification of oligomeric status: 1 - Dimer repeating units, 2 - Dimer only, and 3 - Non-discrete high molecular weight.

The oligomeric state of proteins often influences their function, especially during cellular stress. For example, CHIP forms high-order oligomers after heat shock, and its chaperone activity increases with heat, indicating that mutations affecting oligomerization can impact CHIP’s function (17). Therefore, we aimed to identify changes in the oligomeric state of CHIP wild-type and of CHIP with SCA48 mutations in a cellular system following heat shock. COS-7 cells were transfected with CHIP wild-type and mutant constructs, and cells were lysed under non-denaturing conditions to preserve native structure and interactions. CHIP oligomerization was analyzed using Blue Native-PAGE followed by immunoblotting. At physiological temperature, CHIP WT and all TPR mutants mainly migrated at the molecular weight of a CHIP dimer (70 kDa) (Fig. 2*D*), consistent with MP (Fig. 2, *A* and *B*). When subjected to heat shock at 42 °C, high-molecular-weight species of CHIP WT and K30A formed, while disease-related TPR mutants failed to form these higher-molecular-weight forms (Fig. 2*D*). Consistent with earlier MP data, U-box mutants appeared as high-order oligomers larger than 200 kDa in COS-7 cells at physiological temperature (Fig. 2*E*). However, after heat shock, there was a reduction in higher-molecular-weight forms of U-box-mutant proteins (Fig. 2*E*). Mapping MP-derived oligomeric status of the mutations across the primary structure of CHIP again reinforces domain-specific trends in oligomerization (Fig. 2*F*).

Along with MP data, these results show consistent changes in the oligomeric state of CHIP, which may alter its enzymatic and co-chaperone activities. In its monomer form, CHIP is considered inactive (25). Asymmetric dimerization of two protomers exposes the E2-binding region of the U-box domain, thereby making it accessible to mediate substrate ubiquitination (25, 26). Since these data indicate differences in CHIP's biophysical properties, changes in oligomerization and protein-protein interactions could also affect its enzymatic ubiquitin-ligase and co-chaperone functions.

### SCA48 mutations in the TPR and U-box differentially impact CHIP-dependent ubiquitination activity

**Figure 3:**
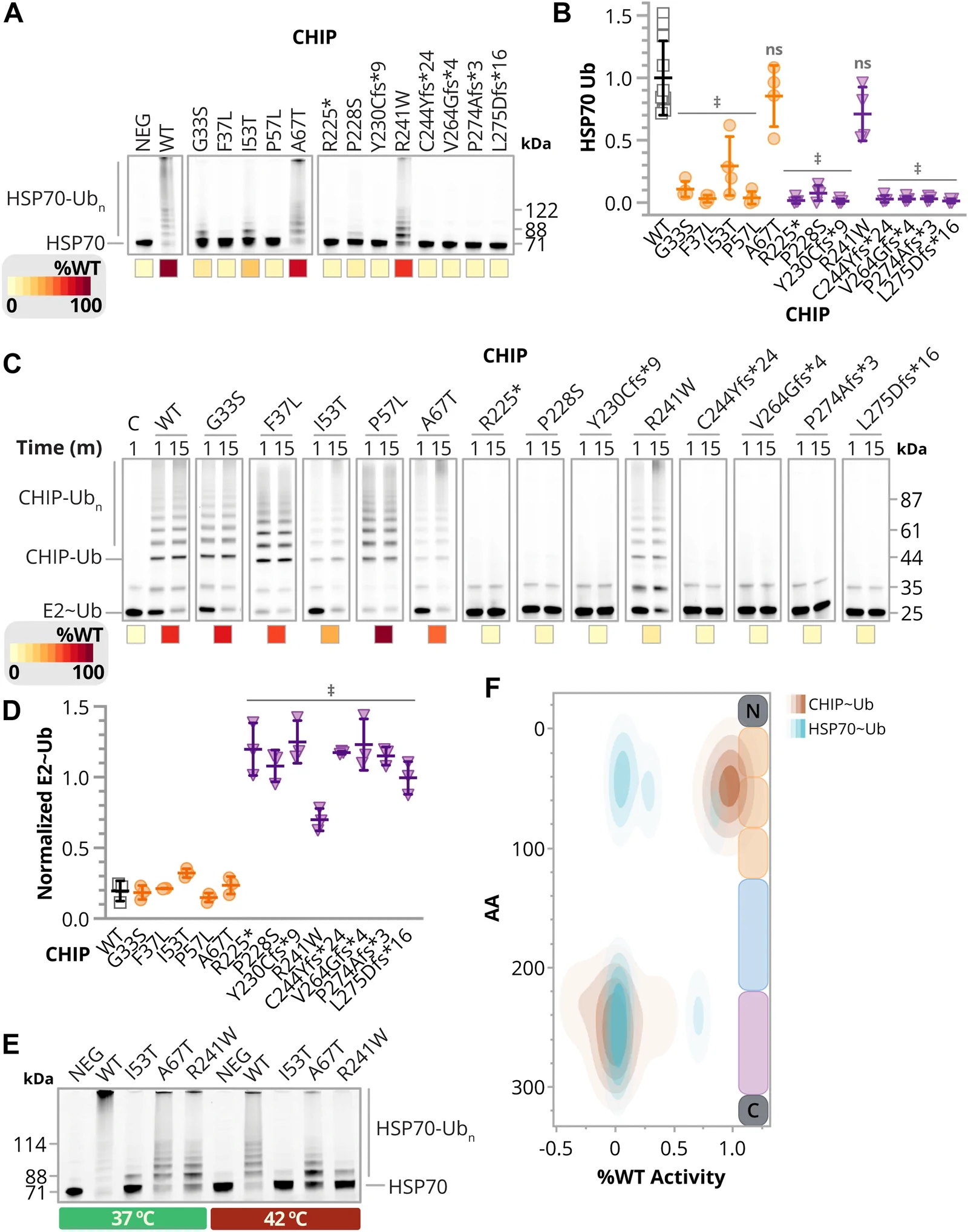
**Distinct effects of SCA48 mutations on CHIP-dependent ubiquitination activity.***A*, representative gel images of CHIP-dependent ubiquitination of fluorescently labeled HSP70, with increasing ubiquitination denoted by HSP70-Ub_n_ and summarized by a cell plot of total HSP70 ubiquitination relative to WT CHIP. *B*, densitometry analysis of HSP70 ubiquitination (HSP70 Ub) by each CHIP variant, represented by a dot plot and summarized by the mean ± SD (n = 4–11), was analyzed *via* one-way ANOVA (F(13, 49) = 26.7, *p* < 0.0001) and Dunnett’s multiple comparison test, ‡ and ns, indicate *p* < 0.0001 and > 0.05, respectively. *C*, representative gel images of CHIP autoubiquitination assay using fluorescently labeled ubiquitin (Ub) and summarized by a cell plot of autoubiquitination levels of each CHIP protein relative to WT CHIP. *D*, densitometry analysis of E2∼Ub levels from each condition (lower levels equate to higher autoubiquitination), represented by a dot plot and summarized by the mean ± SD (n = 3), was analyzed *via* one-way ANOVA (F(13, 28) = 67, *p* < 0.0001) and Dunnett’s multiple comparison test, ‡*p* < 0.0001. *E*, representative gel images of CHIP-dependent ubiquitination of fluorescently labeled HSP70, with increasing ubiquitination denoted by HSC70-Ub_n_ at 37 °C and 42 °C compared to (*C*). *F*, density heatmap of CHIP autoubiquitination (CHIP-Ub) and HSP70 ubiquitination relative to WT CHIP conditions and the amino acid position of the mutation.

Since its discovery, the ubiquitin (Ub) ligase activity of CHIP has been widely studied (8, 9, 27, 28, 29). CHIP facilitates the ubiquitination of molecular chaperones such as HSP70 and their bound substrates. Specifically, CHIP recruits co-chaperones and E2∼Ub through its TPR and U-box domains, respectively (9, 10). To assess the substrate ubiquitination activity of the SCA48 variants, we conducted CHIP-dependent substrate ubiquitination assays using fluorescently labeled HSP70 (Fig. 3, *A* and *B*). Overall, we observed reduced HSP70 ubiquitination across all SCA48 variants, except A67T and R241W.

CHIP’s substrate ubiquitination activity requires both a functional U-box domain for E2 recruitment and TPR domain for substrate recruitment. To separate these functions and assess the intrinsic Ub ligase activity of these CHIP variants, we conducted *in vitro* autoubiquitination assays using a fluorescently labeled Ub. As expected, mutations in the TPR domain largely did not impair intrinsic ligase activity, as shown by the increase in CHIP-Ub_n_ bands and the decrease in ubiquitin-conjugated E2 (E2∼Ub) bands (Fig. 3, *C* and *D*). In contrast, U-box domain mutations failed to exhibit autoubiquitination activity, except for some activity observed with R241W, suggesting an inactive U-box domain. (Fig. 3, *C* and *D*).

Three exceptions to this concept were I53T, A67T, and R241W. These enzymes showed a higher level of ubiquitination than the other mutants tested at 25 °C. However, CHIP activity is crucial during heat stress, and these mutations had among the lowest melting temperatures, suggesting that their ubiquitination activity might decline at higher temperatures. When their ubiquitination activity was tested at physiological temperature (37 °C) or heat-stress temperature (42 °C), CHIP-dependent HSP70 ubiquitination was notably reduced compared to wild-type CHIP, indicating that these variants would likely be defective in ubiquitination within cells (Fig. 3*E*). Taken together, these findings demonstrate that mutations in the TPR and U-box domains primarily reduce substrate ubiquitination activity through two mechanisms: failure to recruit substrates or inactivation of the U-box domain (Fig. 3*F*).

**Supporting Figure S4:**
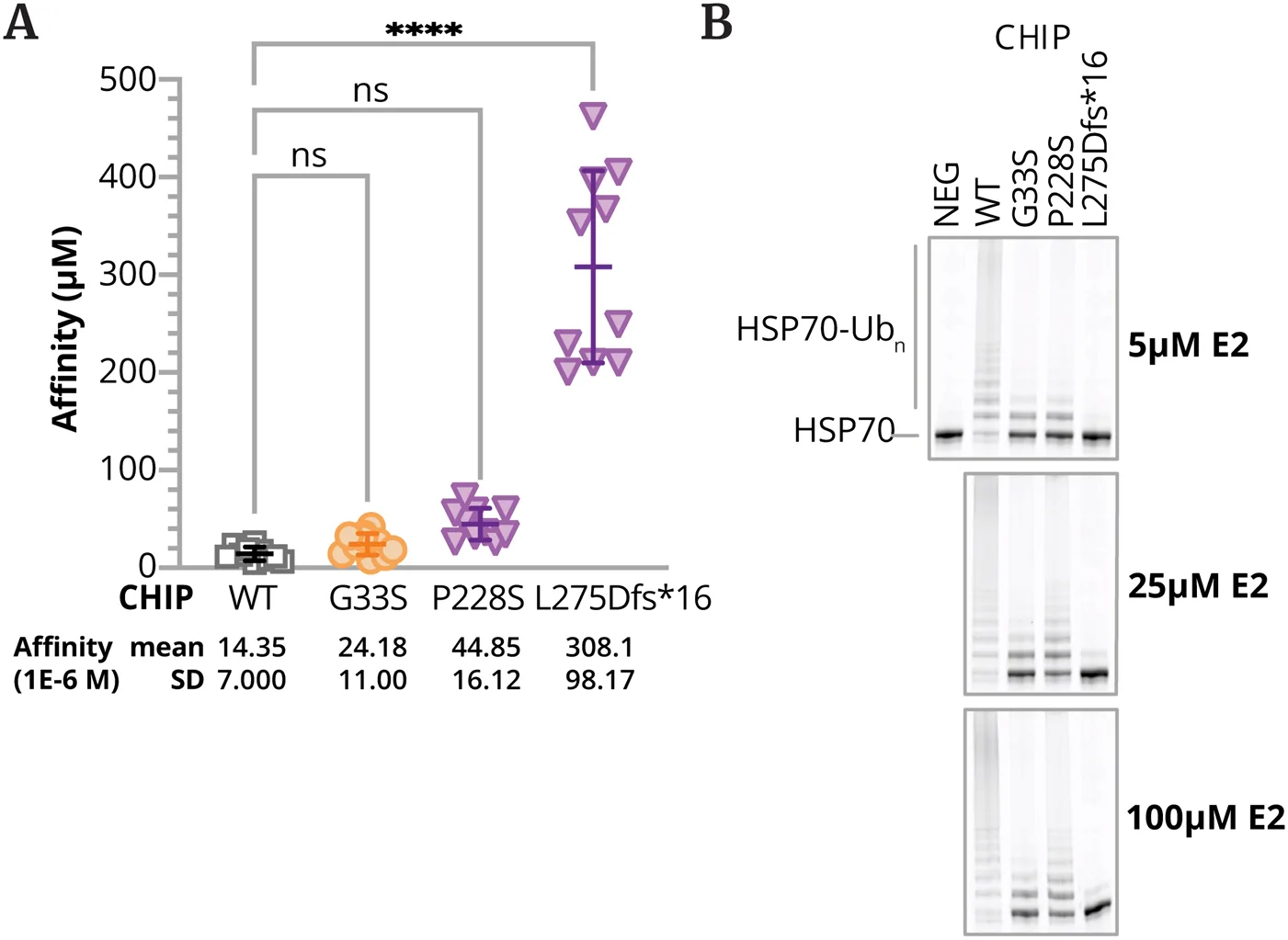


Next, we tested a set of CHIP variants (G33S, P228S, L275Dfs∗16) in their ability to bind and activate the E2 UBE2D2 using BLI and additional enzyme assays, respectively. As expected, the U-box variants exhibited weaker binding affinity for UBE2D2. When compared to WT, P228S was only mildly weaker in affinity (∼3-fold) for the E2 compared to L275Dfs∗16, which was ∼20-fold weaker (Fig. S4*A*). Consistently, we found that only the activity of the P228S variant could be partially rescued at the higher E2 concentration, but not to WT activity (Fig. S4*B*). Overall, these data suggest that improving the binding affinity between CHIP and the E2 may confer therapeutic benefit in some patients harboring U-box variants.

### SCA48 TPR mutations result in defective co-chaperone binding and activity

**Figure 4:**
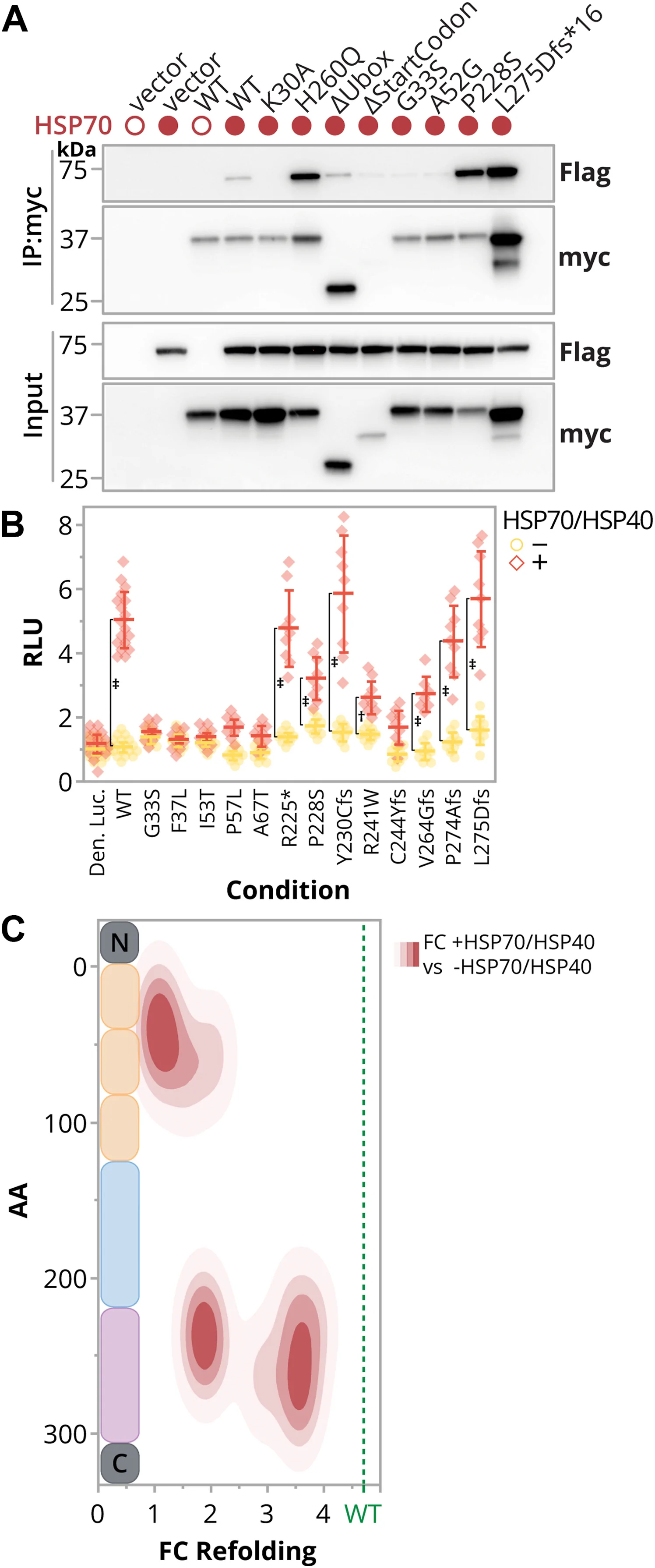
**SCA48 mutations differentially affect co-chaperone activity.***A*, Co-immunoprecipitation (IP) of CHIP and HSP70, along with input levels of both transgenes visualized by immunoblot, from COS-7 cells co-expressing the specified HSP70 and CHIP variants. *B*, co-chaperone refolding activity was assessed by measuring relative luciferase units (RLU) in heat-denatured luciferase refolding assays, with recombinant CHIP protein present (+) or absent (−) alongside HSC70 and HSP40. A two-way mixed model ANOVA of RLU (considering HSC70/HSP40 and CHIP), with replicate (n = 11) and day (n = 3) as random factors, resulted in F(14, 270) = 47.99, *p* < 0.0001 for the interaction term. Tukey post-tests indicated † and ‡, *p* < 0.01 and *p* < 0.0001, respectively, when comparing conditions with and without HSC70/HSP40. *C*, density heatmap of co-chaperone activity, summarized by fold change in luciferase refolding (FC refolding) in the presence of HSC70/HSP40 and the mutation’s amino acid position. Wild-type (WT) CHIP activity is indicated by the dashed line.

The observation that TPR substitutions lead to a loss of HSP70 ubiquitination but maintain its intrinsic ligase function suggests that these variants might also be impaired in their HSP70-dependent co-chaperone activity. Conversely, we hypothesized that U-box mutations would preserve their co-chaperone function, as observed with SCAR16 U-box mutations (19, 20). To begin assessing CHIP-HSP70 binding, we performed co-immunoprecipitation on lysates from COS-7 cells transfected with HSP70 and a select set of CHIP variants. Overall, less HSP70 co-precipitated with CHIP containing TPR mutations, including the previously identified SCA48 mutant A52G (21). In contrast, U-box mutants efficiently co-precipitated HSP70 (Fig. 4*A*). These results further indicate that the reduced ubiquitination of HSP70 in CHIP variants with TPR substitutions is due to a deficiency in substrate recruitment.

The interaction between HSP70 and the CHIP TPR domain is also essential for protein refolding and can be directly tested by measuring the refolding of heat-denatured luciferase in the presence of HSP70 and HSP40. While luciferase refolding required CHIP, HSP70, and HSP40, SCA48-associated substitutions in the TPR domain reduced luciferase refolding (Fig. 4, *B* and *C*) (17, 19). In contrast, the U-box variants showed different levels of luciferase refolding activity. Some frameshift variants and R225∗ maintained activity similar to CHIP WT, while P228S, R241W, C244Yfs∗24, and V264Gfs∗4 exhibited reduced co-chaperone activity (Fig. 4, *B* and *C*). Other than R241W, which is temperature sensitive, P228S, C244Yfs∗24, and V264Gfs∗4 were classified as forming non-discrete high molecular weight variants based on MP (Fig. S2*E*), suggesting that U-box mutations can impact more than CHIP’s established ubiquitin ligase activity.

### Domain-specific biochemical and structural divergence in SCA48 mutations

**Figure 5:**
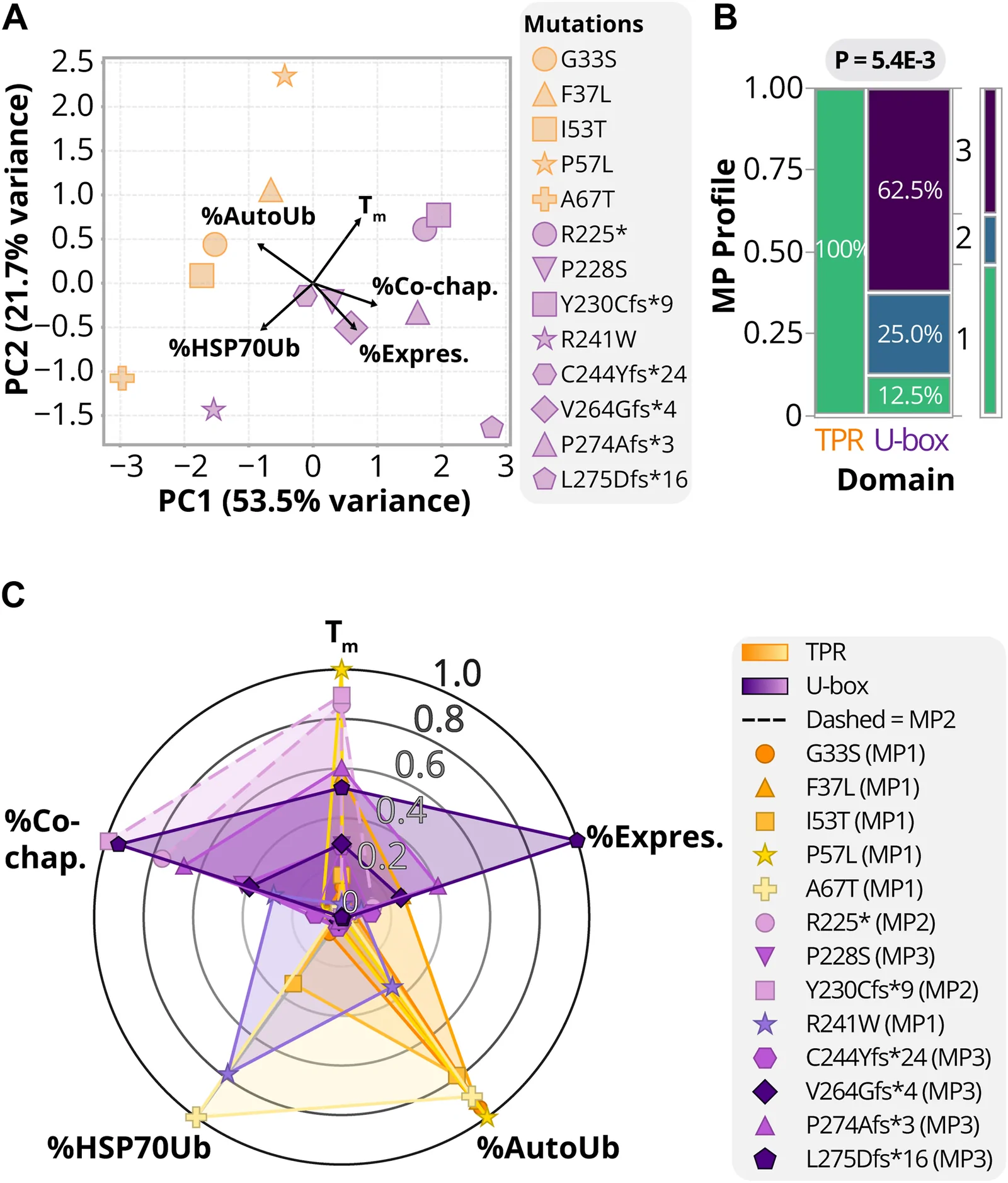
**Integrated biochemical and structural profiling reveals domain-dependent mechanisms of SCA48-associated CHIP dysfunction.***A*, principal component analysis (PCA) of normalized biochemical metrics: thermal stability (T_m_); steady-state expression (% Expres.); auto ubiquitination (%AutoUb); HSC70-dependent ubiquitination (%HSC70Ub); and co-chaperone activity (%Co-Chap), represented by a scatterplot. The first two principal components, PC1 (53.5%) and PC2 (21.7%), together explain 75% of the total variance, and domain-dependent separation on PC1 is confirmed *via t* test (t (11) = 3.3, *p* = 0.0069). *B*, contingency analysis of mass photometry (MP) profile distribution by domain reveals unequal distributions with *p* = 0.0054 *via* Fisher’s Exact Test. *C*, radar plot of the indicated biochemical quantitative metrics (normalized to WT CHIP = 1.0) for 13 *STUB1* mutations.

To characterize the biochemical and structural diversity of SCA48 mutations, we examined 13 mutations in the TPR and U-box domains of CHIP, combining multivariate, molecular, and domain-specific summaries (Fig. 5).

#### Principal component analysis uncovers domain-specific biochemical clustering

We performed a principal component analysis (PCA) on our normalized biochemical metrics, including thermal stability, expression levels, auto-ubiquitination, HSP70 ubiquitination, and co-chaperoning, to explore multidimensional relationships (Fig. 5*A* & Table S2). The first two principal components (PC1 and PC2) explained 75% of the total variance, with PC1 accounting for 53% and PC2 for 22%. The PCA revealed distinct clusters: TPR mutations (G33S, F37L, I53T, P57L) were mostly grouped in the quadrant associated with autoubiquitination loading, suggesting that autoubiquitination is a key feature of TPR mutations. A67T (TPR) and R241W (U-box) diverged, with %HSP70 ubiquitination as the primary driver, indicating a distinct ubiquitination profile for the substrate. U-box mutations R225∗ and Y230Cfs∗*9* are mainly affected by Tm, while the other U-box mutations (P228S, C244Yfs∗24, V264Gfs∗4, P274Afs∗3, and L275Dfs∗16) clustered with co-chaperone activity and expression loadings. A *t* test on PC1 scores confirmed significant separation between domains (t (11) = 3.3, *p* = 0.0069), supporting the biochemical distinction between TPR and U-box mutations.

#### Mass photometry identifies domain-specific molecular assemblies

Structural insights from mass photometry (MP) profiling highlighted significant domain-dependent differences in molecular assembly (Fig. 5*B*). All TPR mutations (G33S, F37L, I53T, P57L, A67T) and the temperature sensitive Ubox mutant (R241W) exhibited an MP profile 1, characterized by dimer repeat units, whereas U-box mutations were predominantly MP profile 3, indicative of high non-discrete high-molecular-weight oligomers (P228S, *C244Yfs*24, V264Gfs*4, P274Afs*3, L275Dfs∗16), with and R225∗ and Y230Cfs*9* as MP profile 2 (dimer only). A contingency analysis confirmed this distribution (*p* = 5.4 × 10^−3^), suggesting that oligomerization propensity may differentiate TPR and U-box pathobiology, potentially influencing cellular localization and function. MP measurements were at a final concentration of 50 nM, a physiological range for chaperone studies, but CHIP oligomerization is inherently concentration-dependent, so these profiles may shift at higher intracellular levels or at lower concentrations.

#### Integrating domain-specific biochemical signatures

A radar plot visualized the normalized biochemical metrics across all 13 mutations, highlighting domain-specific patterns (Fig. 5*C* & Table S2). TPR mutations maintained intact U-box function, consistent with their PCA clustering and MP Profile 1 dimerization. Conversely, U-box mutations showed increased steady-state expression levels and co-chaperone activity, aligning with their oligomer-prone MP Profile 3 and PCA distribution. Overall, thermal stability and steady-state expression levels exhibited the greatest mutation-specific variability within each domain, providing a comprehensive overview linking structural and functional data to downstream cellular and clinical outcomes.

### Mechanistic insights into CHIP dysfunction in SCA48

The radar plot (Fig. 5*C* & Table S2) summarizes our biochemical profiling and reveals distinct domain-specific signatures among SCA48 mutations in the *STUB1*/CHIP protein: TPR variants, such as G33S, maintain ubiquitination activity but have reduced chaperone function and expression, while U-box variants, including P228S and L275Dfs16, show preserved chaperone interactions but nearly no ligase activity. These contrasting profiles suggest a decoupling of substrate recognition and degradation, which may contribute to proteostatic imbalance in affected cells.

**Figure 6:**
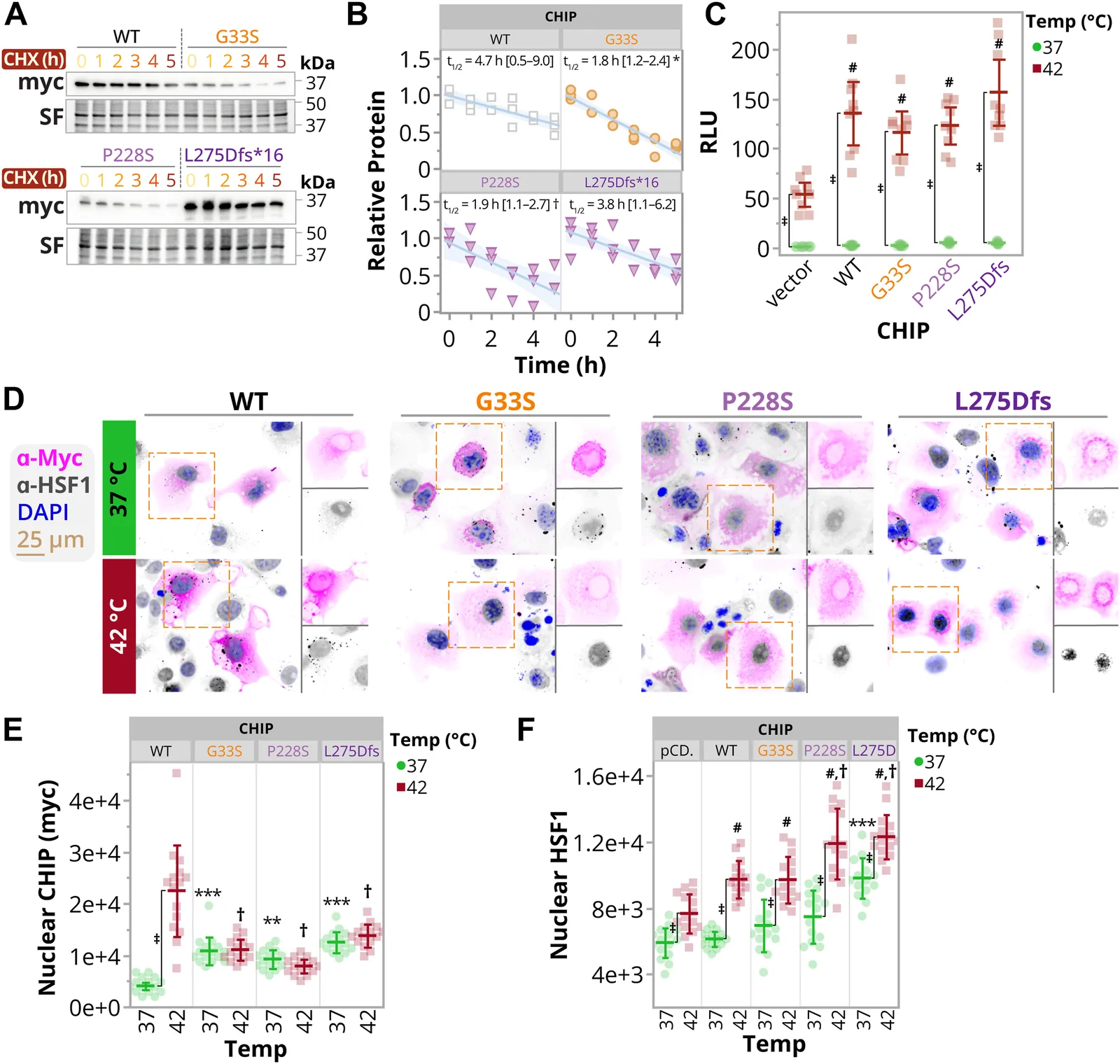
**Impact of SCA48 mutations on CHIP stability and heat shock response regulation.***A*, representative immunoblot of cycloheximide (CHX) chase detecting myc-CHIP with stain-free (SF) loading. *B*, densitometry scatterplot of relative CHIP across time (n = 3 biological replicates, technical duplicates). Half-lives (inline) estimated by mixed-design log-linear regression of ln(intensity) on time with biological replicate as fixed-effect block, shown as point estimate [95% CI]. Time × variant interaction terms with Holm–Bonferroni correction: ∗ Holm-adj *p* < 0.05; † Holm-adj *p* < 0.10. *C*, HSF1-driven luciferase reporter activity (mean ± SD, n = 9 = 3 biological replicates × 3 assay days). Two-way mixed ANOVA (Heat Shock × CHIP condition; replicate and day as random factors): F(1, 78) = 545.6; F(4, 78) = 13.3; F(4, 78) = 11.7; all *p* < 0.0001. Tukey: ‡*p* < 0.0001 within-CHIP (42 *versus* 37 °C); #*p* < 0.0001 *versus* vector at 42 °C. *D*, representative immunofluorescence of COS-7 cells expressing WT or the indicated mutant CHIP-myc (*magenta*) with HSF1 (*gray*) and DAPI (*blue*) at 37 °C and 42 °C; *dashed boxes* mark single-channel insets (pcDNA vector control, Fig. S5). *EandF*, per-cell nuclear CHIP (*E*) and nuclear HSF1 (*F*) (mean ± SD, n = 30 cells/condition); two-way ANOVA (temperature × CHIP variant). Nuclear CHIP: F(1, 140) = 49.57; F(4, 140) = 85.93; F(4, 140) = 49.21; all *p* < 0.0001. Tukey: ‡*p* < 0.0001 (WT only, 42 *versus* 37 °C); ∗∗*p* < 0.001, ∗∗∗*p* < 0.0001 (mutant *versus* WT at 37 °C); †*p* < 0.0001 (mutant *versus* WT at 42 °C). Nuclear HSF1: F(1, 140) = 182.64, F(4, 140) = 43.94 (both *p* < 0.0001); interaction F(4, 140) = 4.24, *p* = 0.0028. Tukey: ‡*p* < 0.0001 within-CHIP (42 *versus* 37 °C); ∗∗∗*p* < 0.0001 (L275Dfs∗16 *versus* WT at 37 °C); #*p* < 0.0001 (P228S, L275Dfs∗16 *versus* vector at 42 °C); †*p* < 0.001 (P228S, L275Dfs∗16 *versus* WT at 42 °C).

**Figure 7:**
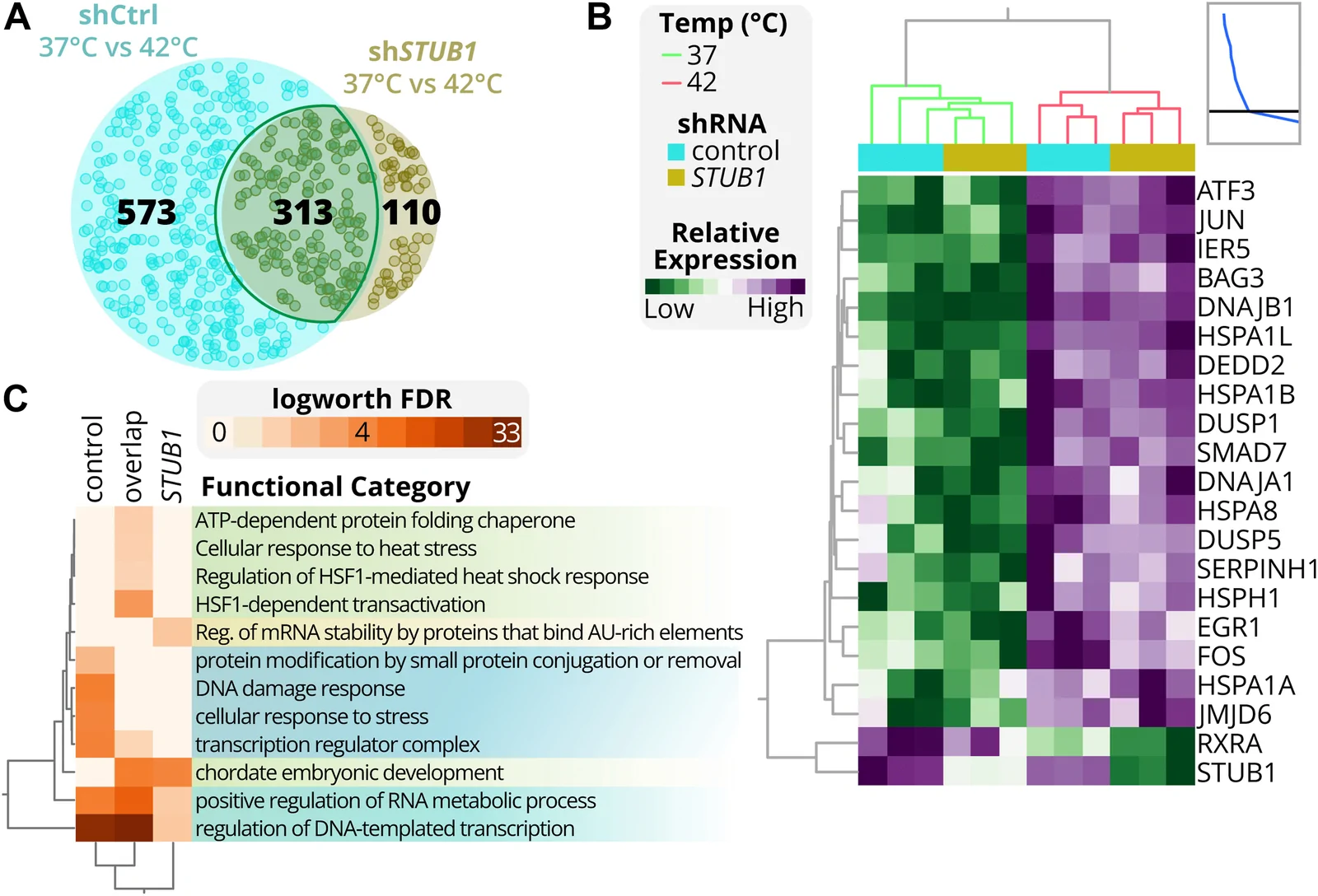
**CHIP modulates HSF1-driven transcriptional responses and stress-induced gene networks.***A*, RNA-sequencing was performed on shCtrl or sh*STUB1* HEK-293 cells under basal conditions and following heat shock. Heat stress-induced differential gene expression (DGE, defined as corrected *p* < 0.05) identified 886 and 423 genes in shControl and sh*STUB1* cells, respectively; 313 genes overlapped, as summarized in a proportional Venn diagram. *B*, a heatmap of known critical HSF1 target genes (within the 313-gene overlap) showed largely preserved HSF1-mediated transcriptional activation, including one known heat-stress-suppressed target (RXRA), and confirmed *STUB1* knockdown in the RNAseq data. *C*, selected functional enrichment categories of the DEGs from the three Venn diagram regions (in *A*), represented by a hierarchical clustered heatmap of the logworth false discovery rate (FDR).

To test whether our domain-specific biochemical signatures predict cellular dysfunction, we selected three prototypic mutants for detailed cell biology: G33S (TPR missense, MP Profile 1, low expression/chaperone activity), P228S (U-box missense, MP Profile 3, high expression/HMW), and L275Dfs16 (U-box frameshift, MP Profile 3, confirming truncating mutations cluster with U-box missense defects; Fig. 5), focusing on protein stability, post-translational modifications, stress responses, subcellular localization, and transcriptional regulation (Figs. 6 and 7). Notably, R241W (U-box) and A67T (TPR) emerged as partial function outliers in biochemical profiling: R241W retained dimerization and partial ligase activity, while A67T showed preserved substrate ubiquitination despite its TPR location (Fig. 5). Both exhibit temperature-sensitive defects (Fig. 3*E*), suggesting these variants may represent intermediate phenotypes within the domain-specific framework.

#### TPR-domain mutations accelerate CHIP turnover

Cycloheximide chase experiments assessed whether the domain-specific defects extend to protein stability (Fig. 6*A*). The TPR variant G33S decayed significantly faster than WT (variant × time interaction, Holm-adjusted *p* = 0.038; t½ = 1.8 h *versus* 4.7 h for WT). The U-box missense P228S showed a similar but non-significant trend (*p* = 0.068; t½ = 1.9 h), and the U-box frameshift L275Dfs∗16 was indistinguishable from WT (*p* = 0.65; t½ = 3.8 h) (Fig. 6*B*). Because the derived half-lives have wide, substantially overlapping confidence intervals, our conclusions about destabilization rest on the variant × time interaction terms rather than on pairwise half-life comparisons; on that basis, only G33S is significantly destabilized. These data are consistent with TPR-domain mutations destabilizing CHIP by reducing co-chaperone engagement, whereas U-box frameshifts leave turnover largely intact.

#### Stabilizing phosphorylation of CHIP does not explain differences in half-life

**Supporting Figure S5:**
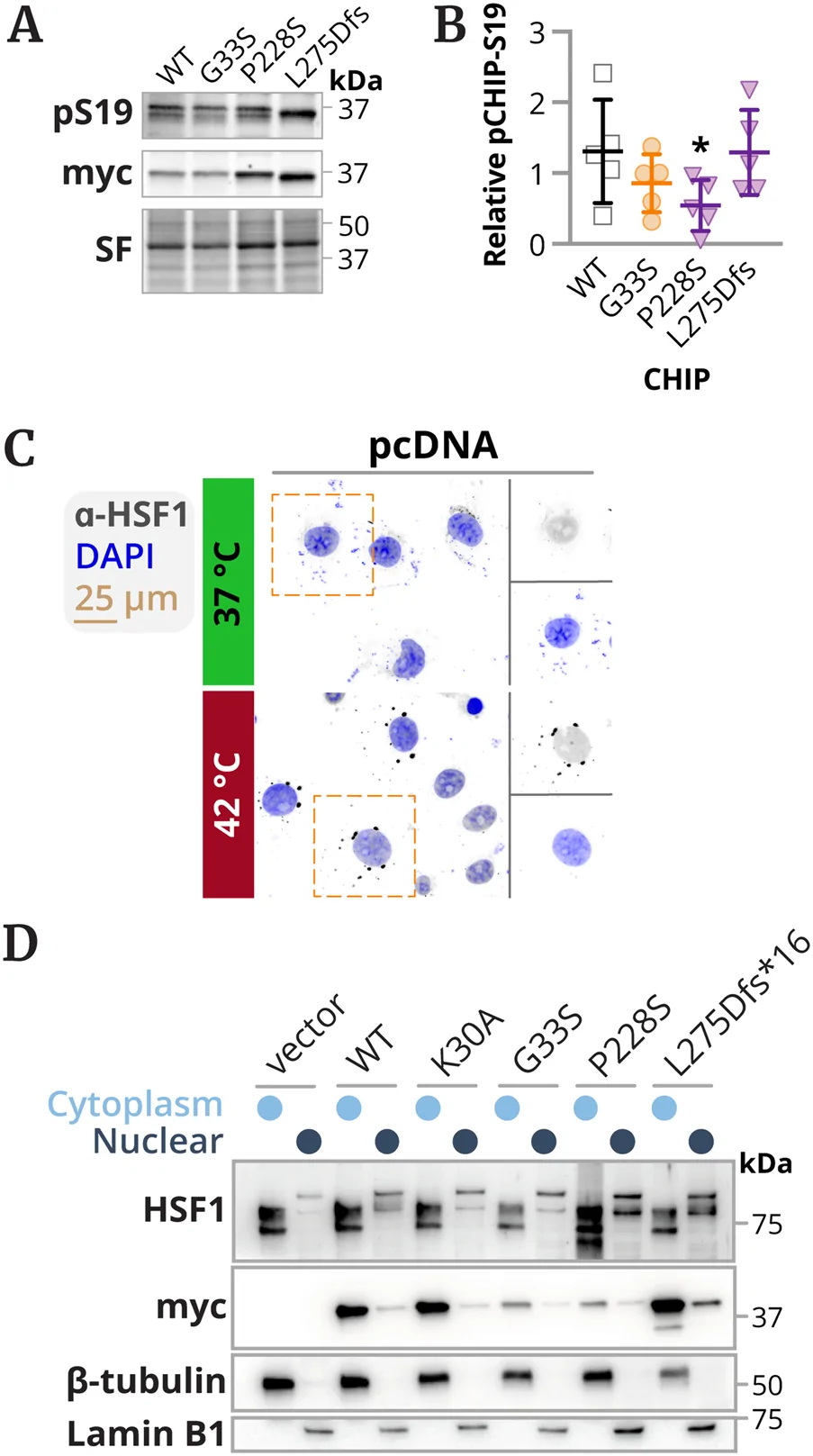


Given the role of post-translational modifications in regulating CHIP stability, we examined phosphorylation at serine 19 (pS19), a modification previously linked to increased stability (30). Western blot analysis showed that P228S had lower pS19 levels than WT, whereas G33S and L275Dfs∗16 maintained WT-like levels (Fig. S5, *A* and *B*). This decrease in P228S suggests a possible reduction in pS19-mediated stability; however, its half-life was similar to WT, indicating that pS19 levels alone do not determine steady-state abundance. Conversely, the significant decrease in half-life observed in G33S (Fig. 6, *A* and *B*), despite unchanged pS19, suggests other regulatory mechanisms, possibly related to impairment of its TPR domain, that accelerate degradation. Overall, these results emphasize a complex relationship between mutation-driven structural changes and post-translational regulation in determining CHIP stability across SCA48 variants.

#### SCA48 mutations cause CHIP mislocalization

An important role of CHIP is to regulate the heat shock response by promoting the trimerization and nuclear translocation of heat shock factor 1 (HSF1), thereby activating heat shock response genes (31). To assess heat shock responses mediated by CHIP and HSF1, we used a dual-luciferase reporter assay with a plasmid expressing luciferase driven by heat shock elements. Overexpressing WT CHIP increased heat-induced HSF1 transcriptional activity (Fig. 6*C*), as heat facilitated the nuclear movement of both HSF1 and CHIP (Figs. 6, *D*–*F* and S5*C*). Surprisingly, the selected SCA48 mutants boosted the heat shock transcriptional response similarly to WT (Fig. 6*C*), which matched increases in heat-induced HSF1 nuclear translocation (Fig. 6, *D* and *F*), indicating this process might be independent of HSP interactions. However, heat shock-induced nuclear translocation of CHIP was missing in all mutants (Fig. 6, *D* and *E*). Notably, the normal localization of CHIP at 37 °C was changed, with all mutants showing higher nuclear presence compared to WT CHIP, implying misregulated nucleocytoplasmic shuttling or retention as a potential feature of SCA48 (Fig. 6, *D* and *E*). Fractionated immunoblotting and immunofluorescence further supported these results: WT CHIP overexpression increased nuclear localization of both CHIP and HSF1 during heat shock (Figs. 6, *D*–*F* and S5*D*). The G33S mutant reduced CHIP nuclear localization with a slight defect in HSF1, while P228S and L275Dfs16, despite decreased CHIP localization, maintained or modestly increased HSF1 nuclear levels compared to WT (Figs. 6, *D*–*F* & S5*D*). This rise in HSF1 activity in U-box mutants may reflect an altered or dysregulated stress response with potential toxic consequences, rather than a simple loss-of-function mechanism. Overall, these findings suggest that CHIP-dependent HSF1 nuclear localization does not strictly depend on HSP interaction but relies on CHIP’s own nuclear import.

#### CHIP insufficiency amplifies the transcriptional heat shock response beyond canonical HSF1 targets

**Supporting Figure S6:**
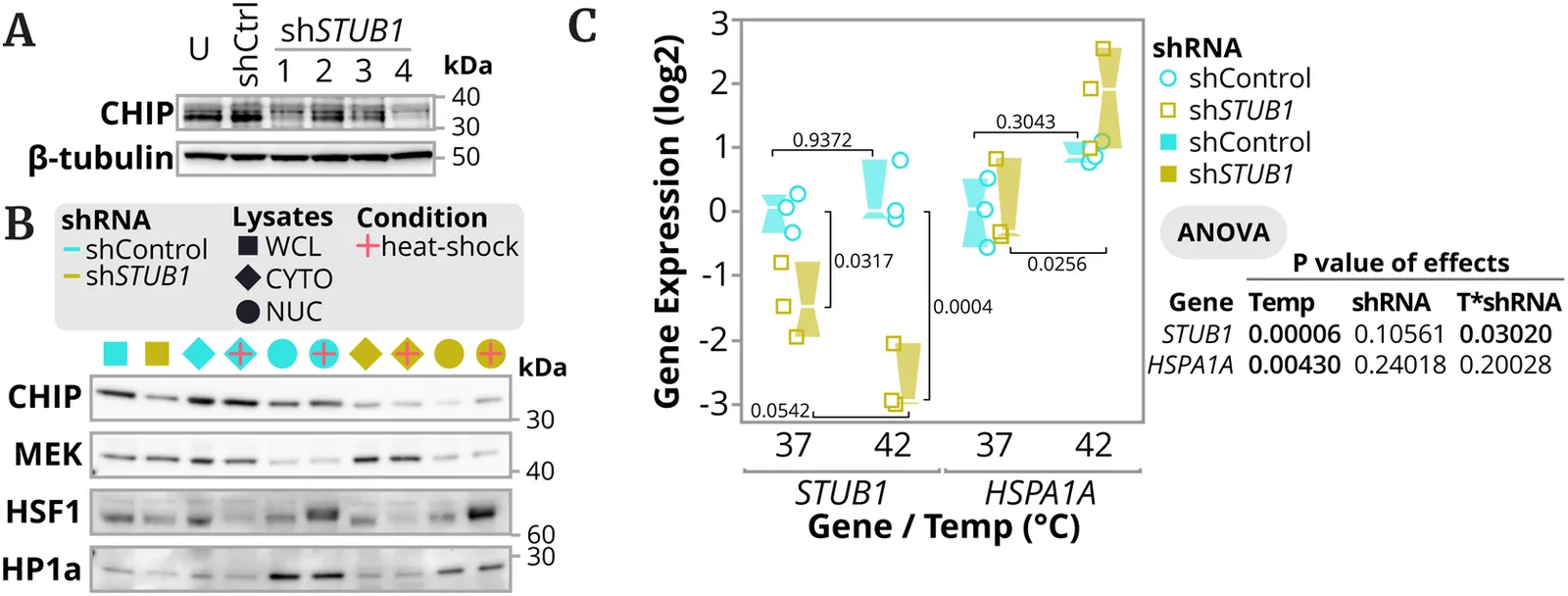


To explore CHIP-dependent transcriptional changes in a model of CHIP insufficiency, we performed RNA-Seq on HEK-293 cells transduced with lentiviruses expressing shRNAs (shControl and sh*STUB1*) under both normal and heat-shock conditions (Figs. 7*A* and S6, *A*, *B*). We observed transcriptional changes in 886 genes in shControl cells and 423 genes in sh*STUB1* cells after heat shock, with 313 genes overlapping. These results suggest significant overlap in heat-stress responses even in the presence of CHIP knockdown. Next, we identified HSF1 transcriptional targets from the 313 DEG overlap, revealing remarkably conserved responses between shControl and sh*STUB1* cells after heat shock, with all HSF1-driven changes maintained (Fig. 7*B*), consistent with nuclear HSF1 levels (Fig. S6*B*). Functional enrichment analysis of the DEGs further revealed that the overlapping DEG set was enriched for HSF-mediated transcription (Figs. 7*C* and S6*C*). Nonetheless, shControl cells exhibited five times as many transcriptional changes as sh*STUB1*, with unique enrichments in ATP-dependent protein folding chaperone activity, protein modification by small conjugation or removal, regulation of DNA-templated transcription, positive regulation of RNA metabolic processes, and cellular stress responses, including DNA damage repair. The loss of these categories in sh*STUB1* cells suggests reduced chaperone and ubiquitination activities, consistent with proteostatic defects observed in SCA48, and implies that CHIP enhances the transcriptional heat shock response through mechanisms beyond HSF1, potentially involving nuclear nodes where CHIP modulates DNA damage repair and broader stress pathways.

### Domain-specific correlations with clinical phenotypes in SCA48 patients

**Figure 8:**
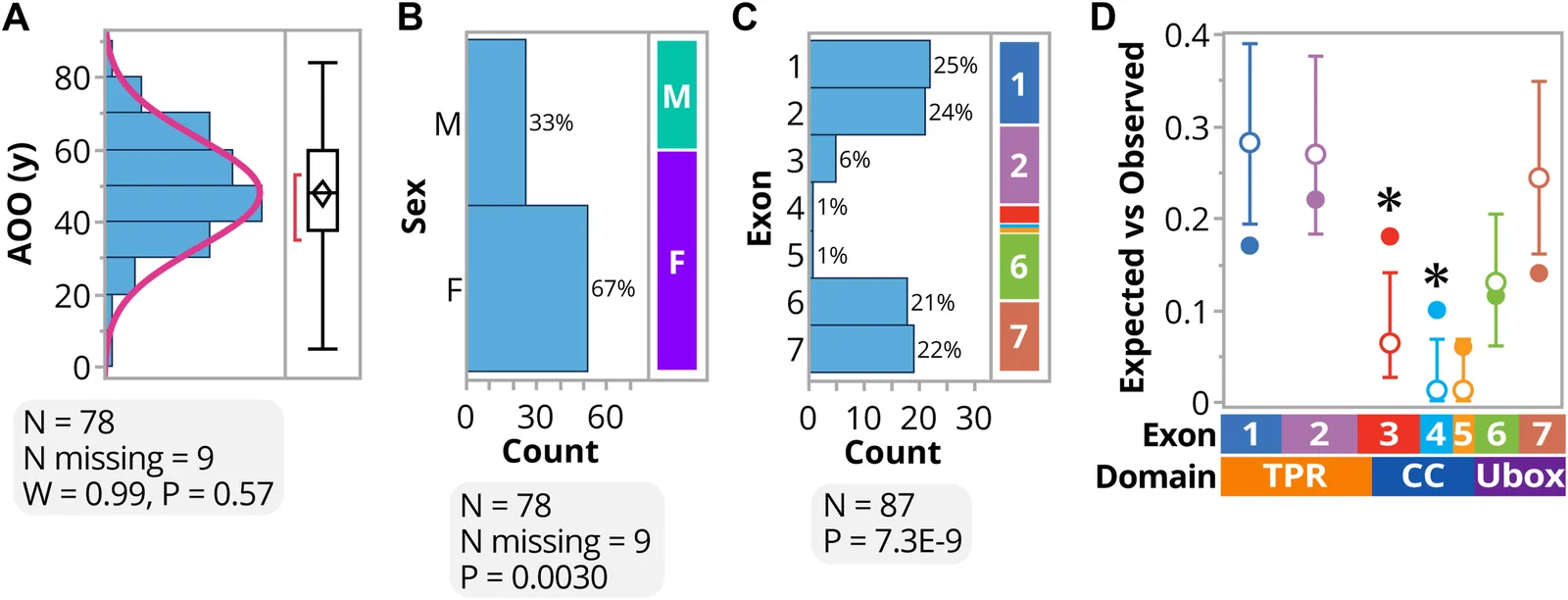
**Domain-specific distribution of SCA48 mutations and demographic patterns.***A*, Reported age of onset (AOO) histogram and box-whisker plot, with overlaid Shapiro-Wilk normality test. *B*, sex frequency; chi-squared goodness-of-fit *versus* 1:1 ratio. *C*, distribution of mutations per exon; chi-squared goodness-of-fit *versus* expected exon frequencies. *D*, expected (○, mean ± 95% CI) *versus* observed (○) mutation frequency per exon; ∗ BH-FDR *p* < 0.05.

Building on the domain-specific biochemical and structural differences observed in SCA48 mutations, we hypothesized that these molecular signatures might be associated with clinical phenotypes. To test this, we conducted a meta-analysis of 87 SCA48 patients from published case studies, gathering data on demographics (age of onset [AOO], sex), mutation locations, Scale for the Assessment and Rating of Ataxia (SARA) scores, and five categorical clinical features representing core symptoms (Table S3) (4, 22, 32, 33, 34, 35, 36). The analysis revealed a median AOO of 48 years, with a female majority (about two-thirds of patients; Fig. 8, *A* and *B*). Mutations mainly occurred in exons 1 to 2 (which encode the TPR domain) and 6 to 7 (which encode the U-box domain), while only 8% were found in exons encoding the coiled-coil (CC) domain (Fig. 8*C*). To determine if this distribution was random, we calculated expected mutation frequencies weighted by exon length. Mutations were more frequent than expected in exons 1 (TPR, observed/expected ratio = 1.6, unadjusted/Holm-adjusted *p* = 0.0168/0.0673) and 7 (U- box, ratio = 1.8, *p* = 0.0116/0.0579), and less common in exons 3 and 4 (CC, ratio = 0.35 and 0.13; *p* = 0.0048/0.0335 and 0.0063/0.0376, Fig. 8*D*). These differences, tested by chi-square goodness-of-fit while controlling for exon length, suggest exon-specific intolerance: TPR and U-box exons may harbor mutations with higher pathogenicity or be influenced by detection bias, while CC exons face strong selective pressure, possibly due to embryonic lethality or non-viable phenotypes in homozygous models like *Stub1* knockout mice (3). Compared to SCAR16 (recessive CHIP mutations), where CC domain variants are more common and linked to cognitive deficits and hypogonadism (37), the rarity of CC variants in SCA48 is consistent with a model in which many SCA48 mutations act through dominant mechanisms. Disruptive CC variants may completely prevent CHIP dimerization, resulting in nonviable or distinct phenotypes. Limitations include potential publication bias favoring symptomatic mutations and limited sequencing depth in CC regions across studies, highlighting the need for larger, uniformly analyzed cohorts.

**Figure 9:**
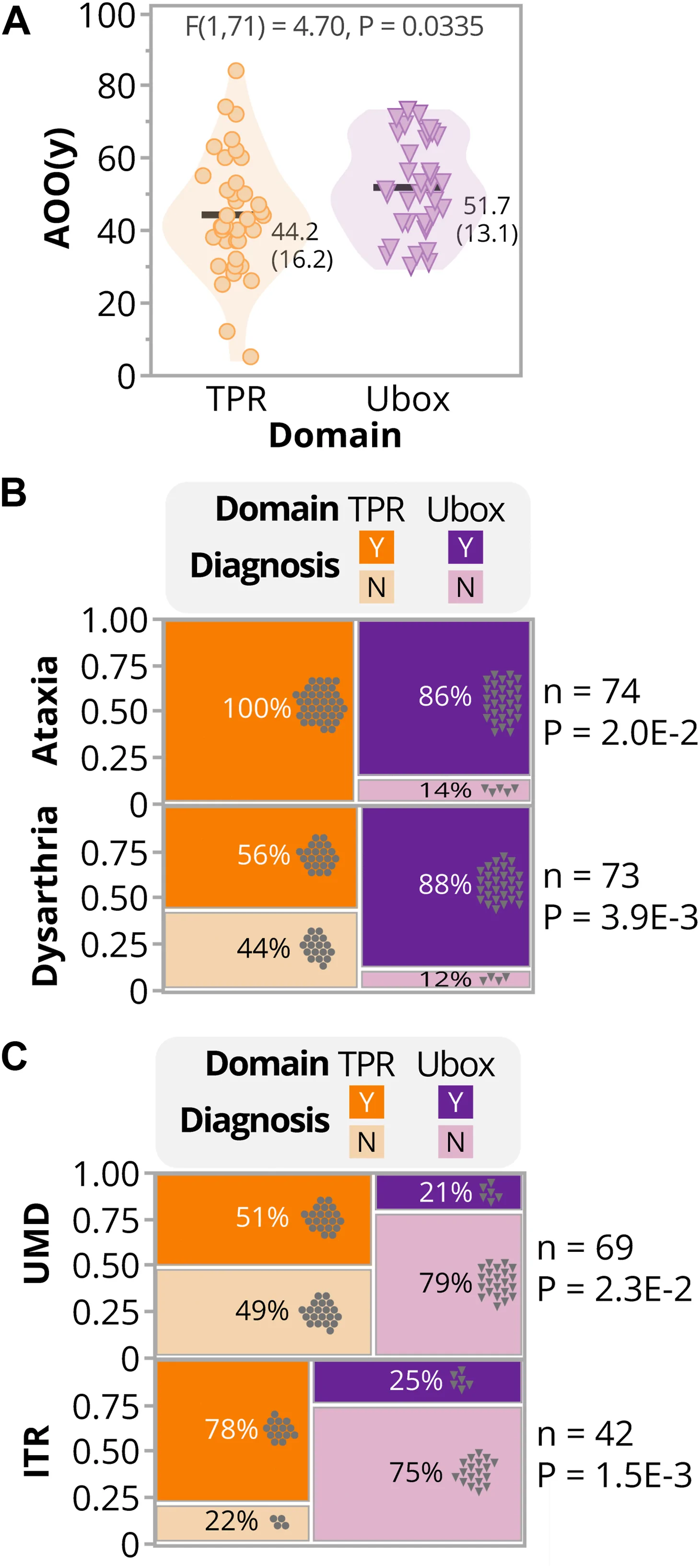
**Genotype-phenotype correlations reveal domain-dependent clinical features in SCA48.***A*, age of onset (AOO) *versus* the domain harboring the mutation represented by a violin dot plot, summarized by the mean and standard deviation, analyzed *via* a two-tailed *t* test. *B & C*, chi-squared analysis of gait ataxia, dysarthria, upper motor-neuron dysfunction (UMD), and increased tendon reflexes (ITR) represented by a contingency plot. The raw *p* values of two-tailed Fisher’s exact test are reported, and all fall under the 5% FDR correction for multiple tests.

**Supporting Figure S7:**
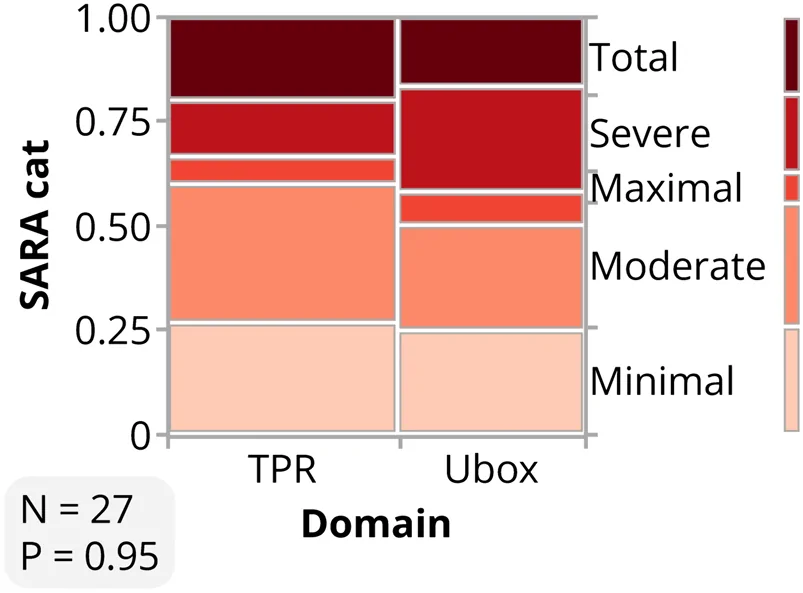


We then analyzed the relationships between mutation location and clinical phenotypes. U-box mutations were associated with a later age of onset (AOO), with a median of 51 years compared with 42 years for TPR mutations (*p* = 0.0321; Fig. 9*A*), possibly indicating milder early proteostatic effects. However, there was no significant difference in SARA scores between TPR and U-box groups (median 12 *versus* 13, *p* = 0.9490, N = 27; Fig. S7). This suggests that overall disease severity is not solely determined by mutation domain, although factors such as sample size, reporting bias, and variability in assessment timing also influence results. Contingency analysis of categorical symptoms, adjusted for multiple comparisons using false discovery rate (FDR), showed no domain-specific differences in cognitive dysfunction or nystagmus (FDR-adjusted *p* = 1.0 or 0.6356, respectively; Table S3). Nevertheless, ataxia was present in 100% of TPR mutations (*versus* 86% in U-box; FDR-adjusted *p* = 0.0344; Fig. 9*B*). Dysarthria exhibited a skewed distribution favoring U-box mutations (88% *versus* 56% in TPR; FDR-adjusted *p* = 0.0117; Fig. 9*B*). Conversely, TPR mutations were associated with upper motor neuron dysfunction, including increased tendon reflexes (ITR; 78% *versus* 25%, FDR-adjusted *p* = 0.0090; Fig. 9*C*) and signs of upper motor neuron involvement (51% *versus* 21%, FDR-adjusted *p* = 0.0344; Fig. 9*C*). These FDR-corrected associations (q < 0.05) support the idea that mutation location influences the heterogeneity of the SCA48 spectrum, with TPR mutations predisposing to motor neuron deficits and U-box mutations tending toward dysarthria-dominant presentations. However, limitations include small sample sizes for some features (*e.g.*, ITR N = 42), potential variability in clinical reporting across studies, and ascertainment bias toward more severe cases, which restricts broader applicability. Future research with larger, prospective cohorts and standardized phenotyping is needed to validate these findings.

## Discussion

Our study provides a detailed biochemical and structural framework for understanding how SCA48-associated *STUB1* mutations disrupt CHIP function at multiple levels, including protein stability (Figs. 1 and 6), post-translational regulation (Fig. 6), oligomeric assembly (Fig. 2), and stress response dynamics (Figs. 4, 6 and 7). These combined findings enhance the mechanistic understanding of how domain-specific mutations interfere with proteostasis pathways and contribute to cellular degeneration. The results emphasize the domain-specific effects of SCA48-related mutations in *STUB1*/CHIP, revealing different mechanisms of dysfunction in the TPR and U-box domains (Fig. 5). TPR mutations, such as G33S, F37L, I53T, P57L, and A67T, primarily impair substrate recruitment and co-chaperone activity while maintaining essential ubiquitin ligase function, as evidenced by decreased HSP70 ubiquitination and luciferase refolding efficiency (Figs. 3 and 4). Conversely, U-box mutations, including frameshifts and nonsense mutations such as R225∗, P228S, and L275Dfs∗16, eliminate ligase activity but often preserve or enhance co-chaperone interactions, resulting in increased protein levels and altered oligomerization (Fig. 5). These observations align with previous reports on *STUB1* mutations in related ataxias, such as SCAR16, in which U-box disruptions similarly impair ubiquitination without completely disrupting chaperone binding (19, 21). The temperature sensitivity observed in variants such as I53T, A67T, and R241W further suggests that environmental stressors, similar to those that induce mRNA CHIP expression in cells (38), could worsen functional deficits *in vivo* and may contribute to the progression of neurodegeneration in SCA48 (39). On the other hand, although ubiquitination defects generally stabilize CHIP by reducing autoubiquitination and proteasomal degradation (22, 23), our CHX chase experiments showed that this is not always the case (Fig. 6). The loss of co-chaperone activity and substrate interaction, often seen in TPR mutants such as G33S, results in a significantly shorter half-life, suggesting that other factors, such as protein misfolding or changes in structural dynamics, may accelerate turnover. The shorter half-life of G33S and the altered phosphorylation at S19 in P228S U-box mutants suggest post-translational modifications, such as those mediated by protein kinase G (30), may help maintain stability. Consistent with this, variants with lower thermal stability (*e.g.*, G33S, I53T, A67T, and R241W) also exhibit predicted steric clashes in the CHIP dimer interface (Fig. S1), which likely destabilize the overall protein structure and decrease steady-state levels (21, 24). These findings imply that destabilizing mutations disrupt the balance between CHIP formation and degradation, leading to variable protein levels across SCA48 variants.

Biophysical analyses, including differential scanning fluorimetry (Table 1) and mass photometry (Fig. 2), show that SCA48 mutations cause distinct changes in protein stability and oligomerization, which likely account for the observed functional differences (Fig. 5). TPR mutants typically form distinct dimer-repeating oligomers (MP Profile 1), allowing some residual activity, whereas most U-box mutants tend to oligomerize into non-discrete high-molecular-weight species (MP Profile 3), which correlates with reduced heat-induced oligomer shifts in cellular assays. This tendency to form higher-order oligomers is consistent with studies on CHIP's role in protein quality control, in which oligomerization influences chaperone-dependent ubiquitination and refolding. Dimerization is critical for co-chaperone binding and ubiquitination, whereas higher-order oligomers are required for robust chaperone activity under cellular stress. Mass photometry and biochemical profiling indicate that TPR-domain mutations largely preserve dimer formation, whereas U-box variants often yield non-discrete higher-order species that may be aggregates (Fig. 2). This change in oligomerization is not merely structural; it likely causes downstream effects, including impaired substrate processing and abnormal stress responses. Notably, mutant CHIP’s failure to form higher-order oligomers during heat shock was linked to impaired nuclear translocation and an altered heat shock response, highlighting the importance of oligomeric regulation. These biochemical defects impact the regulation of HSF1, a key player in the cellular stress response. CHIP promotes HSF1 trimerization and nuclear translocation, thereby boosting the transcription of protective heat shock genes. Although SCA48 mutants maintained HSF1 activity under stress, they couldn’t transfer CHIP to the nucleus and, interestingly, showed increased basal nuclear localization (Fig. 6). This misregulation suggests that CHIP’s movement between the nucleus and cytoplasm is sensitive to structural changes, and abnormal nuclear retention or mislocalization might be a hallmark of SCA48 pathology. Additionally, some U-box mutants, such as L275Dfs∗16, still enhance nuclear accumulation of HSF1, suggesting potential gain-of-function effects that could dysregulate the stress response and promote disease progression. The sh*STUB1* design models CHIP insufficiency; domain-specific RNA-seq profiling of TPR and U-box mutants would directly test whether the biochemical decoupling we observe translates to distinct transcriptional programs.

Clinical correlations from the meta-analysis of 87 SCA48 patients show that mutation location affects phenotypic presentation: U-box mutations are associated with later age of onset, and TPR mutations are associated with upper motor neuron signs, including increased tendon reflexes (Fig. 9). Biochemical profiles, such as low expression, increased autoubiquitination, and decreased co-chaperone activity in TPR variants, support these skewed patterns (Fig. 5), suggesting that proteostatic imbalances contribute to symptoms such as ataxia and dysarthria (Fig. 9). This domain-specific pattern resembles genetic modifiers observed in other SCAs, in which *STUB1* variants interact with repeat expansions in genes such as *ATXN8OS*/*ATXN8* or *TBP* to influence penetrance and severity (5, 40). The concentration of mutations in exons encoding TPR and U-box domains, which differs from a random distribution (Fig. 8), indicates selective pressure on functional hotspots, consistent with CHIP's dual role in proteostasis and neurodegeneration models (41). While our data offers valuable insights into CHIP domain-specific functions and their impact on SCA48 clinical features, further research is necessary to understand how other genetic factors, such as intermediate TBP41-49, operate within the SCA48 context and influence the disease phenotype. A recent study of patients with spinocerebellar ataxia 17 (SCA17) and intermediate glutamine repeat expansions (41–49 repeats) in the TATA-box-binding protein (TBP) gene (TBP41-49) identified heterozygous mutations in *STUB1* (6). Interestingly, while *TBP* alleles with over 49 glutamine repeats are fully penetrant and considered pathological, the TBP41-49 allele shows incomplete penetrance; its frequency in the general population is about 2%, and approximately 50% of carriers of intermediate alleles remain healthy (6). This raised questions about whether *STUB1* mutations are causal or merely disease-modifying. A follow-up study identified normal *TBP* alleles (<40 repeats) in several families with different *STUB1* mutations who presented with the SCA48 phenotype (42). They also noted an association between dementia and shorter lifespan in patients carrying both *STUB1* mutations and intermediate TBP41-49 alleles, concluding that *STUB1* mutations are causal, whereas intermediate TBP41-49 alleles act as disease modifiers, leading to a more severe disease presentation (42). Our clinical association studies excluded patients with mutations in other known ataxia-related genes to better depict the pure *STUB1*-related phenotypes. Although our data provide key insights into CHIP domain-specific functions and their influence on SCA48 clinical presentation, additional research is needed to fully understand how other genetic factors, such as intermediate TBP41-49, operate within the SCA48 framework and contribute to the disease.

The scarcity of mutations in the coiled-coil domain (∼3% of reported SCA48 variants, despite comprising about 15% of *STUB1* length) is notable and suggests strong selective pressure (Fig. 8). This region, crucial for dimerization (25, 43, 44), may be highly intolerant to alterations, as disruptions could completely abolish CHIP function and potentially cause embryonic death or non-viable phenotypes, similar to homozygous *Stub1* knockout mice that die perinatally (31, 45). Alternatively, variants in the coiled-coil region might be tolerated but not cause disease in SCA48, thus escaping detection in disease cohorts. Without biochemical or clinical data on these mutations, definitive conclusions are challenging; however, the importance of this domain indicates that targeted sequencing in larger cohorts is necessary to determine whether these mutations are tolerated or lethal.

This domain-clinical mapping reflects patterns observed in other inherited ataxias and motor neuron diseases, where the mutation location influences phenotypic variation (46). In SCA3 (*ATXN3*), mutations in the N-terminal Josephin domain lead to earlier onset and more severe ataxia, while C-terminal variants near the polyQ tract result in milder forms with notable spasticity or dystonia (47). Likewise, in SCA2 (*ATXN2*), N-terminal expansions are associated with pure ataxia and slow saccades, whereas rare C-terminal disruptions are linked to Parkinsonism or ALS-like features. In ALS (*SOD1*), mutations in the N-terminal metal-binding domain often cause rapid progression with lower motor neuron (LMN) dominance, while C-terminal variants tend to have slower courses with upper motor neuron (UMN) signs (48, 49, 50, 51). In hereditary spastic paraplegia type 4 (SPG4), *SPAST* missense mutations in the C-terminal ATPase (AAA) domain are typically associated with pure spastic paraplegia primarily affecting upper motor neurons, whereas truncating or frameshift variants, often disrupting the overall protein function, including the microtubule-binding domain, are linked to complex phenotypes with additional features such as ataxia and dysarthria (52, 53, 54, 55, 56). These parallels support SCA48's model: TPR (upstream recognition) leads to aggressive UMN/ataxia symptoms, similar to N-terminal disruptions, while U-box (downstream ligase) results in more subtle bulbar involvement, resembling C-terminal effects.

Pathophysiologically, the universal cerebellar atrophy in SCA48 indicates a common core mechanism (32, 39), in which reduced CHIP function and altered oligomerization impair proteostasis and promote aggregate buildup in Purkinje and granule cells. Our biochemical and cellular data, together with the prior models of STUB1-related disease, support a working hypothesis that some SCA48 variants may interfere with wild-type CHIP within dimers and high-order complexes, thereby exerting dominant interference or gain-of-toxic effects. We emphasize that this remains a hypothesis: it has not been directly tested in heterozygous patient-derived systems, and demonstrating dominant-negative activity would require co-expression of wild-type and mutant CHIP (57, 58). Regarding UMN *versus* bulbar involvement, motor neuron diseases like ALS show proteostasis-related susceptibility: UMN vulnerability often stems from cortical ER stress due to trapped aggregates, consistent with TPR's client bottleneck, while bulbar/LMN strain involves chaperone overload and axonal transport issues, matching CHIP’s U-box failure (59, 60). The lack of differences in SARA severity (despite a small sample size of N = 27) supports cerebellar convergence, but larger groups might reveal subtle gradients (61, 62).

Several limitations of the present study warrant explicit discussion. First, our cellular assays were performed in COS-7 and HEK-293 cells using transient overexpression of single CHIP variants, which do not recapitulate the heterozygous state of SCA48 patients, who co-express one wild-type and one mutant *STUB1* allele. Accordingly, we present possible dominant interference and gain-of-toxic effects as working hypotheses derived from the biochemical and cellular signatures of individual variants, rather than as established mechanisms. Dominant-negative activity in the formal sense, active suppression of wild-type CHIP function by the mutant protein, requires co-expression of both alleles to demonstrate; formal testing therefore awaits heterozygous cellular or animal models of SCA48. Second, our sh*STUB1* RNA-seq experiments model CHIP insufficiency rather than the consequences of specific mutant expression. Third, neither COS-7 nor HEK-293 cells recapitulate the proteostatic environment of cerebellar Purkinje neurons, the primary cell type affected in SCA48. Fourth, mass photometry was performed at low nanomolar concentrations; while a new concentration series (Fig. S3) confirms that the three-profile oligomeric classification is robust across 20 to 100 nM, intracellular concentrations and macromolecular crowding remain outside the directly accessible range, and we therefore frame the MP-based classification as a biochemical fingerprint of variant assembly tendency rather than as a precise prediction of in-cell stoichiometry. Fifth, the non-discrete high-molecular-weight species formed by several U-box mutants may represent either ordered higher-order oligomers (potentially retaining partial activity) or amorphous aggregates (potentially proteotoxic); the present biophysical methods do not distinguish these possibilities. Finally, the SARA score comparison (N = 27) is underpowered and reported as such; the remaining phenotype-domain associations (UMN signs and ITR predominance in TPR carriers, near-universal gait ataxia in both domains, dysarthria predominance in U-box carriers) survive BH 5% FDR correction despite the inherent constraints of retrospective multi-center reporting and ascertainment bias.

Finally, these insights open the door for targeted therapies in SCA48 and related ubiquitin ligase disorders (63). Stabilizing TPR-client interactions could mitigate early proteotoxic stress, similar to how ATXN3 inhibitors targeting deubiquitinase activity reduce aggregate formation and improve motor symptoms in SCA3 models (64, 65). For U-box mutations, restoring ligase activity with small-molecule activators of E3 ligases (like PROTACs that recruit CHIP) or chaperone modulators (such as HSP70 inhibitors to reduce overload) shows promise (66, 67). This approach has been demonstrated in ALS trials targeting SOD1 ubiquitination and in Alzheimer’s disease models, where PROTACs degrade tau aggregates by recruiting E3 ligases such as VHL or CRBN, thereby decreasing neurotoxicity (68, 69). Beyond these CHIP-directed strategies, allele-specific approaches such as antisense oligonucleotide silencing or CRISPR-based domain editing of mutant STUB1 alleles may eventually become tractable. All such strategies remain speculative pending a tractable *in vivo* model of SCA48; the present work contributes by providing a domain-specific biochemical and clinical framework for prioritizing candidate interventions.

## Experimental procedures

### Plasmids, mutagenesis, and protein purification

We used the pcDNA3-CHIP-myc and pET30-CHIP-His plasmids for mammalian and bacterial expression, respectively, as described earlier (19). Disease-related mutations were introduced using the Q5 Site-Directed Mutagenesis Kit (New England Biolabs, E0554S) following the manufacturer’s instructions, with primers listed in Table S4.

CHIP mutants were expressed with a C-terminal His tag in BL21 (DE3)-RIL cells. Cells were resuspended and lysed in 1X PBS, 20 mM imidazole, 5 mM BME. Proteins for mass photometry were purified *via* nickel affinity chromatography and buffer exchanged into 20 mM HEPES pH 8.0, 200 mM NaCl, 1 mM DTT using Cytiva PD-10 desalting columns. Proteins for ubiquitination assays were purified *via* nickel affinity chromatography followed by size exclusion chromatography in 20 mM HEPES pH 8.0, 200 mM NaCl, 1 mM DTT.

Fluorescently labeled HSP70 (∗HSP70) was prepared using a GST-tagged, sortase-compatible HSP70 construct. This construct was expressed similarly to CHIP but was resuspended and lysed in 50 mM Tris pH 7.6, 200 mM NaCl, and 5 mM BME. The protein was purified through glutathione affinity chromatography, and the GST tag was cleaved with PreScission-GST. Uncleaved protein was removed by an additional round of glutathione affinity chromatography. GST-cleaved HSP70, which contains an N-terminal Glycine residue, was fluorescently labeled *via* an overnight reaction with 5 μM protein, 50 μM fluorescent peptide, and 5 μM His-sortase. The reaction was quenched with 10 mM EDTA, then passed through a Cytiva PD-10 desalting column and nickel affinity chromatography to remove excess peptide and His-sortase. Finally, ∗HSP70 was further purified by size exclusion chromatography using 20 mM HEPES pH 8.0, 200 mM NaCl, and 1 mM DTT. Non-labeled HSP70 and HSP40 were purified as previously described (17).

### Cell culture, transfection, and heat shock induction

COS-7 (ATCC CRL-1651) and HEK-293 (ATCC CRL-1573) cells were obtained directly from the American Type Culture Collection and used within 20 passages of thawing; no additional STR profiling was performed. Cells were cultured in Dulbecco’s Modified Eagle’s Medium (Corning, 10-017-CV) supplemented with 10% fetal bovine serum (Gibco, 26140-079) and incubated at 37 °C in a 5% CO_2_ atmosphere. Transient transfections were performed using X-tremeGENE 9 DNA transfection reagent (Roche, 6365787001) following the manufacturer’s instructions, with a 1:3 DNA to reagent ratio. Heat shock was applied by incubating cells at 42 °C for 2 h in a 5% CO_2_ environment. Stable knockdown of *STUB1*/CHIP was achieved using a pooled combination of four validated shRNA lentiviruses (TRCN0000007525, TRCN0000007526, TRCN0000007528, TRCN0000007529) as previously published; target sequences are given below under RNA sequencing.

### Mass photometry

Mass photometry was conducted using a Refeyn OneMP Mass Photometer placed on an Accurion i4 vibration isolation system. Samples were prepared by washing ThorLabs Precision microscope cover slips through sonication for 10 min in a 50:50 mixture of Isopropanol and DI water, followed by 10 min in 100% DI water. The cleaned cover slips were stored in DI water and dried immediately before use with filtered air. CultureWell gaskets (Grace Bio-Labs) were glued to the dried cover slips. The mass photometer lens was prepped by adding a drop of Olympus IMMOIL-F30CC immersion oil, after which the prepared cover slip was placed on top. For each well, 15 μl of filtered 20 mM HEPES buffer (pH 8.0) containing 200 mM NaCl was added, and the lens was focused near the edge of the well using the droplet-dilution method. Once proper focus was achieved, 5 μl of a 200 nM prediluted CHIP sample was added to the well, resulting in a total volume of 20 μl and a final concentration of 50 nM (a subset of proteins was tested at additional concentrations: 20 nM and 100 nM). The signal was briefly allowed to stabilize, then a 60-s video was recorded at 100 frames per second using the AcquireMP software. The video was processed in DiscoverMP, and a mass calibration curve was created using BSA (Sigma P0834) and ApoFerritin (Sigma A3660) to estimate the mass of each event. The analyzed events were then processed in Python to generate normalized bar and scatter plots, which were visualized using GraphPad Prism.

### HSP70 ubiquitination and autoubiquitination assays

HSP70 ubiquitination assays were performed using HSP70 tagged with a fluorescent N-terminus. All assay components except CHIP were premixed on ice in a BSA-containing buffer (20 mM HEPES, pH 8.0, 200 mM NaCl, 0.5 mg/ml BSA). Reactions were initiated by adding CHIP or a mixture of MgCl2-ATP, ∗HSP70, and UBA1, and kept at room temperature unless otherwise specified. The reactions contained 1 μM UBA1, 5 μM, 25 μM, or 100 μM UBE2D2, 1 μM ∗HSP70, 100 μM Ub, 5 mM MgCl2-ATP, and 5 μM CHIP. They were allowed to proceed for 15 min before being quenched with SDS loading buffer. The quenched samples were separated by molecular weight using SDS-PAGE under non-reducing conditions with GenScript SurePAGE 4 to 12% gels. Fluorescent scanning on an Amersham Typhoon 5 enabled visualization and quantification of CHIP-dependent ubiquitination of ∗HSP70.

CHIP autoubiquitination was monitored through pulse-chase assays. First, a 6x pulse mixture of 0.1 μM UBA1, 10 μM UBE2D2, 10 μM ∗Ub, and 5 mM MgCl2-ATP was mixed on ice to form UBE2D2∼∗Ub (“∼” denotes a thioester intermediate). The UBE2D2∼∗Ub mixture was incubated at room temperature for 10 min, then quenched with 50 mM EDTA. Chase mixes were prepared with 5 μM of either wild-type or variant CHIP, and the reaction was started by adding the pulse mix to a 1x concentration. The reactions were allowed to proceed at room temperature and quenched with SDS loading buffer at the indicated time points. Quenched samples were analyzed by SDS-PAGE and fluorescent scanning, as described above for substrate ubiquitination assays.

### Bio-layer interferometry

Bio-layer interferometry (BLI) experiments were performed using a BLItz instrument (FortéBio) to study the interaction between UBE2D2 and different CHIP mutants. Protein concentrations were measured using a Pierce 660 nm protein assay (ThermoFisher) and diluted to the required concentrations. An anti-Penta-HIS (HIS1K, Sartorius) biosensor was used for loading the His-tagged CHIP wild-type and mutant proteins onto the biosensors, and all experiments were carried out at 25 °C in a temperature-controlled incubator. The biosensors were first hydrated in 200 μl of buffer (20 mM HEPES, 50 mM NaCl, pH 7.5) for 10 min. After hydration, the biosensor was attached to the tip of the BLItz instrument for the experiment. The initial baseline was conducted for 30 s with the equilibration buffer (50 mM HEPES, 50 mM NaCl, pH 7.5) in a 500 μl opaque amber microcentrifuge tube. Next, 4 μl of His-tagged CHIP at 5 μM concentration was pipetted into the drop holder, and the loading step was conducted for 120 s. The biosensor was then incubated in the equilibration buffer for another 30 s to establish a second baseline. Next, 4 μl of UBE2D2 was added, and the association step was carried out for 120 s to measure the interaction between the CHIP variant and UBE2D2. Finally, the dissociation step was performed by incubating the biosensor in equilibration buffer for 120 s. The UBE2D2 construct used for the BLItz experiments was expressed from a pHis//2 Hs UBE2D2 (1–147) plasmid and purified as previously described, including tag removal using Tobacco Etch Virus protease (70, 71). To determine the binding affinity (*K*_*d*_), BLI measurements were performed at multiple UBE2D2 concentrations (25, 50, 75, and 100 μM) during the association step. The same procedure was repeated for all His-tagged CHIP variants. The resulting data were analyzed using BLItz software to calculate the binding affinity from the locally fitted association and dissociation rates of each replicate.

### Cell lysis and soluble–insoluble fractionation

Cells were maintained and transfected as described above. Twenty-four hours after transfection, cells were lysed in ice-cold 1% Triton X-100 buffer (50 mM Tris-HCl pH 7.4, 150 mM NaCl, 2 mM EDTA, 1% Triton X-100) supplemented with 1X Halt protease and phosphatase inhibitor (Thermo Scientific, 78447). Lysates were cleared by centrifugation at 15,000 rpm for 10 min. BCA assay (Thermo Scientific, 23223 & 23224) was performed to determine protein concentrations, and 50 μg of total protein was used for immunoblotting.

For soluble and insoluble fractionation, cells were lysed in ice-cold 1% Triton X-100 buffer (50 mM Tris-HCl pH 7.4, 150 mM NaCl, 2 mM EDTA, 1% Triton X-100) supplemented with 1X Halt protease and phosphatase inhibitors (Thermo Scientific, 78447). Lysates were cleared by centrifugation at 16,000 × *g* for 30 min at 4 °C. The supernatants (Triton X-100 soluble fraction) were transferred to a fresh tube. The pellets were washed with lysis buffer and centrifuged at 16,000 × *g* for 10 min at 4 °C. Lysis buffer was discarded, and the pellets were resuspended in 1X Laemmli sample buffer (Bio-Rad, 1610747). Samples were briefly sonicated, and protein concentrations were measured (Thermo Scientific, 22660). 50 micrograms of Triton X-100 soluble and insoluble fractions were used for immunoblotting.

### Immunoblotting, immunoprecipitation, and antibodies

Cells were lysed as described above, and protein concentrations were measured using the BCA Protein Assay Kit (Thermo Scientific, 23223 & 2,224). Equal amounts of total protein (15–50 μg) were mixed with 1X Laemmli sampling buffer (Bio-Rad, 1610747) and boiled at 100 °C for 5 min. Proteins were separated on 4 to 15% Mini-PROTEAN TGX Stain-Free gels (Bio-Rad, 4568084) and then transferred to PVDF membranes (Bio-Rad, 1620264). Membranes were blocked with 5% milk or bovine serum albumin (BSA) and incubated overnight at 4 °C with primary antibodies diluted in the appropriate blocking solution (Table S5). After washing in TBST, membranes were incubated with HRP-conjugated anti-rabbit secondary antibody (Cell Signaling Technology, 7074), and visualized using Lumigen ECL Ultra (Lumigen, tma-100). For fluorescent Western blot detection, primary antibodies against phospho-CHIP (Ser20) (Abmart Inc., 25011-1) and MYC (Proteintech, 60003-2-Ig) were used, followed by fluorescent secondary antibodies StarBright 700 (Bio-Rad, 12004161) or StarBright 520 (Bio-Rad, 12005866). For immunoprecipitation, 10E6 cells were plated in 10 cm tissue culture dishes and transfected as described above. Twenty-four hours post-transfection, cells were lysed in ice-cold 1% Triton X-100 buffer (50 mM Tris-HCl pH 7.4, 150 mM NaCl, 2 mM EDTA, 1% Triton X-100) supplemented with 1X Halt Protease and Phosphatase Inhibitor Cocktail (Thermo Scientific, 78447). Lysates were cleared by centrifugation at 15,000 rpm for 10 min, and protein concentrations were measured using a BCA assay. For each sample, 1 mg of total protein was used for immunoprecipitation. CHIP-myc and Flag-HSP70 were pulled down with Ezview Red Anti-c-Myc affinity gel (Sigma-Aldrich, E6654) and Ezview Red Anti-Flag M2 affinity gel (Sigma-Aldrich, F2426), respectively, following the manufacturer’s instructions. Cell lysates were incubated with affinity gels overnight at 4 °C with rotation. Beads were washed 5 times before elution with 1X Laemmli sampling buffer containing 1% Triton X-100, then boiled at 100 °C for 5 min. Eluted samples and inputs were run on 4 to 15% Mini-PROTEAN TGX Stain-Free gels and transferred to PVDF membranes for immunoblotting as described above.

### Immunofluorescence

COS-7 cells were fixed with 4% paraformaldehyde (PFA) for 15 min at room temperature, then washed three times with 1× phosphate-buffered saline (PBS). Fixed cells were either stored at 4 °C or processed immediately for immunofluorescence staining. For blocking and permeabilization, cells were incubated for 60 min at room temperature in 5% bovine serum albumin (BSA; Fisher Scientific, BP9703-100) diluted in PBS containing 0.1% Triton X-100. Subsequently, cells were incubated overnight at 4 °C with primary antibodies against Myc (Proteintech, 60003-2-Ig, D1I4Q) and HSF1 (Cell Signaling Technology, #4356), both diluted in 1% BSA with 0.1% Triton X-100.

Following primary antibody incubation, cells were washed three times with PBS containing 0.1% Triton X-100 for 10 to 15 min each. Samples were then incubated for 1 h at room temperature with Multi-rAb CoraLite Plus 488-Goat Anti-Rabbit Recombinant Secondary Antibody (H + L) and Multi-rAb CoraLite Plus 555-Goat Anti-Mouse Recombinant Secondary Antibody (H + L) (Proteintech), both diluted in 1% BSA with 0.1% Triton X-100. After secondary antibody incubation, cells were rinsed three times with PBS containing 0.1% Triton X-100. During the second wash, NucBlue Fixed Cell ReadyProbes Reagent (Invitrogen, MA, USA) was added to stain nuclei.

After the final wash, samples were mounted on glass slides and covered with #1.5 coverslips using ProLong Diamond Antifade Mountant (Thermo Fisher Scientific). Immunofluorescence images were captured with a Nikon Eclipse Ti2 microscope equipped with a 40×/0.95 NA Plan Apochromat air objective and controlled by Nikon Elements software. Fluorescence signals were recorded using a pco.edge 4.2Q High QE sCMOS camera, providing high sensitivity and resolution for quantitative image analysis.

#### Image analysis

For each condition, images from three fields were blinded and randomly selected per biological replicate. Images were included for analysis conditionally if they had visible boundaries of the target structure. Images were processed and analyzed using the ImageJ/FIJI software. The nuclear localization of Myc and HSF1 was quantified by comparing nuclear to cytoplasmic intensity ratios or by normalizing nuclear Myc or HSF1 intensity to the total nuclear signal (for example, nuclear Myc/nuclear NucBlue intensity). In the figures, the nuclear intensity of Myc or HSF1 is shown using a masked nuclear region created based on NucBlue nuclear staining, with the intensity displayed in a 16-color LUT (FIJI).

### Antibody validation

All antibodies used in this study were commercially obtained and used at the manufacturer-recommended dilutions; the vendor, catalog number, and dilution for each antibody are listed in Table S5. For epitope-tag antibodies (anti-myc and anti-Flag), specificity was confirmed by parallel staining or immunoblotting of vector-only (untransfected) negative controls, which showed no specific signal at the expected molecular weight. For all other antibodies, vendor-supplied validation data (Western blot specificity in relevant cell lines, knockout/knockdown controls, and/or peptide blocking) were relied on.

### Blue native polyacrylamide gel electrophoresis

COS-7 cells were maintained and transfected as described above. Twenty-four hours after transfection, samples were collected in Native PAGE sample buffer (Invitrogen, BN2003) supplemented with 1X Halt Protease and Phosphatase Inhibitors cocktail (Thermo Scientific, 78447) and 1% digitonin (Calbiochem, 300410). Lysates were clarified by centrifugation at 20,000×*g* for 30 min at 4 °C. Protein concentrations were measured using a BCA assay. G-250 Sample Additive (Invitrogen, BN2004) was added to the samples, and blue native PAGE was run using the dark blue and light blue cathode buffer protocol according to the manufacturer’s instructions (Invitrogen). Native protein samples were resolved on 18-well 4% to 15% Criterion TGX Stain-Free Precast Gels (Bio-Rad, 5678084) using 0.25% G-250 cathode buffer (Invitrogen, BN2007) on ice and transferred to PVDF membrane. Immunoblot analysis was performed as described above.

### Cycloheximide chase assay

COS-7 cells were maintained as described earlier. Twenty-four hours post-transfection, cells were treated with 50 μg/ml cycloheximide at the specified time points and then harvested in Triton X-100 buffer. 50 μg of total protein were separated by SDS-PAGE as described earlier, followed by immunoblot analysis. Densitometry values were normalized to the t = 0 time point and log-transformed. Protein turnover was modeled using a mixed-design log-linear regression of ln(CHIP intensity) on time, with biological replicate included as a fixed-effect block (n = 3 biological replicates, technical duplicates). Because the derived half-lives are nonlinear functions of the fitted slopes and have wide confidence intervals, differences in stability between variants were evaluated using the variant × time interaction terms rather than pairwise comparisons of half-lives, with multiple-comparison correction by the Holm–Bonferroni method. Half-lives (t½ = ln2/|slope|) are reported as descriptive summaries with 95% confidence intervals.

### Luciferase refolding assay

Recombinant CHIP, HSP70, and HSP40 proteins were purified as described above. Recombinant luciferase (Promega, E1701) was prepared at 0.2 μM in 2 mg/ml BSA (Thermo Fisher Scientific, BP9706100) and heat-denatured by incubation at 42 °C for 15 min. Recombinant CHIP, HSP70, and HSP40 proteins were diluted to 32 μM in HEPES protein storage buffer. Denatured luciferase and recombinant proteins were diluted 1:4 in protein storage buffer and incubated at 37 °C for an hour to allow refolding. Three replicates of 2 μl reaction mix were aliquoted into a white opaque 96-well plate. A CLARIOstar plate reader (BMG Labtech) was used to inject 50 μl Luciferase Assay Reagent (Promega, E1483) into each well, followed by luminescence detection.

### HSF1 luciferase reporter assay

HSF1 luciferase reporter assay was conducted as previously described (19). Briefly, cells were transfected with the indicated vectors alongside the Qiagen Heat Shock Response Reporter Kit (Qiagen, CCS4023L) according to the manufacturer’s instructions. Twenty-four hours after transfection, cells were lysed with 1X passive lysis buffer (Promega, E1910), and the luciferase assay was performed using the Dual-Luciferase Reporter Assay System (Promega, E1910) on a CLARIOstar plate reader following the manufacturer’s protocol. Three biological replicates, each with three technical replicates, were conducted on separate days. Luciferase/Renilla ratios were calculated, and technical replicates were averaged. Ratios were normalized to wells transfected with empty vector.

### Differential scanning fluorometry

Recombinant CHIP proteins were purified as described above. Reaction buffers were prepared by diluting the indicated CHIP proteins to 20 μM in 10X SYPRO Orange (Sigma-Aldrich, S5692) in HEPES protein storage buffer. Three replicates of 10 μl reaction buffer were aliquoted into 384-well PCR plates, and the melting temperature assay was performed on a LightCycler 480 (Roche) following the manufacturer’s protocol. Fluorescence was measured at 568 nm while the temperature was ramped from 20 °C to 85 °C with 10 acquisitions per degree Celsius. The melting temperatures of CHIP proteins were determined by calculating the first derivative of the fluorescence data and identifying the local maxima. Differential scanning fluorimetry was performed in three independent experiments on separate days. For each experiment, technical triplicates were measured for every variant.

### Densitometry analysis

Immunoblots were detected using Bio-Rad ECL substrate or the indicated Bio-Rad fluorescent secondary antibodies and imaged on a Bio-Rad ChemiDoc system, with exposure times kept within the detector's linear range. Stain-free total protein was imaged on the same instrument per the manufacturer’s protocol. Immunoblot quantification was performed by densitometry using Bio-Rad Image Lab software (v6.1).

### RNA sequencing and differential expression analysis

#### Cell culture, heat shock, and RNA isolation

HEK-293 cells were cultured in DMEM supplemented with 10% FBS and 1% penicillin-streptomycin at 37 °C in 5% CO_2_. Stable STUB1 knockdown (shCHIP) and non-targeting control (shControl) lines were generated as previously described (72). The shControl line was generated using the MISSION pLKO.1-puro non-mammalian non-targeting shRNA control (SHC002; Sigma-Aldrich), which carries a hairpin with no homology to any known mammalian transcript. Knockdown was achieved using a lentiviral shRNA library consisting of four independently validated hairpins in the pLKO.1-puro backbone (MISSION shRNA particles; Sigma-Aldrich), each targeting human STUB1 (NM_005861). Target sequences (5′→3′) were: TRCN0000007525, CCCAAGTTCTGCTGTTGGACT (3′-UTR, position 1069); TRCN0000007526, GAAGAGGAAGAAGCGAGACAT (CDS, position 716); TRCN0000007528, GCAGTCTGTGAAGGCGCACTT (CDS, position 329); and TRCN0000007529, CGCGAAGAAGAAGCGCTGGAA (CDS, position 479). Each hairpin has the structure 5′-CCGG-[target sequence]-CTCGAG-[reverse complement of target]-TTTTT-3′. Lentiviral particles were pooled to minimize off-target effects, and knockdown efficiency was confirmed in pilot experiments by qPCR (>75% reduction in STUB1 mRNA) and immunoblotting (>75% reduction in CHIP protein levels) (Fig. S6, *A*–*C*). Stable cells were selected with puromycin (2 μg/ml) for 72 h. For heat shock experiments, cells were incubated at 42 °C for 30 min. Three independent biological replicates were prepared for each of four conditions: shControl 37 °C, shControl 42 °C, shCHIP 37 °C, and shCHIP 42 °C (n = 12 samples total). Total RNA was isolated using the RNeasy Mini Kit (Qiagen) with on-column DNase I digestion. RNA purity was assessed by NanoDrop (A260/A280 ≥ 2.0, A260/A230 ≥ 1.8), and integrity (RIN ≥ 9.0) was confirmed using the RNA 6000 Nano Kit on a 2100 Bioanalyzer (Agilent).

#### Library construction and sequencing

Poly(A)-selected mRNA libraries were prepared using the Illumina TruSeq RNA Sample Preparation Kit. Sequencing was performed single-end 50 cycles on an Illumina HiSeq 2500, producing 15.8 to 36.4 million read pairs per sample (average: 24.7 million). Raw data quality was confirmed using FastQC v0.11.9 (per-base Phred score >30 in at least 95% of cycles).

#### Read alignment and quantification

Reads were aligned to the human reference genome (GRCh38.p13) using STAR v2.7.8a in 2-pass mode with default parameters. Alignment was guided by Ensembl transcript annotations (release 105). Gene-level read counts were quantified using Partek Expectation/Maximization (E/M) in Partek Flow v10.0.23.0214, counting only uniquely mapped, properly paired reads overlapping annotated exons. Initial quantification included 27,173 genes.

*Differential expression analysis* was performed in Partek Flow v10.0.23.0214 using the DESeq2 node with median ratio normalization. A low-count filter was applied: genes with less than 1 count per million (CPM) in at least 30% of samples were removed, resulting in 15,475 genes retained (11,698 genes were filtered out). The linear model included genotype (shControl, sh*STUB1*), temperature (37 °C, 42 °C), and their interaction.

$$\log_{2}\left(normalized\;count\right)∼genotype+temperature+genotype:temperature$$

Wald tests were performed for the following contrasts.

• shCHIP *versus* shControl (37 °C)
• 42 °C *versus* 37 °C (shControl)
• 42 °C *versus* 37 °C (shCHIP)
• shCHIP 42 °C *versus* shControl 42 °C
• shCHIP 37 °C *versus* shControl 37 °C
• Interaction (shCHIP 42 °C *versus* shCHIP 37 °C) *versus* (shControl 42 °C *versus* shControl 37 °C):

FDR step-up (Benjamini–Hochberg) correction was applied. Independent filtering was enabled; fold change shrinkage was not used. Differentially expressed genes (DEGs) were identified as having FDR < 0.05 in heat shock contrasts (42 °C *versus* 37 °C). This process identified 886 heat-responsive genes in shControl (573 unique), 423 in sh*STUB1* (110 unique), and 313 genes in common (overlap). Variance-stabilized counts were used for downstream visualization.

Raw and processed RNA sequencing data, including FASTQ files and gene-level counts, have been deposited in the NCBI Gene Expression Omnibus (GEO) under accession number GSE310700.

#### Functional enrichment analysis

Three gene sets were analyzed using g:Profiler (g:GOSt functional profiling, ens109) in multi-query mode.

1. shControl-unique DEGs (n = 573)
2. shCHIP-unique DEGs (n = 110)
3. Overlap DEGs (n = 313)

The background gene set comprised 15,476 filtered genes. Enrichment analysis was performed using Gene Ontology (Biological Process, Molecular Function, Cellular Component) and Reactome pathways. To prioritize biologically relevant pathways for SCA48, Grok AI (xAI) was queried with the following prompt: “Given the following g:Profiler enrichment results for the multi-query, prioritize terms that are relevant to SCA48 pathophysiology. Focus on pathways related to protein homeostasis, stress response (particularly heat shock and HSF1 signaling), ubiquitination/proteostasis, transcriptional regulation, and neuronal or cerebellar function. Highlight terms with strong enrichment in the overlap or shControl conditions but attenuated in shSTUB1.” AI suggestions were manually curated by the authors using three criteria (1): adjusted *p*-value < 0.05 in at least one condition (2), biological relevance to CHIP/STUB1 function or SCA48 phenotypes, and (3) differential enrichment pattern (stronger in overlap/control *versus* shSTUB1). The full unfiltered g:Profiler output is provided in Table S6. Results passing an FDR < 0.05 cutoff were visualized as a heatmap of −log_10_(FDR) values across the three conditions.

#### Identification of HSF1 target genes

Candidate HSF1 targets were identified by systematically cross-referencing multiple high-confidence, publicly available sources of direct HSF1 targets in human cells. Direct targets were defined as genes with evidence of HSF1 binding (ChIP-seq/ChIP-chip peaks near the transcription start site) and/or HSF1-dependent transcriptional regulation under proteotoxic stress (heat shock, proteostasis challenge) or in cancer contexts. Genes were retained only if supported by at least two independent sources (ENCODE, ChIP-Atlas, HSF1Base, and the non-canonical HSF1 regulon) to ensure robustness and reduce false positives (73, 74, 75, 76, 77, 78, 79, 80).

### Meta-analysis of SCA48 clinical phenotypes

#### Data collection and cohort assembly

Clinical and genetic data from 87 SCA48 patients with heterozygous *STUB1* mutations were compiled from eight published studies across multiple geographic regions (Table S3). Inclusion required a confirmed pathogenic variant (missense, frameshift, nonsense, or splice-site), at least one documented clinical feature, and a diagnosis of cerebellar ataxia. Extracted variables included age of onset (AOO, years), sex, *STUB1* cDNA and protein changes, exon, domain, SARA score, and the presence of gait ataxia (Y/N), dysarthria (Y/N), cognitive dysfunction (Y/N), upper motor neuron dysfunction (UMND; Y/N), increased tendon reflexes (ITR; Y/N), and nystagmus (Y/N). Cerebellar atrophy was consistently reported (100%) when used as a clinical diagnostic criterion but was not included in association analyses. Patients with pathogenic or likely pathogenic variants in other known ataxia-associated genes, including intermediate TBP repeat expansions, were excluded from all genotype-phenotype association analyses so that the reported associations reflect STUB1 genotype alone. Clinical features were coded as present (Y) or absent (N) only when the source publication explicitly documented the feature as present or absent; features not reported or reported ambiguously were coded as N/A. Missing documentation was never treated as evidence of symptom absence. N/A entries were excluded listwise on a per-feature basis, so the denominator varies by comparison; the analyzed N for each feature is given in Table S3 and in the corresponding figure legends (for example, ITR, N = 42; SARA, N = 27). No imputation was performed. Because incomplete reporting is unlikely to be independent of phenotype severity, this approach is conservative with respect to prevalence estimates but remains subject to ascertainment bias, as noted in the Discussion.

#### Mutation annotation and domain assignment

Variants were annotated relative to the canonical *STUB1* transcript (ENST00000219548.9). Domain boundaries were assigned based on structural and functional data: TPR (aa 1–126), CC (aa 127–221), and U-box (aa 222–303). Exon/AA boundaries are 1 (1, 2, 3, 4, 5, 6, 7, 8, 9, 10, 11, 12, 13, 14, 15, 16, 17, 18, 19, 20, 21, 22, 23, 24, 25, 26, 27, 28, 29, 30, 31, 32, 33, 34, 35, 36, 37, 38, 39, 40, 41, 42, 43, 44, 45, 46, 47, 48, 49, 50, 51, 52, 53), 2 (54–120), 3 (121–175), 4 (176–204), 5 (205–223), 6 (224–262), and 7 (263–303). Frameshift and nonsense variants were classified by the domain containing the premature termination codon.

#### Statistical analyses

Analyses were performed using JMP Pro v19 (SAS Institute). Two-sided *p*-values less than 0.05 were considered statistically significant.

##### Mutation distribution and exon intolerance

Expected mutation frequencies per exon were calculated as:

$$E_{i}=N\times \frac{L_{i}}{\sum L_{j}}$$

where N = total mutations and $L_{i}$ = coding length of exon i. Observed *versus* expected counts were compared using chi-square goodness-of-fit with Bonferroni correction across seven exons.

##### Age of onset and disease severity

AOO was compared between TPR and U-box mutation groups using a two-tailed Student’s *t* test, which was confirmed to be normal by the Shapiro–Wilk test (W = 0.98, *p* = 0.4921). SARA scores were categorized into five severity levels (minimal: 1–9; moderate: 10–12; maximal: 13–17; severe: 18–24; total: 25–40). Domain associations were analyzed with a contingency table using Fisher’s exact test.

##### Categorical symptom associations

Six binary clinical features (ataxia, dysarthria, cognitive dysfunction, UMND, ITR, nystagmus) were tested against the domain (TPR *versus* U-box) using 2 × 2 contingency tables and Fisher’s exact test. *p*-values were adjusted with the false discovery rate (FDR; Benjamini–Hochberg) across the tests.

##### Sensitivity and Limitations

Due to the retrospective, multinational design, variation in clinical assessment timing, diagnostic criteria, and reporting depth was inevitable. Results should be viewed as hypothesis-generating, pending validation in prospective, standardized cohorts.

### Statistical analysis software

All statistical analyses of the biochemical and clinical data were performed using GraphPad Prism 9 and JMP Pro (v19.0.0), respectively. Per-figure-panel statistical reporting, including test, test statistic, exact and adjusted *p*-values, and sample size for each comparison, is tabulated in Table S1.

## Data availability

Raw and processed RNA-sequencing data have been deposited in NCBI Gene Expression Omnibus under accession GSE310700 and are publicly available. All other data supporting the findings of this study are available within the article and its supplementary information files, or from the corresponding authors upon reasonable request.

## Supporting information

This article contains supporting information.

## Conflict of interest

The authors declare that they have no conflicts of interest with the contents of this article.

## References

[1] Wolff S., Weissman J.S., Dillin A. (2014). Differential scales of protein quality control. Cell.

[2] Ronnebaum S.M., Patterson C., Schisler J.C. (2014). Emerging evidence of coding mutations in the ubiquitin-proteasome system associated with cerebellar ataxias. Hum. Gen. Variation..

[3] Shi C.-H., Schisler J.C., Rubel C.E., Tan S., Song B., McDonough H. (2014). Ataxia and hypogonadism caused by the loss of ubiquitin ligase activity of the U box protein CHIP. Hum. Mol. Genet..

[4] Genis D., Ortega-Cubero S., San Nicolás H., Corral J., Gardenyes J., de Jorge L. (2018). Heterozygous STUB1 mutation causes familial ataxia with cognitive affective syndrome (SCA48). Neurology.

[5] Baviera-Muñoz R., Carretero-Vilarroig L., Pedro-Ibor A., Jaijo T., Del Valle-Carranza A., Martínez-Torres I. (2024). STUB1 mutations as possible genetic modifiers in spinocerebellar ataxia type 8. Mov. Disord..

[6] Magri S., Nanetti L., Gellera C., Sarto E., Rizzo E., Mongelli A. (2022). Digenic inheritance of STUB1 variants and TBP polyglutamine expansions explains the incomplete penetrance of SCA17 and SCA48. Genet. Med..

[7] Kampinga H.H., Kanon B., Salomons F.A., Kabakov A.E., Patterson C. (2003). Overexpression of the cochaperone CHIP enhances Hsp70-dependent folding activity in mammalian cells. Mol. Cell. Biol..

[8] Ballinger C.A., Connell P., Wu Y., Hu Z., Thompson L.J., Yin L.Y. (1999). Identification of CHIP, a novel tetratricopeptide repeat-containing protein that interacts with heat shock proteins and negatively regulates chaperone functions. Mol. Cell. Biol..

[9] Jiang J., Ballinger C.A., Wu Y., Dai Q., Cyr D.M., Höhfeld J. (2001). CHIP is a U-box-dependent E3 ubiquitin ligase: identification of Hsc70 as a target for ubiquitylation. J. Biol. Chem..

[10] Murata S., Minami Y., Minami M., Chiba T., Tanaka K. (2001). CHIP is a chaperone-dependent E3 ligase that ubiquitylates unfolded protein. EMBO Rep..

[11] Al-Ramahi I., Lam Y.C., Chen H.-K., de Gouyon B., Zhang M., Pérez A.M. (2006). CHIP protects from the neurotoxicity of expanded and wild-type ataxin-1 and promotes their ubiquitination and degradation. J. Biol. Chem..

[12] Shin Y., Klucken J., Patterson C., Hyman B.T., McLean P.J. (2005). The co-chaperone carboxyl terminus of Hsp70-interacting protein (CHIP) mediates alpha-synuclein degradation decisions between proteasomal and lysosomal pathways. J. Biol. Chem..

[13] Dickey C.A., Kamal A., Lundgren K., Klosak N., Bailey R.M., Dunmore J. (2007). The high-affinity HSP90-CHIP complex recognizes and selectively degrades phosphorylated tau client proteins. J. Clin. Invest..

[14] Jana N.R., Dikshit P., Goswami A., Kotliarova S., Murata S., Tanaka K. (2005). Co-chaperone CHIP associates with expanded polyglutamine protein and promotes their degradation by proteasomes. J. Biol. Chem..

[15] Paulson H.L., Shakkottai V.G., Clark H.B., Orr H.T. (2017). Polyglutamine spinocerebellar ataxias - from genes to potential treatments. Nat. Rev. Neurosci..

[16] Zhang S., Hu Z.-W., Mao C.-Y., Shi C.-H., Xu Y.-M. (2020). CHIP as a therapeutic target for neurological diseases. Cell Death Dis..

[17] Rosser M.F.N., Washburn E., Muchowski P.J., Patterson C., Cyr D.M. (2007). Chaperone functions of the E3 ubiquitin ligase CHIP. J. Biol. Chem..

[18] Schisler J.C., Rubel C.E., Zhang C., Lockyer P., Cyr D.M., Patterson C. (2013). CHIP protects against cardiac pressure overload through regulation of AMPK. J. Clin. Invest..

[19] Shi C.-H., Rubel C., Soss S.E., Sanchez-Hodge R., Zhang S., Madrigal S.C. (2018). Disrupted structure and aberrant function of CHIP mediates the loss of motor and cognitive function in preclinical models of SCAR16. Plos Genet..

[20] Madrigal S.C., McNeil Z., Sanchez-Hodge R., Shi C.-H., Patterson C., Scaglione K.M. (2019). Changes in protein function underlie the disease spectrum in patients with CHIP mutations. J. Biol. Chem..

[21] Umano A., Fang K., Qu Z., Scaglione J.B., Altinok S., Treadway C.J. (2022). The molecular basis of spinocerebellar ataxia type 48 caused by a de novo mutation in the ubiquitin ligase CHIP. J. Biol. Chem..

[22] Roux T., Barbier M., Papin M., Davoine C.-S., Sayah S., Coarelli G. (2020). Clinical, neuropathological, and genetic characterization of STUB1 variants in cerebellar ataxias: a frequent cause of predominant cognitive impairment. Genet. Med..

[23] De Michele G., Galatolo D., Barghigiani M., Dello Iacovo D., Trovato R., Tessa A. (2020). Spinocerebellar ataxia type 48: last but not least. Neurol. Sci..

[24] Mengel D., Traschütz A., Reich S., Leyva-Gutiérrez A., Bender F., Hauser S. (2021). A de novo STUB1 variant associated with an early adult-onset multisystemic ataxia phenotype. J. Neurol..

[25] Ye Z., Needham P.G., Estabrooks S.K., Whitaker S.K., Garcia B.L., Misra S. (2017). Symmetry breaking during homodimeric assembly activates an E3 ubiquitin ligase. Sci. Rep..

[26] Zhang M., Windheim M., Roe S.M., Peggie M., Cohen P., Prodromou C. (2005). Chaperoned ubiquitylation--crystal structures of the CHIP U box E3 ubiquitin ligase and a CHIP-Ubc13-Uev1a complex. Mol. Cell..

[27] Meacham G.C., Patterson C., Zhang W., Younger J.M., Cyr D.M. (2001). The Hsc70 co-chaperone CHIP targets immature CFTR for proteasomal degradation. Nat. Cell Biol..

[28] Connell P., Ballinger C.A., Jiang J., Wu Y., Thompson L.J., Höhfeld J. (2001). The co-chaperone CHIP regulates protein triage decisions mediated by heat-shock proteins. Nat. Cell Biol..

[29] McDonough H., Patterson C. (2003). CHIP: a link between the chaperone and proteasome systems. Cell Stress Chaperones.

[30] Ranek M.J., Oeing C., Sanchez-Hodge R., Kokkonen-Simon K.M., Dillard D., Aslam M.I. (2020). CHIP phosphorylation by protein kinase G enhances protein quality control and attenuates cardiac ischemic injury. Nat. Commun..

[31] Dai Q., Zhang C., Wu Y., McDonough H., Whaley R.A., Godfrey V. (2003). CHIP activates HSF1 and confers protection against apoptosis and cellular stress. EMBO J..

[32] De Michele G., Lieto M., Galatolo D., Salvatore E., Cocozza S., Barghigiani M. (2019). Spinocerebellar ataxia 48 presenting with ataxia associated with cognitive, psychiatric, and extrapyramidal features: a report of two Italian families. Parkinsonism Relat. Disord..

[33] Lieto M., Riso V., Galatolo D., De Michele G., Rossi S., Barghigiani M. (2020). The complex phenotype of spinocerebellar ataxia type 48 in eight unrelated Italian families. Eur. J. Neurol..

[34] Palvadeau R., Kaya-Güleç Z.E., Şimşir G., Vural A., Öztop-Çakmak Ö., Genç G. (2020). Cerebellar cognitive-affective syndrome preceding ataxia associated with complex extrapyramidal features in a Turkish SCA48 family. Neurogenetics.

[35] Chen D.-H., Latimer C., Yagi M., Ndugga-Kabuye M.K., Heigham E., Jayadev S. (2020). Heterozygous STUB1 missense variants cause ataxia, cognitive decline, and STUB1 mislocalization. Neurol. Genet..

[36] Mol M.O., van Rooij J.G.J., Brusse E., Verkerk A.J.M.H., Melhem S., den Dunnen W.F.A. (2020). Clinical and pathologic phenotype of a large family with heterozygous STUB1 mutation. Neurol. Genet..

[37] Pakdaman Y., Berland S., Bustad H.J., Erdal S., Thompson B.A., James P.A. (2021). Genetic dominant variants in STUB1, segregating in families with SCA48, display in vitro functional impairments indistinctive from recessive variants associated with SCAR16. Int. J. Mol. Sci..

[38] Dikshit P., Jana N.R. (2007). The co-chaperone CHIP is induced in various stresses and confers protection to cells. Biochem. Biophys. Res. Commun..

[39] Ravel J.-M., Benkirane M., Calmels N., Marelli C., Ory-Magne F., Ewenczyk C. (2021). Expanding the clinical spectrum of STIP1 homology and U-box containing protein 1-associated ataxia. J. Neurol..

[40] van Prooije T.H., Pennings M., Dorresteijn L., Gardeitchik T., Odekerken V.J.J., Oosterloo M. (2024). A new case series suggests that SCA48 (ATX/STUB1) is primarily a monogenic disorder. Mov. Disord..

[41] Shi D., Bai Q., Zhou S., Liu X., Liu H., Yao X. (2018). Molecular dynamics simulation, binding free energy calculation and unbinding pathway analysis on selectivity difference between FKBP51 and FKBP52: insight into the molecular mechanism of isoform selectivity. Proteins.

[42] Barbier M., Davoine C.-S., Petit E., Porché M., Guillot-Noel L., Sayah S. (2023). Intermediate repeat expansions of TBP and STUB1: genetic modifier or pure digenic inheritance in spinocerebellar ataxias?. Genet. Med..

[43] Xu Z., Devlin K.I., Ford M.G., Nix J.C., Qin J., Misra S. (2006). Structure and interactions of the helical and U-box domains of CHIP, the C terminus of HSP70 interacting protein. Biochemistry.

[44] Nikolay R., Wiederkehr T., Rist W., Kramer G., Mayer M.P., Bukau B. (2004). Dimerization of the human E3 ligase CHIP via a coiled-coil domain is essential for its activity. J. Biol. Chem..

[45] Min J.-N., Whaley R.A., Sharpless N.E., Lockyer P., Portbury A.L., Patterson C. (2008). CHIP deficiency decreases longevity, with accelerated aging phenotypes accompanied by altered protein quality control. Mol. Cell. Biol..

[46] Buijsen R.A.M., Toonen L.J.A., Gardiner S.L., van Roon-Mom W.M.C. (2019). Genetics, mechanisms, and therapeutic progress in polyglutamine spinocerebellar ataxias. Neurotherapeutics.

[47] Bettencourt C., Lima M. (2011). Machado-Joseph Disease: from first descriptions to new perspectives. Orphanet J. Rare Dis..

[48] Pulst S.-M. (2006). Genetic Instabilities and Neurological Diseases.

[49] Opie-Martin S., Iacoangeli A., Topp S.D., Abel O., Mayl K., Mehta P.R. (2022). The SOD1-mediated ALS phenotype shows a decoupling between age of symptom onset and disease duration. Nat. Commun..

[50] Pal S., Tiwari A., Sharma K., Sharma S.K. (2020). Does conserved domain SOD1 mutation has any role in ALS severity and therapeutic outcome?. BMC Neurosci..

[51] Huang M., Liu Y.U., Yao X., Qin D., Su H. (2024). Variability in SOD1-associated amyotrophic lateral sclerosis: geographic patterns, clinical heterogeneity, molecular alterations, and therapeutic implications. Transl. Neurodegener..

[52] Nielsen J.E., Johnsen B., Koefoed P., Scheuer K.H., Grønbech-Jensen M., Law I. (2004). Hereditary spastic paraplegia with cerebellar ataxia: a complex phenotype associated with a new SPG4 gene mutation. Eur. J. Neurol..

[53] Solowska J.M., Rao A.N., Baas P.W. (2017). Truncating mutations of SPAST associated with hereditary spastic paraplegia indicate greater accumulation and toxicity of the M1 isoform of spastin. Mol. Biol. Cell..

[54] Shoukier M., Neesen J., Sauter S.M., Argyriou L., Doerwald N., Pantakani D.V.K. (2009). Expansion of mutation spectrum, determination of mutation cluster regions and predictive structural classification of SPAST mutations in hereditary spastic paraplegia. Eur. J. Hum. Genet..

[55] Chelban V., Tucci A., Lynch D.S., Polke J.M., Santos L., Jonvik H. (2017). Truncating mutations in SPAST patients are associated with a high rate of psychiatric comorbidities in hereditary spastic paraplegia. J. Neurol. Neurosurg. Psychiatr..

[56] Yao L., Cao Y., Zhang C., Huang X., Tian W., Cao L. (2024). Clinical and genetic characteristics in a Chinese cohort of complex spastic paraplegia type 4. Clin. Genet..

[57] Kadowaki H., Nishitoh H. (2013). Signaling pathways from the endoplasmic reticulum and their roles in disease. Genes.

[58] Kim P. (2024). Understanding the Unfolded Protein Response (UPR) pathway: insights into neuropsychiatric disorders and therapeutic potentials. Biomol. Ther. (Seoul)..

[59] Capponi S., Geuens T., Geroldi A., Origone P., Verdiani S., Cichero E. (2016). Molecular chaperones in the pathogenesis of amyotrophic lateral sclerosis: the role of HSPB1. Hum. Mutat..

[60] Seki S., Kitaoka Y., Kawata S., Nishiura A., Uchihashi T., Hiraoka S.-I. (2023). Characteristics of sensory neuron dysfunction in amyotrophic lateral sclerosis (ALS): potential for ALS therapy. Biomedicines.

[61] Algon A.L., Ponger P., Daniel L., De Picciotto Y., Gazit E., Brozgol M. (2025). Scale for the assessment and rating of ataxia: a live e-version. J. Neurol..

[62] Petit E., Schmitz-Hübsch T., Coarelli G., Jacobi H., Heinzmann A., Figueroa K.P. (2024). SARA captures disparate progression and responsiveness in spinocerebellar ataxias. J. Neurol..

[63] Jeong Y., Oh A.-R., Jung Y.H., Gi H., Kim Y.U., Kim K. (2023). Targeting E3 ubiquitin ligases and their adaptors as a therapeutic strategy for metabolic diseases. Exp. Mol. Med..

[64] Da Silva J.D., Teixeira-Castro A., Maciel P. (2019). From pathogenesis to novel therapeutics for spinocerebellar ataxia type 3: evading potholes on the way to translation. Neurotherapeutics.

[65] Moore L.R., Rajpal G., Dillingham I.T., Qutob M., Blumenstein K.G., Gattis D. (2017). Evaluation of antisense oligonucleotides targeting ATXN3 in SCA3 mouse models. Mol. Ther. Nucleic Acids.

[66] Thapa P., Chikale R.V., Szulc N.A., Pandrea M.-T., Sztyler A., Jaggi K. (2024). HSP70 inhibits CHIP E3 ligase activity to maintain germline function in Caenorhabditis elegans. J. Biol. Chem..

[67] Thomas B.A.I., Lewis H.L., Jones D.H., Ward S.E. (2023). Central nervous system targeted protein degraders. Biomolecules.

[68] Tsekrekou M., Giannakou M., Papanikolopoulou K., Skretas G. (2024). Protein aggregation and therapeutic strategies in SOD1- and TDP-43- linked ALS. Front. Mol. Biosci..

[69] Wang L., Sooram B., Kumar R., Schedin-Weiss S., Tjernberg L.O., Winblad B. (2025). Tau degradation in Alzheimer’s disease: mechanisms and therapeutic opportunities. Alzheimers Dement.

[70] Zhang H., Amick J., Chakravarti R., Santarriaga S., Schlanger S., McGlone C. (2015). A bipartite interaction between Hsp70 and CHIP regulates ubiquitination of chaperoned client proteins. Structure.

[71] Manage M.M., Nix J.C., Page R.C. (2025). Functional and structural analyses of UbcH5 mutants with enhanced binding to the E3 ubiquitin ligase CHIP. Biochem. Biophys. Res. Commun..

[72] Ronnebaum S.M., Wu Y., McDonough H., Patterson C. (2013). The ubiquitin ligase CHIP prevents SirT6 degradation through noncanonical ubiquitination. Mol. Cell. Biol..

[73] ENCODE Project Consortium (2012). An integrated encyclopedia of DNA elements in the human genome. Nature.

[74] Kagda M.S., Lam B., Litton C., Small C., Sloan C.A., Spragins E. (2025). Data navigation on the ENCODE portal. Nat. Commun..

[75] Luo Y., Hitz B.C., Gabdank I., Hilton J.A., Kagda M.S., Lam B. (2020). New developments on the Encyclopedia of DNA Elements (ENCODE) data portal. Nucleic Acids Res..

[76] Zou Z., Ohta T., Oki S. (2024). ChIP-Atlas 3.0: a data-mining suite to explore chromosome architecture together with large-scale regulome data. Nucleic Acids Res..

[77] Zou Z., Ohta T., Miura F., Oki S. (2022). ChIP-Atlas 2021 update: a data-mining suite for exploring epigenomic landscapes by fully integrating ChIP-seq, ATAC-seq and Bisulfite-seq data. Nucleic Acids Res..

[78] Oki S., Ohta T., Shioi G., Hatanaka H., Ogasawara O., Okuda Y. (2018). ChIP-Atlas: a data-mining suite powered by full integration of public ChIP-seq data. EMBO Rep..

[79] Kovács D., Sigmond T., Hotzi B., Bohár B., Fazekas D., Deák V. (2019). Hsf1base: a comprehensive database of HSF1 (heat shock factor 1) target genes. Int. J. Mol. Sci..

[80] Mendillo M.L., Santagata S., Koeva M., Bell G.W., Hu R., Tamimi R.M. (2012). HSF1 drives a transcriptional program distinct from heat shock to support highly malignant human cancers. Cell.

[81] Kanack A.J., Newsom O.J., Scaglione K.M. (2018). Most mutations that cause spinocerebellar ataxia autosomal recessive type 16 (SCAR16) destabilize the protein quality-control E3 ligase CHIP. J. Biol. Chem..

