# Supplementary material for: Domain-dependent uncoupling of the co-chaperone and E3 ubiquitin ligase CHIP underlies heterogeneity in spinocerebellar ataxia 48
Source: J Biol Chem. 2026 Aug 7;302(10):113399. doi: 10.1016/j.jbc.2026.113399 (PMC13570301; doi:10.1016/j.jbc.2026.113399)
Supplement: Supplementary file 1 [file mmc1.docx]

# SUPPLEMENTAL INFORMATION

Domain-dependent uncoupling of the co-chaperone and E3 ubiquitin ligase CHIP underlies heterogeneity in spinocerebellar ataxia 48

Selin Altinok, Ethan Paulakonis, Michael F Almeida, Isaac Hwang, Rebekah Sanchez-Hodge, Christina D'Ovidio, Alison S Eidman, Shreesti Shrestha, Georgia Roper, Grant Irons, Elena Vargas, Ivy Peng, Morcos Saeed, Aliyaa Pathan, Mel N Azin, Joseph Latham, Sarah M Ronnebaum, Chang-he Shi, Mark J. Ranek, K Matthew Scaglione, Richard C Page, Nicholas G Brown, and Jonathan C Schisler

Manuscript JBC-D-26-00078

Correspondence: nbrown1@email.unc.edu; schisler@unc.edu

TABLE OF CONTENTS

[SUPPLEMENTAL INFORMATION	1](#_Toc234916869)

[SUPPLEMENTAL TABLES	3](#_Toc234916870)

[Supplementary Table 1. Statistical comparisons across all figure panels.	3](#_Toc234916871)

[Supplementary Table 2. Biochemical Profiles of CHIP mutants	7](#_Toc234916872)

[Supplementary Table 3. SCA48 Patient Genotype/Phenotype	8](#_Toc234916873)

[Supplementary Table 4. Primers used in CHIP Mutagenesis.	10](#_Toc234916874)

[Supplementary Table 5. Primary antibodies used for immunoblotting and immunofluorescence.	11](#_Toc234916875)

[Supplementary Table 6. Full unfiltered g:Profiler multiquery enrichment results (JBC-D-26-00078_Supplemental_Table_6.csv)	12](#_Toc234916876)

[SUPPLEMENTAL METHODS	13](#_Toc234916877)

[Nuclear and Cytoplasmic Fractionation	13](#_Toc234916878)

[SUPPLEMENTAL FIGURE LEGENDS	13](#_Toc234916879)

[Supplementary Figure 1. Structural modeling of CHIP variants reveals steric clashes at multiple mutation sites.	13](#_Toc234916880)

[Supplementary Figure 2. Mass photometry reveals distinct oligomerization profiles for CHIP wild-type and SCA48-associated variants.	13](#_Toc234916881)

[Supplementary Figure 3. Oligomerization profiles of CHIP at different concentrations.	14](#_Toc234916882)

[Supplementary Figure 4. Binding affinity and function of UBE2D2 with CHIP variants.	14](#_Toc234916883)

[Supplementary Figure 5. Impact of SCA48 mutations on CHIP phosphorylation, vector control immunofluorescence, and subcellular localization of CHIP variants and HSF1 determined by fractionated immunoblotting.	15](#_Toc234916884)

[Supplementary Figure 6. Validation of *STUB1* knockdown, HSF1 activation, and RNA-seq quality control.	15](#_Toc234916885)

[Supplementary Figure 7. Relationship between CHIP mutation domain and clinical severity in SCA48 patients.	16](#_Toc234916886)

# SUPPLEMENTAL TABLES

## Supplementary Table 1. Statistical comparisons across all figure panels.

| **Figure** | **Panel** | **Comparison** | **Test** | **Test statistic** | **Exact p** | **Adj. p** | **n** | **Notes** |
| --- | --- | --- | --- | --- | --- | --- | --- | --- |
| Fig 1 | D | G33S vs WT | Mixed-effects model + Dunnett's vs WT | t = -0.66 | — | 0.9996 | 3 | log₂ FC CHIP protein (COS-7) |
| Fig 1 | D | F37L vs WT | Mixed-effects model + Dunnett's vs WT | t = 2.27 | — | 0.2692 | 3 | log₂ FC CHIP protein (COS-7) |
| Fig 1 | D | I53T vs WT | Mixed-effects model + Dunnett's vs WT | t = -0.99 | — | 0.9824 | 3 | log₂ FC CHIP protein (COS-7) |
| Fig 1 | D | P57L vs WT | Mixed-effects model + Dunnett's vs WT | t = -2.48 | — | 0.1794 | 3 | log₂ FC CHIP protein (COS-7) |
| Fig 1 | D | A67T vs WT | Mixed-effects model + Dunnett's vs WT | t = -2.32 | — | 0.2469 | 3 | log₂ FC CHIP protein (COS-7) |
| Fig 1 | D | R225* vs WT | Mixed-effects model + Dunnett's vs WT | t = 0.96 | — | 0.9863 | 3 | log₂ FC CHIP protein (COS-7) |
| Fig 1 | D | P228S vs WT | Mixed-effects model + Dunnett's vs WT | t = -0.97 | — | 0.9851 | 3 | log₂ FC CHIP protein (COS-7) |
| Fig 1 | D | Y230Cfs*9 vs WT | Mixed-effects model + Dunnett's vs WT | t = -3.53 | — | 0.0159 | 3 | log₂ FC CHIP protein (COS-7) |
| Fig 1 | D | R241W vs WT | Mixed-effects model + Dunnett's vs WT | t = -0.42 | — | 1 | 3 | log₂ FC CHIP protein (COS-7) |
| Fig 1 | D | C244Yfs*24 vs WT | Mixed-effects model + Dunnett's vs WT | t = 0.03 | — | 1 | 3 | log₂ FC CHIP protein (COS-7) |
| Fig 1 | D | V264Gfs*4 vs WT | Mixed-effects model + Dunnett's vs WT | t = 2.44 | — | 0.194 | 3 | log₂ FC CHIP protein (COS-7) |
| Fig 1 | D | P274Afs*3 vs WT | Mixed-effects model + Dunnett's vs WT | t = 3.63 | — | 0.0125 | 3 | log₂ FC CHIP protein (COS-7) |
| Fig 1 | D | L275Dfs*16 vs WT | Mixed-effects model + Dunnett's vs WT | t = 6.52 | — | <0.0001 | 3 | log₂ FC CHIP protein (COS-7) |
| Fig 1 | D | ΔStartCodon vs WT | Mixed-effects model + Dunnett's vs WT | t = -4.66 | — | 0.0008 | 3 | log₂ FC CHIP protein (COS-7) |
| Fig 2 | B | Oligomeric-state proportions, by variant | n/a (descriptive) | n/a | n/a | n/a | 3 | Mean ± SD reported only |
| Fig 2 | C | PC1 by MP profile (overall ANOVA) | One-way ANOVA | F(2,11) = 43 | <0.0001 | n/a | 3 | Reported in legend |
| Fig 2 | C | PC2 by MP profile (overall ANOVA) | One-way ANOVA | F(2,11) = 5.7 | 0.0201 | n/a | 3 | Reported in legend |
| Fig 2 | C | PC1 Tukey: D vs DR | Tukey HSD | Δ = -1.636773, SE = 0.5413156 | — | 0.0288 | 3 | All pairwise comparisons |
| Fig 2 | C | PC2 Tukey: D vs DR | Tukey HSD | Δ = -1.987195, SE = 0.6968198 | — | 0.0386 | 3 |  |
| Fig 2 | C | PC1 Tukey: D vs HMW | Tukey HSD | Δ = -2.022836, SE = 0.5648620 | — | 0.011 | 3 | All pairwise comparisons |
| Fig 2 | C | PC2 Tukey: D vs HMW | Tukey HSD | Δ = -2.420298, SE = 0.7271304 | — | 0.017 | 3 |  |
| Fig 2 | C | PC1 Tukey: DR vs HMW | Tukey HSD | Δ = 3.659608, SE = 0.3953210 | — | <0.0001 | 3 | All pairwise comparisons |
| Fig 2 | C | PC2 Tukey: DR vs HMW | Tukey HSD | Δ = 0.433103, SE = 0.5088853 | — | 0.6805 | 3 |  |
| Fig 3 | B | Overall ANOVA (HSP70 ubiquitination) | One-way ANOVA | F(13, 49) = 26.69 | <0.0001 | n/a | 4–11 | R² = 0.876. Brown-Forsythe (unequal variances): F = 2.111, p = 0.0303 |
| Fig 3 | B | G33S vs WT (HSP70 Ub) | Dunnett's vs WT | q = 8.978, DF = 49 | — | <0.0001 | 4–11 | HSP70 ubiquitination |
| Fig 3 | B | F37L vs WT (HSP70 Ub) | Dunnett's vs WT | q = 9.746, DF = 49 | — | <0.0001 | 4–11 | HSP70 ubiquitination |
| Fig 3 | B | I53T vs WT (HSP70 Ub) | Dunnett's vs WT | q = 7.119, DF = 49 | — | <0.0001 | 4–11 | HSP70 ubiquitination |
| Fig 3 | B | P57L vs WT (HSP70 Ub) | Dunnett's vs WT | q = 9.676, DF = 49 | — | <0.0001 | 4–11 | HSP70 ubiquitination |
| Fig 3 | B | A67T vs WT (HSP70 Ub) | Dunnett's vs WT | q = 1.465, DF = 49 | — | 0.8076 | 4–11 | HSP70 ubiquitination |
| Fig 3 | B | R225* vs WT (HSP70 Ub) | Dunnett's vs WT | q = 9.895, DF = 49 | — | <0.0001 | 4–11 | HSP70 ubiquitination |
| Fig 3 | B | P228S vs WT (HSP70 Ub) | Dunnett's vs WT | q = 9.296, DF = 49 | — | <0.0001 | 4–11 | HSP70 ubiquitination |
| Fig 3 | B | Y230Cfs*9 vs WT (HSP70 Ub) | Dunnett's vs WT | q = 9.951, DF = 49 | — | <0.0001 | 4–11 | HSP70 ubiquitination |
| Fig 3 | B | R241W vs WT (HSP70 Ub) | Dunnett's vs WT | q = 2.915, DF = 49 | — | 0.0599 | 4–11 | HSP70 ubiquitination |
| Fig 3 | B | C244Yfs*24 vs WT (HSP70 Ub) | Dunnett's vs WT | q = 9.775, DF = 49 | — | <0.0001 | 4–11 | HSP70 ubiquitination |
| Fig 3 | B | V264Gfs*4 vs WT (HSP70 Ub) | Dunnett's vs WT | q = 9.776, DF = 49 | — | <0.0001 | 4–11 | HSP70 ubiquitination |
| Fig 3 | B | P274Afs*3 vs WT (HSP70 Ub) | Dunnett's vs WT | q = 9.767, DF = 49 | — | <0.0001 | 4–11 | HSP70 ubiquitination |
| Fig 3 | B | L275Dfs*16 vs WT (HSP70 Ub) | Dunnett's vs WT | q = 9.926, DF = 49 | — | <0.0001 | 4–11 | HSP70 ubiquitination |
| Fig 3 | D | Overall ANOVA (E2~Ub / autoubiquitination) | One-way ANOVA | F(13, 28) = 67 | <0.0001 | n/a | 3 | R² = 0.969. Brown-Forsythe: F = 0.7829, p = 0.6711 (variances equal) |
| Fig 3 | D | G33S vs WT (autoub) | Dunnett's vs WT | q = 0.133, DF = 28 | — | >0.9999 | 3 | E2~Ub depletion as readout |
| Fig 3 | D | F37L vs WT (autoub) | Dunnett's vs WT | q = 0.215, DF = 28 | — | >0.9999 | 3 | E2~Ub depletion as readout |
| Fig 3 | D | I53T vs WT (autoub) | Dunnett's vs WT | q = 1.550, DF = 28 | — | 0.637 | 3 | E2~Ub depletion as readout |
| Fig 3 | D | P57L vs WT (autoub) | Dunnett's vs WT | q = 0.569, DF = 28 | — | 0.9995 | 3 | E2~Ub depletion as readout |
| Fig 3 | D | A67T vs WT (autoub) | Dunnett's vs WT | q = 0.489, DF = 28 | — | >0.9999 | 3 | E2~Ub depletion as readout |
| Fig 3 | D | R225* vs WT (autoub) | Dunnett's vs WT | q = 12.27, DF = 28 | — | <0.0001 | 3 | E2~Ub depletion as readout |
| Fig 3 | D | P228S vs WT (autoub) | Dunnett's vs WT | q = 10.83, DF = 28 | — | <0.0001 | 3 | E2~Ub depletion as readout |
| Fig 3 | D | Y230Cfs*9 vs WT (autoub) | Dunnett's vs WT | q = 12.90, DF = 28 | — | <0.0001 | 3 | E2~Ub depletion as readout |
| Fig 3 | D | R241W vs WT (autoub) | Dunnett's vs WT | q = 6.177, DF = 28 | — | <0.0001 | 3 | E2~Ub depletion as readout |
| Fig 3 | D | C244Yfs*24 vs WT (autoub) | Dunnett's vs WT | q = 11.98, DF = 28 | — | <0.0001 | 3 | E2~Ub depletion as readout |
| Fig 3 | D | V264Gfs*4 vs WT (autoub) | Dunnett's vs WT | q = 12.68, DF = 28 | — | <0.0001 | 3 | E2~Ub depletion as readout |
| Fig 3 | D | P274Afs*3 vs WT (autoub) | Dunnett's vs WT | q = 11.69, DF = 28 | — | <0.0001 | 3 | E2~Ub depletion as readout |
| Fig 3 | D | L275Dfs*16 vs WT (autoub) | Dunnett's vs WT | q = 9.793, DF = 28 | — | <0.0001 | 3 | E2~Ub depletion as readout |
| Fig 4 | B | Interaction (HSC70/HSP40 × CHIP) — overall | Two-way mixed ANOVA | F(14, 270) = 47.99 | <0.0001 | n/a | 11 | Main effects: Condition F(14,270)=64.24 p<0.0001; HSC70/HSP40 F(1,270)=758.51 p<0.0001 |
| Fig 4 | B | WT: +HSC70/HSP40 vs −HSC70/HSP40 | Tukey HSD | t = -22.17, DF = 270 | — | <0.0001 | 11 | Refolding activity |
| Fig 4 | B | G33S: +HSC70/HSP40 vs −HSC70/HSP40 | Tukey HSD | t = -0.64, DF = 270 | — | >0.9999 | 11 | Refolding activity |
| Fig 4 | B | F37L: +HSC70/HSP40 vs −HSC70/HSP40 | Tukey HSD | t = -0.09, DF = 270 | — | >0.9999 | 11 | Refolding activity |
| Fig 4 | B | I53T: +HSC70/HSP40 vs −HSC70/HSP40 | Tukey HSD | t = -0.75, DF = 270 | — | >0.9999 | 11 | Refolding activity |
| Fig 4 | B | P57L: +HSC70/HSP40 vs −HSC70/HSP40 | Tukey HSD | t = -3.44, DF = 270 | — | 0.1425 | 11 | Refolding activity |
| Fig 4 | B | A67T: +HSC70/HSP40 vs −HSC70/HSP40 | Tukey HSD | t = -2.07, DF = 270 | — | 0.9652 | 11 | Refolding activity |
| Fig 4 | B | R225*: +HSC70/HSP40 vs −HSC70/HSP40 | Tukey HSD | t = -13.40, DF = 270 | — | <0.0001 | 11 | Refolding activity |
| Fig 4 | B | P228S: +HSC70/HSP40 vs −HSC70/HSP40 | Tukey HSD | t = -5.87, DF = 270 | — | <0.0001 | 11 | Refolding activity |
| Fig 4 | B | Y230Cfs*9: +HSC70/HSP40 vs −HSC70/HSP40 | Tukey HSD | t = -17.09, DF = 270 | — | <0.0001 | 11 | Refolding activity |
| Fig 4 | B | R241W: +HSC70/HSP40 vs −HSC70/HSP40 | Tukey HSD | t = -4.56, DF = 270 | — | 0.0028 | 11 | Refolding activity |
| Fig 4 | B | C244Yfs*24: +HSC70/HSP40 vs −HSC70/HSP40 | Tukey HSD | t = -3.35, DF = 270 | — | 0.1785 | 11 | Refolding activity |
| Fig 4 | B | V264Gfs*4: +HSC70/HSP40 vs −HSC70/HSP40 | Tukey HSD | t = -6.90, DF = 270 | — | <0.0001 | 11 | Refolding activity |
| Fig 4 | B | P274Afs*3: +HSC70/HSP40 vs −HSC70/HSP40 | Tukey HSD | t = -12.42, DF = 270 | — | <0.0001 | 11 | Refolding activity |
| Fig 4 | B | L275Dfs*16: +HSC70/HSP40 vs −HSC70/HSP40 | Tukey HSD | t = -16.17, DF = 270 | — | <0.0001 | 11 | Refolding activity |
| Fig 5 | A | PC1 by domain (TPR vs U-box) | Two-sample t-test | t(11) = 3.3 | 0.0069 | n/a | 13 | Reported in legend |
| Fig 5 | B | MP profile × domain (contingency) | Fisher's exact | n/a | 0.0054 | n/a | 13 | Reported in legend |
| Fig 6 | B | Overall Dunnett's (CHX chase half-life) | Mixed-design log-linear regression | Time × variant interaction tests, Holm-corrected | — | — | 3 | n=3 biological reps with technical duplicates |
| Fig 6 | B | G33S vs WT (CHX t½) | Mixed-design log-linear regression | β = -0.234, t = -2.494, DF = 60 | 0.0126 | 0.038 | 3 | Time × G33S interaction; t½ = 1.8 h (95% CI 1.2–2.4) |
| Fig 6 | B | P228S vs WT (CHX t½) | Mixed-design log-linear regression | β = -0.214, t = -2.122, DF = 60 | 0.0338 | 0.068 | 3 | Time × P228S interaction; t½ = 1.9 h (95% CI 1.1–2.7); trending |
| Fig 6 | B | L275Dfs*16 vs WT (CHX t½) | Mixed-design log-linear regression | β = -0.042, t = -0.450, DF = 60 | 0.6525 | 0.6525 | 3 | Time × L275Dfs interaction; t½ = 3.7 h (95% CI 1.1–6.2); ns |
| Fig 6 | D | G33S pS19 vs hypothetical 1.0 | One-sample t-test | t = 0.776, df = 4 | 0.4809 | n/a (uncorrected) | 5 | Mean = 0.858; 95% CI −0.650 to 0.366 |
| Fig 6 | D | P228S pS19 vs hypothetical 1.0 | One-sample t-test | t = 2.835, df = 4 | 0.0471 | n/a (uncorrected) | 5 | Mean = 0.542; 95% CI −0.907 to −0.009 * |
| Fig 6 | D | L275Dfs*16 pS19 vs hypothetical 1.0 | One-sample t-test | t = 1.091, df = 4 | 0.3366 | n/a (uncorrected) | 5 | Mean = 1.292; 95% CI −0.452 to 1.036 |
| Fig 6 | D | WT pS19 vs hypothetical 1.0 | One-sample t-test | t = 0.944, df = 4 | 0.3987 | n/a | 5 | Mean = 1.308; 95% CI −0.598 to 1.214 |
| Fig 6 | E | Temperature × CHIP interaction (HSF1 reporter) | Two-way mixed ANOVA | F(4, 78) = 11.67 | <0.0001 | n/a | 9 | Main effects: Heat Shock F(1,78)=545.55 p<0.0001; condition F(4,78)=13.32 p<0.0001 |
| Fig 6 | E | vector at 42°C vs WT at 42°C | Tukey HSD | t = -7.48, DF = 78 | — | <0.0001 | 9 | Mutant vs WT at heat shock |
| Fig 6 | E | WT: 42°C vs 37°C | Tukey HSD | t = -12.22, DF = 78 | — | <0.0001 | 9 | Within-condition temp effect |
| Fig 6 | E | G33S at 42°C vs WT at 42°C | Tukey HSD | t = +1.77, DF = 78 | — | 0.749 | 9 | Mutant vs WT at heat shock |
| Fig 6 | E | G33S: 42°C vs 37°C | Tukey HSD | t = -10.44, DF = 78 | — | <0.0001 | 9 | Within-condition temp effect |
| Fig 6 | E | P228S at 42°C vs WT at 42°C | Tukey HSD | t = +1.13, DF = 78 | — | 0.9801 | 9 | Mutant vs WT at heat shock |
| Fig 6 | E | P228S: 42°C vs 37°C | Tukey HSD | t = -10.82, DF = 78 | — | <0.0001 | 9 | Within-condition temp effect |
| Fig 6 | E | L275Dfs*16 at 42°C vs WT at 42°C | Tukey HSD | t = -1.94, DF = 78 | — | 0.6444 | 9 | Mutant vs WT at heat shock |
| Fig 6 | E | L275Dfs*16: 42°C vs 37°C | Tukey HSD | t = -13.91, DF = 78 | — | <0.0001 | 9 | Within-condition temp effect |
| Fig 6 | H | Temp main effect (nuclear CHIP) | Two-way ANOVA | F(1, 140) = 49.57 | <0.0001 | — | 30 |  |
| Fig 6 | H | CHIP variant main effect (nuclear CHIP) | Two-way ANOVA | F(4, 140) = 85.93 | <0.0001 | — | 30 |  |
| Fig 6 | H | Temp × variant interaction (nuclear CHIP) | Two-way ANOVA | F(4, 140) = 49.21 | <0.0001 | — | 30 |  |
| Fig 6 | H | WT 37°C vs 42°C (nuclear CHIP) | Tukey HSD | t = -15.62, DF = 140 | <0.0001 | — | 30 | WT translocates strongly with heat shock; ‡ |
| Fig 6 | H | G33S 37°C vs 42°C (nuclear CHIP) | Tukey HSD | t = -0.20, DF = 140 | 1 | — | 30 | ns — mutants fail to translocate |
| Fig 6 | H | G33S vs WT at 37°C (nuclear CHIP) | Tukey HSD | t = -5.75, DF = 140 | <0.0001 | — | 30 | Elevated basal nuclear CHIP |
| Fig 6 | H | G33S vs WT at 42°C (nuclear CHIP) | Tukey HSD | t = +9.67, DF = 140 | <0.0001 | — | 30 | WT >> mutant; mutant fails to translocate |
| Fig 6 | H | P228S 37°C vs 42°C (nuclear CHIP) | Tukey HSD | t = +1.15, DF = 140 | 0.9789 | — | 30 | ns — fails to translocate |
| Fig 6 | H | P228S vs WT at 37°C (nuclear CHIP) | Tukey HSD | t = -4.41, DF = 140 | 0.0008 | — | 30 | Elevated basal nuclear CHIP |
| Fig 6 | H | P228S vs WT at 42°C (nuclear CHIP) | Tukey HSD | t = +12.35, DF = 140 | <0.0001 | — | 30 | WT >> mutant |
| Fig 6 | H | L275Dfs*16 37°C vs 42°C (nuclear CHIP) | Tukey HSD | t = -1.08, DF = 140 | 0.9863 | — | 30 | ns — fails to translocate |
| Fig 6 | H | L275Dfs*16 vs WT at 37°C (nuclear CHIP) | Tukey HSD | t = -7.18, DF = 140 | <0.0001 | — | 30 | Elevated basal nuclear CHIP |
| Fig 6 | H | L275Dfs*16 vs WT at 42°C (nuclear CHIP) | Tukey HSD | t = +7.36, DF = 140 | <0.0001 | — | 30 | WT >> mutant |
| Fig 6 | I | Temp main effect (nuclear HSF1) | Two-way ANOVA | F(1, 140) = 182.64 | <0.0001 | — | 30 |  |
| Fig 6 | I | CHIP variant main effect (nuclear HSF1) | Two-way ANOVA | F(4, 140) = 43.94 | <0.0001 | — | 30 |  |
| Fig 6 | I | Temp × variant interaction (nuclear HSF1) | Two-way ANOVA | F(4, 140) = 4.24 | 0.0028 | — | 30 |  |
| Fig 6 | I | WT 37°C vs 42°C (nuclear HSF1) | Tukey HSD | t = -7.24, DF = 140 | <0.0001 | — | 30 | HSF1 translocates with heat shock; ‡ |
| Fig 6 | I | G33S 37°C vs 42°C (nuclear HSF1) | Tukey HSD | t = -5.55, DF = 140 | <0.0001 | — | 30 | HSF1 translocates normally |
| Fig 6 | I | G33S vs WT at 37°C (nuclear HSF1) | Tukey HSD | t = -1.63, DF = 140 | 0.8295 | — | 30 | ns |
| Fig 6 | I | G33S vs WT at 42°C (nuclear HSF1) | Tukey HSD | t = +0.05, DF = 140 | 1 | — | 30 | ns |
| Fig 6 | I | P228S 37°C vs 42°C (nuclear HSF1) | Tukey HSD | t = -8.87, DF = 140 | <0.0001 | — | 30 | HSF1 translocates |
| Fig 6 | I | P228S vs WT at 37°C (nuclear HSF1) | Tukey HSD | t = -2.70, DF = 140 | 0.1824 | — | 30 | ns |
| Fig 6 | I | P228S vs WT at 42°C (nuclear HSF1) | Tukey HSD | t = -4.34, DF = 140 | 0.0011 | — | 30 | P228S > WT — gain of function |
| Fig 6 | I | L275Dfs*16 37°C vs 42°C (nuclear HSF1) | Tukey HSD | t = -5.01, DF = 140 | <0.0001 | — | 30 | HSF1 translocates |
| Fig 6 | I | L275Dfs*16 vs WT at 37°C (nuclear HSF1) | Tukey HSD | t = -7.39, DF = 140 | <0.0001 | — | 30 | L275Dfs elevates basal nuclear HSF1 |
| Fig 6 | I | L275Dfs*16 vs WT at 42°C (nuclear HSF1) | Tukey HSD | t = -5.16, DF = 140 | <0.0001 | — | 30 | L275Dfs > WT — gain of function |
| Fig 8 | A | AOO normality | Shapiro-Wilk | W = 0.9863 | 0.5733 | n/a | 78 | Mean=47.92, SD=15.05, median=48; AOO consistent with normal distribution |
| Fig 8 | B | Sex F:M vs 1:1 (goodness-of-fit) | Chi-squared (Pearson) | χ²(1) = 8.667 | 0.0032 | n/a | 78 | F=52 (67%), M=26 (33%); ~2:1 female predominance; LR χ²=8.835, p=0.0030 |
| Fig 8 | C | Mutations per exon (frequency) | Chi-squared (Pearson) | χ²(6) = 38.0337 | <0.0001 | n/a | — |  |
| Fig 8 | D | Exon 1 expected vs observed | Multinomial / binomial | Expected vs observed multinomial | 0.0166 | 0.0673 | — | Trending; does not survive Holm correction |
| Fig 8 | D | Exon 3 expected vs observed | Multinomial / binomial | Expected vs observed multinomial | 0.0048 | 0.0335 | — | Survive Holm correction |
| Fig 8 | D | Exon 4 expected vs observed | Multinomial / binomial | Expected vs observed multinomial | 0.0063 | 0.0376 | — | Survive Holm correction |
| Fig 8 | D | Exon 7 expected vs observed | Multinomial / binomial | Expected vs observed multinomial | 0.0116 | 0.0579 | — | Trending; does not survive Holm correction |
| Fig 9 | A | AOO: TPR vs U-box | Two-tailed t-test / F-test | F(1, 71) = 4.70 | 0.0335 | 0.0335 (4 tests via BH; survives FDR<0.05) | 73 | TPR 44.2±16.2 (n~36) vs U-box 51.7±13.1 (n~37); U-box later onset |
| Fig 9 | B/C | UMD × domain | Fisher's exact (2-tailed) | n = 69 (TPR 51% / U-box 21% positive) | 0.023 | <0.05 (BH FDR) | — | Survives BH 5% FDR |
| Fig 9 | B/C | ITR × domain | Fisher's exact (2-tailed) | n = 42 (TPR 78% / U-box 25% positive) | 0.0015 | <0.05 (BH FDR) | — | Survives BH 5% FDR; strongest |
| Fig 9 | B/C | Gait ataxia × domain | Fisher's exact (2-tailed) | n = 74 (TPR 100% / U-box 86% positive) | 0.02 | <0.05 (BH FDR) | — | Survives BH 5% FDR; near-universal |
| Fig 9 | B/C | Dysarthria × domain | Fisher's exact (2-tailed) | n = 73 (TPR 56% / U-box 88% positive) | 0.0039 | <0.05 (BH FDR) | — | Survives BH 5% FDR; U-box predominant |

Per-figure-panel statistical reporting for every quantitative comparison in this study. Columns list the figure number, panel, specific comparison performed, statistical test applied, test statistic with relevant degrees of freedom, unadjusted exact p-value, multiple-comparison-adjusted p-value (where corrections were applied), number of biological replicates (n), and brief contextual notes. Tests include one- and two-way ANOVA, mixed-effects models, mixed-design log-linear regression, t-tests (one- and two-sample, two-tailed unless noted), Tukey HSD and Dunnett's post-hoc procedures, chi-squared goodness-of-fit, Fisher's exact, Shapiro-Wilk normality, and Benjamini–Hochberg false-discovery-rate or Holm–Bonferroni multiple-comparison corrections, as appropriate. "—" denotes a cell not applicable to the given comparison; "n/a" denotes an overall test where no per-pair adjusted value is meaningful (see JBC-D-26-00078_Supplemental_Table_S1.xlsx).

## Supplementary Table 2. Biochemical Profiles of CHIP mutants

| **Mutation** | **AA#** | **Exon** | **Domain** | **T_m_ (C°)** | **%Expression** | **%AutoUb** | **%HSP70Ub** | **%Co-chaper.** | **MP Profile** |
| --- | --- | --- | --- | --- | --- | --- | --- | --- | --- |
| WT | N/A | N/A | N/A | 44.12 | 100 | 100 | 100 | 100 | 1 |
| G33S | 33 | 1 | TPR | 41.40 | 90 | 101 | 10 | 33 | 1 |
| F37L | 37 | 1 | TPR | 44.87 | 281 | 98 | 4 | 27 | 1 |
| I53T | 53 | 1 | TPR | 41.78 | 73 | 84 | 37 | 29 | 1 |
| P57L | 57 | 2 | TPR | 48.18 | 45 | 106 | 5 | 35 | 1 |
| A67T | 67 | 2 | TPR | 40.50 | 62 | 95 | 107 | 30 | 1 |
| R225* | 225 | 6 | Ubox | 47.00 | 163 | 0 | 1 | 101 | 2 |
| P228S | 228 | 6 | Ubox | 42.74 | 75 | 0 | 6 | 68 | 3 |
| R241W | 241 | 6 | Ubox | 40.80 | 118 | 37 | 84 | 55 | 1 |
| Y230Cfs*9 | 230 | 6 | Ubox | 47.29 | 42 | 0 | 1 | 124 | 2 |
| C244Yfs*24 | 244 | 6 | Ubox | 42.48 | 137 | 0 | 2 | 36 | 3 |
| V264Gfs*4 | 264 | 7 | Ubox | 42.79 | 271 | 0 | 2 | 65 | 3 |
| P274Afs*3 | 274 | 7 | Ubox | 45.15 | 412 | 0 | 2 | 92 | 3 |
| L275Dfs*16 | 275 | 7 | Ubox | 44.47 | 954 | 1 | 1 | 120 | 3 |

AA = amino acid, Tm = thermal melting temperature, %Expression = percentage of the steady-state protein levels compared to CHIP WT, %AutoUb = percentage of CHIP protein that was modified by ubiquitination, %HSP70Ub = percentage of HSP70 protein that was modified by ubiquitination, %Co-chaper = percentage of luciferase refolding activity compared to CHIP WT, MP profile = mass photometry profile identified (1 = dimer repeating units, 2 = dimer only, 3 = non-discrete high molecular weight).

## Supplementary Table 3. SCA48 Patient Genotype/Phenotype

| **STUB1** | **CHIP** | **Exon** | **Domain** | **AOO (y)** | **Sex** | **Ataxia** | **Dys** | **SARA** | **CD** | **UMND** | **ITR** | **Nys** | **Citation** |
| --- | --- | --- | --- | --- | --- | --- | --- | --- | --- | --- | --- | --- | --- |
| c.97G>A | G33S | 1 | TPR | 50 | F | Y | Y | N/A | Y | N | N | N/A | De Michele, G |
| c.97G>A | G33S | 1 | TPR | 40 | F | Y | Y | N/A | Y | Y | Y | N/A | De Michele, G |
| c.97G>A | G33S | 1 | TPR | 22 | M | Y | Y | N/A | Y | Y | Y | N/A | De Michele, G |
| c.97G>A | G33S | 1 | TPR | 28 | F | Y | Y | N/A | Y | Y | Y | N/A | De Michele, G |
| c.97G>A | G33S | 1 | TPR | 49 | M | Y | Y | N/A | Y | Y | Y | N/A | De Michele, G |
| c.97G>A | G33S | 1 | TPR | 44 | M | Y | Y | N/A | Y | Y | Y | N/A | De Michele, G |
| c.111C>G | F37L | 1 | TPR | 41 | F | Y | Y | N/A | Y | N | N | Y | Chen, D-H |
| c.134C>T | A45V | 1 | TPR | 62 | F | Y | Y | 28 | Y | Y | Y | N | Ravel, J |
| c.136G>C | A46P | 1 | TPR | 60 | F | Y | N | N/A | N | N | N/A | Y | Roux, T |
| c.136G>C | A46P | 1 | TPR | 38 | F | Y | N | N/A | Y | N | N/A | N | Roux, T |
| c.136G>C | A46P | 1 | TPR | 47 | F | Y | Y | N/A | Y | N | N/A | N | Roux, T |
| c.146A>G | Y49C | 1 | TPR | N/A | F | N/A | N | N/A | N/A | N | N/A | N/A | Roux, T |
| c.146A>G | Y49C | 1 | TPR | 37 | M | Y | Y | 12.5 | Y | N | N/A | N | Roux, T |
| c.146A>G | Y49C | 1 | TPR | 37 | F | Y | Y | 12 | Y | N | N/A | N | Roux, T |
| c.146A>G | Y49C | 1 | TPR | N/A | F | N/A | N/A | N/A | N/A | N/A | N/A | N/A | Roux, T |
| c.146A>G | Y49C | 1 | TPR | 48 | F | Y | N/A | 8 | Y | Y | N | N | Ravel, J |
| c.146A>G | Y49C | 1 | TPR | 84 | F | N/A | N/A | N/A | Y | N/A | N/A | N | Ravel, J |
| c.146A>G | Y49C | 1 | TPR | 53 | F | Y | N | 33 | Y | Y | Y | N | Ravel, J |
| c.158T>C | I53T | 1 | TPR | 41 | M | Y | Y | N/A | Y | Y | Y | N | Chen, D-H |
| c.158T>C | I53T | 1 | TPR | 63 | M | Y | Y | N/A | Y | Y | Y | N | Chen, D-H |
| c.158T>C | I53T | 1 | TPR | 26 | M | Y | Y | N/A | Y | Y | Y | N | Chen, D-H |
| c.158T>C | I53T | 1 | TPR | 24 | F | Y | N | N/A | Y | Y | Y | N | Chen, D-H |
| c.170C>T | P57L | 2 | TPR | 43 | M | Y | Y | 10 | N | Y | Y | N | Lieto, M |
| c.170C>T | P57L | 2 | TPR | 60 | M | Y | N | N/A | Y | Y | N/A | N | Roux, T |
| c.170C>T | P57L | 2 | TPR | 40 | F | Y | Y | N/A | N | N | N/A | N | Roux, T |
| c.170C>T | P57L | 2 | TPR | 40 | F | Y | Y | 4 | N | N | N/A | N | Roux, T |
| c.194A>G | N65S | 2 | TPR | 51 | F | Y | N | N/A | N | N | N/A | Y | Roux, T |
| c.194A>G | N65S | 2 | TPR | 55 | F | Y | N | N/A | Y | N | N/A | N | Roux, T |
| c.194A>G | N65S | 2 | TPR | 30 | F | Y | N | N/A | N | Y | N/A | Y | Roux, T |
| c.194A>G | N65S | 2 | TPR | 41 | F | Y | Y | N/A | Y | N | N/A | N | Roux, T |
| c.197G>C | R66P | 2 | TPR | 32 | M | Y | N/A | 28 | Y | N | N | Y | Ravel, J |
| c.197G>C | R66P | 2 | TPR | 72 | M | Y | N | 24 | N | Y | Y | N | Ravel, J |
| c.199G>A | A67T | 2 | TPR | 37 | F | Y | Y | 17 | N | Y | Y | N | Lieto, M |
| c.206G>A | C69Y | 2 | TPR | 44 | M | Y | N | 7.5 | Y | N | N/A | N | Roux, T |
| c.206G>A | C69Y | 2 | TPR | 74 | F | Y | N | N/A | Y | N | N/A | Y | Roux, T |
| c.221A>C | Q74P | 2 | TPR | 65 | F | Y | Y | N/A | Y | N | N/A | N | Roux, T |
| c.235G>C | A79P | 2 | TPR | 40 | F | Y | Y | N/A | Y | Y | N/A | N | Roux, T |
| c.301G>C | G101R | 2 | TPR | N/A | F | Y | N | 7 | N | N | N/A | N | Roux, T |
| c.301G>C | G101R | 2 | TPR | 25 | M | Y | N | 12 | N | Y | N/A | N | Roux, T |
| c.301G>C | G101R | 2 | TPR | 30 | M | Y | Y | 11.5 | N | N | N/A | Y | Roux, T |
| c.301G>C | G101R | 2 | TPR | 50 | F | Y | N | 11 | Y | Y | N/A | N | Roux, T |
| c.301G>C | G101R | 2 | TPR | N/A | F | N/A | N | 6.5 | N | Y | N/A | N | Roux, T |
| c.358+2T>C | Splicedonor | 2 | TPR | 30 | M | Y | N | N/A | Y | N | N/A | N | Roux, T |
| c.426_428del | K143del | 3 | CC | 52 | F | Y | N | N/A | Y | N | N/A | Y | Roux, T |
| c.426_428del | K143del | 3 | CC | 27 | F | Y | N | 4.5 | N | N | N/A | Y | Roux, T |
| c.426_428del | K143del | 3 | CC | 28 | M | N | N | 4.5 | N | N | N/A | N | Roux, T |
| c.440G>A | W147* | 3 | CC | 53 | F | Y | Y | N/A | Y | Y | N/A | N | Roux, T |
| c.502C>T | L168F | 3 | CC | N/A | F | Y | N | N/A | Y | N/A | N/A | N/A | Roux, T |
| c.544C>T | R182* | 4 | CC | 65 | F | N/A | N | N/A | Y | N | N/A | N/A | Roux, T |
| c.665G>A | R222K | 5 | CC | 50 | M | Y | Y | N/A | N | Y | N/A | N | Roux, T |
| c.673C>T | R225* | 6 | U-box | 50 | F | Y | Y | 9 | N | N | N | N | Lieto, M |
| c.682C>T | P228S | 6 | U-box | 55 | M | Y | Y | N/A | Y | N | N | N/A | De Michele, G |
| c.682C>T | P228S | 6 | U-box | 35 | M | Y | Y | N/A | Y | Y | Y | N/A | De Michele, G |
| c.689_692delACCT | Y230Cfs*9 | 6 | U-box | 31 | M | Y | Y | 11 | N | Y | Y | Y | Lieto, M |
| c.689_692delACCT | Y230Cfs*9 | 6 | U-box | 48 | F | Y | Y | 36 | N | N | N | N | Lieto, M |
| c.694T>G | C232G | 6 | U-box | 44 | M | Y | N/A | 18 | Y | N | N | N | Ravel, J |
| c.694T>G | C232G | 6 | U-box | 68 | F | Y | Y | N/A | Y | N | N | N/A | Ravel, J |
| c.721C>T | R241W | 6 | U-box | 46 | F | N | Y | 3 | Y | N | N | N | Lieto, M |
| c.728C>T | P243L | 6 | U-box | 33 | F | Y | Y | N/A | N | N | N/A | Y | Roux, T |
| c.731_732delGC | C244Yfs*24 | 6 | U-box | 71 | N/A | Y | N/A | N/A | Y | N/A | N/A | N/A | Mol, M |
| c.731_732delGC | C244Yfs*24 | 6 | U-box | 66 | N/A | Y | Y | N/A | Y | N/A | N/A | N/A | Mol, M |
| c.731_732delGC | C244Yfs*24 | 6 | U-box | 72 | N/A | Y | Y | N/A | Y | N/A | N/A | N | Mol, M |
| c.731_732delGC | C244Yfs*24 | 6 | U-box | 69 | N/A | Y | Y | N/A | Y | N/A | N/A | N | Mol, M |
| c.731_732delGC | C244Yfs*24 | 6 | U-box | 65 | N/A | N | Y | N/A | Y | N/A | N/A | N | Mol, M |
| c.731_732delGC | C244Yfs*24 | 6 | U-box | 67 | N/A | Y | Y | N/A | Y | N/A | N/A | N | Mol, M |
| c.731_732delGC | C244Yfs*24 | 6 | U-box | 61 | N/A | Y | Y | N/A | Y | N/A | N/A | N | Mol, M |
| c.731_732delGC | C244Yfs*24 | 6 | U-box | 50 | N/A | Y | Y | N/A | Y | N/A | N/A | N | Mol, M |
| c.731_732delGC | C244Yfs*24 | 6 | U-box | 67 | N/A | Y | Y | N/A | Y | N/A | N/A | N | Mol, M |
| c.791_792delTG | V264Gfs*4 | 7 | U-box | 56 | M | Y | Y | 12 | N | N | N | N | Lieto, M |
| c.791_792delTG | V264Gfs*4 | 7 | U-box | 40 | F | Y | Y | 14 | N | N | N | N | Lieto, M |
| c.794G>A | G265D | 7 | U-box | 67 | F | Y | Y | 18.5 | Y | N | N/A | N | Roux, T |
| c.794G>A | G265D | 7 | U-box | 73 | M | Y | N/A | 24 | Y | N | N | Y | Ravel, J |
| c.818_819dupGC | P274Afs*3 | 7 | U-box | 42 | F | Y | Y | 40 | N | N | N | N | Lieto, M |
| c.818_819dupGC | P274Afs*3 | 7 | U-box | 34 | F | Y | Y | 9 | Y | Y | Y | N | Lieto, M |
| c.823_824delCT | L275Dfs*16 | 7 | U-box | 53 | F | Y | Y | N/A | Y | N | N | N | Genis, D |
| c.823_824delCT | L275Dfs*16 | 7 | U-box | 52 | F | Y | Y | N/A | Y | N | N | N | Genis, D |
| c.823_824delCT | L275Dfs*16 | 7 | U-box | 41 | F | Y | Y | N/A | Y | N | N | N | Genis, D |
| c.823_824delCT | L275Dfs*16 | 7 | U-box | 53 | M | N | N | N/A | Y | N | N | N | Genis, D |
| c.823_824delCT | L275Dfs*16 | 7 | U-box | 42 | F | Y | Y | N/A | Y | Y | Y | N | Genis, D |
| c.823_824delCT | L275Dfs*16 | 7 | U-box | 56 | M | N | N | N/A | Y | N | N | N | Genis, D |
| c.823_824delCT | L275Dfs*16 | 7 | U-box | N/A | F | Y | Y | N/A | Y | N | N | N | Genis, D |
| c.823_824delCT | L275Dfs*16 | 7 | U-box | 42 | F | Y | Y | N/A | Y | N | N | N | Genis, D |
| c.823_824delCT | L275Dfs*16 | 7 | U-box | 33 | M | N | N | N/A | N | N | N | N | Genis, D |
| c.823_824delCT | L275Dfs*16 | 7 | U-box | 30 | F | Y | Y | 12 | N | Y | Y | Y | Lieto, M |
| c.823_24delCT | L275Dfs*16 | 7 | U-box | 51 | F | Y | Y | N/A | Y | Y | Y | N/A | Palvadeau, R |
| c.824delT | L275Rfs*11 | 7 | U-box | N/A | F | N/A | N | N/A | N | N | N/A | N | Roux, T |
| c.849C>G | N283K | 7 | U-box | 48 | F | N/A | Y | N/A | N | N | N/A | N | Roux, T |

AOO = age of onset (in years), Dys = dysarthria, SARA = Scale for Assessment and Rating of Ataxia, CD = cognitive dysfunction, UMND = upper motor neuron dysfunction, ITR = increased tendon reflexes, Nys = nystagmus, N/A = not available.

## Supplementary Table 4. Primers used in CHIP Mutagenesis.

| **Primer Name** | **Sequence** |
| --- | --- |
| CHIP G33S-F | CAAGGAGCAGAGCAATCGTCTGTTCGTGGG |
| CHIP G33S-R | AGCTCCTGCGCGCTCGGG |
| CHIP P228S-F | GCGAGACATCTCCGACTACCT |
| CHIP P228S-R | TTCTTCCTCTTCTCATCCACC |
| CHIP L275Dfs*16-F1 | GACCCAGGAACAGCTCATCCCCAAC |
| CHIP L275Dfs*16-R1 | GGGGCTCCGGGTCACGGG |
| CHIP L275Dfs*16-F2 (pcDNA3) | TATCTAGAGGGCCCGCGG |
| CHIP L275Dfs*16-R2 (pcDNA3) | ATAACCTCCTTCATAGCCAAGTTGG |
| CHIP L275Dfs*16-F2 (pET-30) | ATAACCTCCTTCATAGCC |
| CHIP L275Dfs*16-R2 (pET-30) | AGTCTAGACCTCGAGCAC |
| CHIP F37L-F | TTCGGCCCACCAACAGACGATTGCCC |
| CHIP F37L-R | AGTACCCGGAGGCGGCGG |
| CHIP I53T-F | GTTCCGGGTGGTCGCGCGGCC |
| CHIP I53T-R | CCGCTGGTGGCCGTGTATTACACC |
| CHIP P57L-F (pET-30) | GGCCACCAGCAGGTTCCGGGT |
| CHIP P57L-R (pET-30) | GTGTATTACACCAACCGGGCC |
| CHIP P57L-F (pcDNA3) | ACCCGGAACCTGCTGGTGGCC |
| CHIP P57L-R (pcDNA3) | GATCGCGCGGCCGTAGCAG |
| CHIP A67T-F (pET-30) | TAGCACAAGGTCCGGTTGGTG |
| CHIP A67T-R (pET-30) | CCTGAAGATGCAGCAGCAC |
| CHIP A67T-F (pcDNA3) | CACCAACCGGACCTTGTGCTA |
| CHIP A67T-R (pcDNA3) | TAATACACGGCCACCAGC |
| CHIP P225*-F (pET-30) | CTTCTTCCTCTTCTCATC |
| CHIP P225*-R (pET-30) | GAGGACTACAGTCTAGAC |
| CHIP P225*-F (pcDNA3) | TATCTAGAGGGCCCGCGG |
| CHIP P225*-R (pcDNA3) | CTTCTTCCTCTTCTCATCCACCTG |
| CHIP Y230Cfs*9-F1 | AGTCGGGGATGTCTCGCT |
| CHIP Y230Cfs*9-R1 | GTGTGGCAAGATCAGCTTTG |
| CHIP Y230Cfs*9-F2 (pET-30) | GCTCAAAGCTGATCTTGC |
| CHIP Y230Cfs*9-R2 (pET-30) | GAGGACTACAGTCTAGAC |
| CHIP Y230Cfs*9-F2 (pcDNA3) | TATCTAGAGGGCCCGCGG |
| CHIP Y230Cfs*9-R2 (pcDNA3) | GCTCAAAGCTGATCTTGCCAC |
| CHIP R241W-F (pET-30) | CACGGCTCCCACATCAGCTCAAAG |
| CHIP R241W-R (pET-30) | CATCACGCCCAGTGGCAT |
| CHIP R241W-F (pcDNA3) | TGAGCTGATGTGGGAGCCGTG |
| CHIP R241W-R (pcDNA3) | AAGCTGATCTTGCCACACAGG |
| CHIP C244Yfs*24-F1 | ACGGCTCCCGCATCAGCT |
| CHIP C244Yfs*24-R1 | ATCACGCCCAGTGGCATC |
| CHIP C244Yfs*24-F2 (pET-30) | AAATGACCCACACGCTGC |
| CHIP C244Yfs*24-R2 (pET-30) | GAGGACTACAGTCTAGACC |
| CHIP C244Yfs*24-F2 (pcDNA3) | TATCTAGAGGGCCCGCGG |
| CHIP C244Yfs*24-R2 (pcDNA3) | AAATGACCCACACGCTGC |
| CHIP V264Gfs*4-F1 | CACGCTGCAGGTGCTCCT |
| CHIP V264Gfs*4-R1 | GGTCATTTTGACCCCGTGACC |
| CHIP V264Gfs*4-F2 (pET-30) | AAAATGACCCACGCTGCA |
| CHIP V264Gfs*4-R2 (pET-30) | GAGGACTACAGTCTAGACC |
| CHIP V264Gfs*4-F2 (pcDNA3) | TATCTAGAGGGCCCGCGG |
| CHIP V264Gfs*4-R2 (pcDNA3) | AAATGACCCACGCTGCAG |
| CHIP P274Afs*3-F1 (pET-30) | GCGCTCCGGGTCACGGGGTC |
| CHIP P274Afs*3-R1 (pET-30) | CCCCTGACCCAGGAACAGC |
| CHIP P274Afs*3-F2 (pET-30) | GGGGGCGCTCCGGGTCAC |
| CHIP P274Afs*3-R2 (pET-30) | GAGGACTACAGTCTAGACCTCGAGCACC |
| CHIP P274Afs*3-F1 (pcDNA3) | GCCCCCTGACCCAGGAACAGC |
| CHIP P274Afs*3-R1 (pcDNA3) | GCTCCGGGTCACGGGGTC |
| CHIP P274Afs*3-F2 (pcDNA3) | TATCTAGAGGGCCCGCGGTTCG |
| CHIP P274Afs*3-R2 (pcDNA3) | GGGGGCGCTCCGGGTCAC |

## Supplementary Table 5. Primary antibodies used for immunoblotting and immunofluorescence.

| **Antibody** | **Source** | **Identifier** |
| --- | --- | --- |
| Myc-HRP | Cell Signaling Technologies | 2040 |
| Flag-HRP | Cell Signaling Technologies | 2044 |
| HA | Cell Signaling Technologies | 3724 |
| HSF1 | Enzo Life Sciences | ADI-SPA-901 |
| HSF1 | Cell Signaling Technologies | #4356 |
| b-tubulin | Sigma | T3828 |
| Lamin B1-HRP | Cell Signaling Technologies | 15068 |
| Mouse Myc | Proteintech | 60003-2-Ig |
| PhosphoCHIP S20 | AbMart Inc.: p-CHIP Ser20* | 25011–1 |
| StarBright B700 | Bio-Rad | 12004161 |
| StarBright B520 | Bio-Rad | 12005866 |

*(Custom antibody, Shanghai, China)

## Supplementary Table 6. Full unfiltered g:Profiler multiquery enrichment results (JBC-D-26-00078_Supplemental_Table_6.csv)

The table contains results from g:Profiler (g:GOSt) multiquery analysis performed on three gene sets derived from RNA-seq:

- **Overlap** (313 genes commonly differentially expressed in both shControl and shSTUB1 cells upon heat shock)
- **Control** (573 genes uniquely differentially expressed in shControl cells)
- **STUB1** (110 genes uniquely differentially expressed in shSTUB1 cells)

Analyses were performed against the human (hsapiens) background using Ensembl release 105 (or 109, depending on run). Only terms with FDR-adjusted p-value < 0.05 in at least one query are shown. The full dataset is provided as a separate file: JBC-D-26-00078_Supplemental_Table_6.csv.

**Column descriptions:**

- **source**: Ontology or database source (e.g., GO:MF, GO:BP, GO:CC, REAC, KEGG, etc.).
- **term_name**: Descriptive name of the enriched term/pathway.
- **term_id**: Unique identifier for the term (e.g., GO:0006355).
- **term_size**: Total number of genes annotated to this term in the background.
- **effective_domain_size**: Number of genes in the effective statistical domain (background after filtering).
- **highlighted**: Flags (true/false) indicating whether the term met significance criteria in each query (overlap, control, STUB1).
- **adjusted_p_value__overlap** / **adjusted_p_value__control** / **adjusted_p_value__STUB1**: FDR-adjusted p-values for each gene set.
- **query_size__overlap** / **query_size__control** / **query_size__STUB1**: Number of input genes in each respective gene set.
- **intersection_size__overlap** / **intersection_size__control** / **intersection_size__STUB1**: Number of genes in the input set that overlap with the term.

# SUPPLEMENTAL METHODS

## Nuclear and Cytoplasmic Fractionation

COS-7 cells were maintained in Dulbecco’s Modified Eagle’s Medium (DMEM; Corning, 10-017-CV) supplemented with 10% fetal bovine serum (FBS; Gibco, 26140-079) and 1% penicillin-streptomycin at 37 °C in a humidified 5% CO₂ incubator. For transient transfection, cells were seeded to reach ~70–80% confluence and transfected with plasmid DNA using X-tremeGENE™ 9 DNA transfection reagent (Roche, 6365787001) at a 1:3 DNA-to-reagent ratio according to the manufacturer’s instructions. Approximately 24 hours after the transfection, nuclear and cytoplasmic extracts were prepared using the NE-PER Nuclear and Cytoplasmic Extraction Kit (Thermo Scientific, 78835) following the manufacturer’s protocol. Briefly, cells were harvested, lysed sequentially to separate cytoplasmic and nuclear components, and fractions were collected for downstream analyses.

# SUPPLEMENTAL FIGURE LEGENDS

## Supplementary Figure 1. Structural modeling of CHIP variants reveals steric clashes at multiple mutation sites.

The crystal structure of wild-type CHIP (WT, grey) is shown as a cartoon representation with key side chains depicted as sticks. The native residues at positions 33 (glycine), 67 (alanine), 153 (isoleucine), 228 (proline), and 241 (arginine) are highlighted in cyan. Substitution of these residues with serine (G33S and P228S), threonine (A67T and I153T), or tryptophan (R241W) introduces new side-chain conformations (rotamers) shown as g⁺ (yellow), g⁻ (green), and t (magenta). Colored "bump" discs provide a qualitative indication of van der Waals interactions between mutant side chains and surrounding residues, binned as slight contact (green), slight overlap (orange), or significant overlap (red), with disc size proportional to the degree of steric interference.

## Supplementary Figure 2. Mass photometry reveals distinct oligomerization profiles for CHIP wild-type and SCA48-associated variants.

We used mass photometry (MP) to monitor the oligomerization behavior of wild-type (WT) CHIP and SCA48-associated variants, using characteristic CHIP peaks corresponding to dimer, tetramer, hexamer, and octamer molecular weights. According to their MP profiles, CHIP mutants were grouped into three categories, with relative quantification represented by dot plot summarized by the mean bar plot ± SD (n = 3): *Dimer-repeating units:* (**A**) Representative MP profiles and (**B**) quantification of mutations G33S, F37L, I53T, P57L, A67T, and R241W, which retain distinct oligomeric states at the dimer, tetramer, hexamer, and octamer levels. *Dimer-only:* (**C**) MP profiles and (**D**) quantification for R225* and Y230Cfs*9* variants, which predominantly (~80%) form dimers without detectable higher-order oligomers*.* *Non-discrete high molecular weight species:* (**E**) MP profiles and (**F**) quantification of synthetic mutations P228S, C244Yfs24, V264Gfs4, P274Gfs3, and L275Dfs*16, which exhibit very low levels of discrete oligomers and instead accumulate as non-discrete high-molecular-weight species.

## Supplementary Figure 3. Oligomerization profiles of CHIP at different concentrations.

Mass photometry experiments were repeated with WT and one representative mutant from each class at three concentrations: 20 nM (*upper*), 50 nM (*middle*), and 100 nM (*lower*). For each concentration, probability density (PD) is plotted against the molecular weight (kDa) (*left*). The proportions of CHIP oligomeric states, dimer (2n), tetramer (4n), hexamer (6n), octamer (8n), and high molecular weight (High MW), are represented by a dot plot summarized by the mean bar plot ± SD (n = 2)*.*

## Supplementary Figure 4. Binding affinity and function of UBE2D2 with CHIP variants.

(**A**) Affinity values (µM) determined by bio-layer interferometry*.* Results shown as the mean ± SD (n = 10). Each point represents the determined affinity (K_D_) from individual experiments. (**B**) Representative gel images of CHIP-dependent ubiquitination of fluorescently labeled HSP70, with its ubiquitination denoted by HSP70-Ub_n_. Concentration of UBE2D2 was titrated at 5 µM (*upper*), 25 µM (*middle*), and 100 µM (*lower*) with constant CHIP and HSP70.

## Supplementary Figure 5. Impact of SCA48 mutations on CHIP phosphorylation, vector control immunofluorescence, and subcellular localization of CHIP variants and HSF1 determined by fractionated immunoblotting.

(**A**) Representative immunoblot of pS19, myc-CHIP, and SF. (**B**) Relative pS19 dot plot (mean ± SD, n = 5); one-sample t-test, * P < 0.05. (**C**) Representative immunofluorescence of pcDNA3-transfected (vector control) COS-7 at 37 °C and 42 °C: HSF1 (gray), DAPI (blue); dashed boxes mark single-channel insets. (**D**) Fractionated immunoblotting of COS-7 cells expressing CHIP wild type (WT), G33S, P228S, L275Dfs16, and the synthetic mutant K30A revealed distinct nuclear and cytoplasmic distributions of CHIP and HSF1. CHIP WT was predominantly cytoplasmic, but its overexpression enhanced nuclear localization of HSF1. The G33S, P228S, and K30A variants showed a mild reduction in nuclear CHIP compared to WT, whereas the L275Dfs16 mutant markedly increased nuclear accumulation of both CHIP and HSF1. CHIP WT overexpression increased HSF1 nuclear levels, which were generally retained, but not elevated, by most CHIP variants, with a remarkable increase observed for L275Dfs16.

## Supplementary Figure 6. Validation of *STUB1* knockdown, HSF1 activation, and RNA-seq quality control.

(**A**) Immunoblot analysis of CHIP protein levels in HEK-293 cells transduced with individual MISSION shRNA lentiviral particles (1–4: TRCN0000007525, TRCN0000007526, TRCN0000007528, TRCN0000007529) or a non-targeting control shRNA (shCtrl). (**B**) Immunoblot analysis of CHIP and HSF1 in whole-cell lysates, cytoplasmic, and nuclear-enriched fractions from shCtrl or sh*STUB1* cells. MEK and HP1α served as cytoplasmic and nuclear loading markers, respectively. Cells were maintained at 37 °C or subjected to heat shock at 42 °C for 30 min (+). (**C**) qPCR quantification of *STUB1* (CHIP) and *HSPA1A* (HSP70) mRNA levels in shCtrl and sh*STUB1* cells under basal (37 °C) or heat-shock (42 °C) conditions. Data are presented as box plots showing individual biological replicates (n = 3 per condition). Statistical analysis was performed using two-way ANOVA followed by Tukey’s multiple-comparison post-hoc test; pairwise P values are indicated. (D) Co-immunoprecipitation (IP) of CHIP-myc tag, along with input levels of both transgenes visualized by immunoblot, from COS-7 cells, expressing the specified CHIP variants, under basal (37 °C) or heat-shock (42 °C) conditions.

## Supplementary Figure 7. Relationship between CHIP mutation domain and clinical severity in SCA48 patients.

Analysis of clinical data from 87 SCA48 patients with confirmed STUB1 mutations compiled from previously published case studies. The Scale for the Assessment and Rating of Ataxia (SARA) scores (when available; n = 27) were plotted by mutation location within the CHIP domains (TPR vs. U-box). Statistical comparison between the two groups was performed using an unpaired t-test. The number of data points and p-values are indicated in the bottom left corner of each plot. No significant difference in mean SARA scores was observed between patients carrying TPR or U-box domain mutations, suggesting that the mutation domain alone does not account for variability in disease severity.
